# Surface-Water Nutrient Conditions and Sources in the United States Pacific Northwest^1^

**DOI:** 10.1111/j.1752-1688.2011.00580.x

**Published:** 2011-10

**Authors:** Daniel R Wise, Henry M Johnson

**Keywords:** nutrients, watersheds, geospatial analysis, Pacific Northwest

## Abstract

**Abstract:**

The SPAtially Referenced Regressions On Watershed attributes (SPARROW) model was used to perform an assessment of surface-water nutrient conditions and to identify important nutrient sources in watersheds of the Pacific Northwest region of the United States (U.S.) for the year 2002. Our models included variables representing nutrient sources as well as landscape characteristics that affect nutrient delivery to streams. Annual nutrient yields were higher in watersheds on the wetter, west side of the Cascade Range compared to watersheds on the drier, east side. High nutrient enrichment (relative to the U.S. Environmental Protection Agency's recommended nutrient criteria) was estimated in watersheds throughout the region. Forest land was generally the largest source of total nitrogen stream load and geologic material was generally the largest source of total phosphorus stream load generated within the 12,039 modeled watersheds. These results reflected the prevalence of these two natural sources and the low input from other nutrient sources across the region. However, the combined input from agriculture, point sources, and developed land, rather than natural nutrient sources, was responsible for most of the nutrient load discharged from many of the largest watersheds. Our results provided an understanding of the regional patterns in surface-water nutrient conditions and should be useful to environmental managers in future water-quality planning efforts.

## Introduction

One of the most important water-quality issues in the United States (U.S.) Pacific Northwest (PNW) is the enrichment of many freshwater streams with nutrients. State regulatory agencies have designated many freshwater stream reaches in the region as water-quality impaired because of high concentrations of nutrients and related impacts, such as excessive aquatic plant growth, low concentrations of dissolved oxygen, and high pH (see Figure S1 and Table S1 in the Supporting Information). The methodologies for assessing the impacts from nutrient enrichment, however, vary between states and often within the same state (Keller and Cavallaro, 2007). In addition to the regulatory assessments of surface-water nutrients there have also been some nonregulatory assessments of individual watersheds in the PNW (e.g., Rupert, 1996; Clark, 1997; Embrey and Inkpen, 1998; Wentz *et al.*, 1998; Williamson *et al.*, 1998; Ebbert *et al.*, 2003; Wise *et al.*, 2007), but there has been no evaluation of nutrient enrichment across the entire region. Water-quality managers in the PNW, therefore, would benefit from a systematic and consistent regional assessment of surface-water nutrient enrichment and from an estimate of the relative contribution of different sources to surface-water nutrients. Water-quality modeling can be used to address this need by using available data to characterize stream conditions.

The U.S. Geological Survey (USGS) SPAtially Referenced Regressions On Watershed attributes (SPARROW) model is a hybrid statistical and mechanistic model for estimating the movement of mass through the landscape under long-term, steady state conditions (Schwarz *et al.*, 2006; Preston *et al.*, 2009). The model uses watershed data describing sources, landscape characteristics, and stream properties to explain the spatial variation in measured, mean annual stream load. One advantage SPARROW has over most other source-transport watershed models is that it uses a spatially referenced stream network covering a large region together with simple process-based descriptions of sources and transport (Schwarz *et al.*, 2006). The SPARROW model has been used at different scales (e.g., Smith *et al.*, 1997; Preston and Brakebill, 1999; Moore *et al.*, 2004; Hoos and McMahon, 2009) to assess nutrient enrichment and to estimate the contribution of different nutrient sources to sensitive downstream locations. The Supporting Information provides more details about the SPARROW model.

The USGS National Water-Quality Assessment (NAWQA) Program is currently developing SPARROW models for major river basins of the conterminous U.S. This paper describes the PNW component of that effort; specifically, the development of SPARROW models to assess the factors that affect total nitrogen (TN) and total phosphorus (TP) conditions in the surface waters of the PNW. The objectives of our assessment were to: (1) calibrate PNW SPARROW models for TN and TP, (2) estimate mean annual TN and TP yields, (3) use a regionally consistent method to estimate nutrient enrichment, and (4) determine the contribution of different nutrient sources to TN and TP stream loads.

## Description of the PNW Modeling Region

**FIGURE 1:**
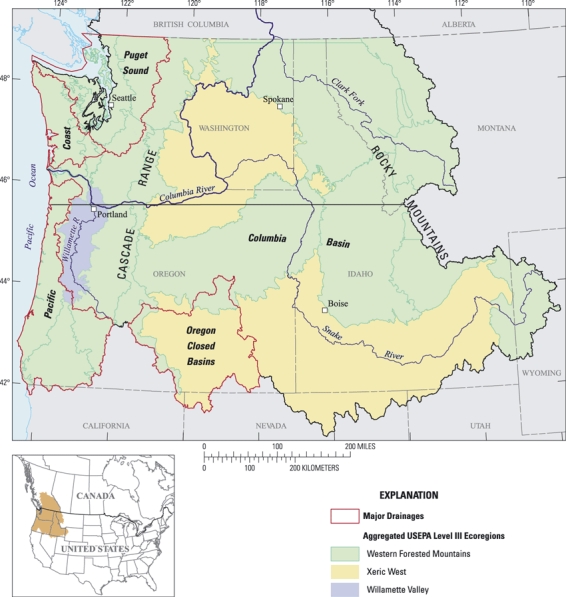
Major Drainages, Largest Rivers, and Aggregated USEPA Level III Ecoregions in the United States Pacific Northwest.

The PNW covers approximately 814,000 km^2^ and includes five major drainages: the Columbia River basin, the Puget Sound (PUGT) basin, the Pacific coast of Washington, the Pacific coast of Oregon, and the closed basins in southern Oregon (Figure 1). Eighty-seven percent of the total area of the PNW is within the conterminous U.S. and the remainder is in Canada. The U.S. part of the PNW includes three aggregated U.S. Environmental Protection Agency (USEPA) level III ecoregions (USEPA 1997): Western Forested Mountains (61%), the Xeric West (36%), and the Willamette Valley (3%). In 2001, range and forest land made up 43 and 40%, respectively, of the U.S part of the PNW, agriculture accounted for 10%, and developed and other landscape types made up the rest (Homer *et al.*, 2004). The PNW is characterized by distinct spatial and seasonal precipitation patterns. The mountain and valley systems that define the landscape have a pronounced effect on regional precipitation, which mostly falls during the late autumn, winter, and early spring. Most of the precipitation falls in the coastal mountains (primarily as rain) and in the Cascade Range and the Rocky Mountains (primarily as snow), whereas the least falls in the region between the Cascade Range and the Rocky Mountains. The land between the coastal mountains and Cascade Range contains the largest metropolitan areas (including Seattle and Portland), as well as agricultural areas that benefit from a mild climate, abundant rainfall, and fertile soil. Many smaller communities also lie along the Pacific coasts of Washington and Oregon. The other areas of the PNW are generally sparsely populated and contain agricultural areas that require extensive irrigation to supplement the small amount of rain that falls east of the Cascade Range.

## Methods

### Data Compilation

#### Calibration Stream Loads

Water-quality and streamflow data were needed to estimate the mean annual TN and TP stream loads for the calibration stations used in the PNW SPARROW models (178 for TN and 228 for TP). These data were obtained from federal agencies (primarily the USGS), state regulatory agencies, county and city governments, and conservation districts (Saad *et al.*, this issue). The sources of water-quality and streamflow data used to estimate the mean annual TN and TP stream loads for the calibration stations are shown in Table 1. Using water-quality and streamflow data from organizations other than the USGS allowed us to have calibration sites on stream reaches where the USGS does not collect data. We did not review the field and laboratory quality assurance programs for these organizations, however, and the data were often collected in ways that differed from data collected by the USGS (see Wise *et al.*, 2007). The mean annual TN and TP stream loads were estimated using the USGS Fluxmaster model (Schwarz *et al.*, 2006), which relates the loads measured at water-quality monitoring stations to measured streamflow, season, and time. The mean stream loads estimated by Fluxmaster were detrended to a base year (2002 for our analysis) to account for differences in record length, hydrologic conditions, and sample size between calibration stations (Preston *et al.*, 2009). Therefore, the results presented in this paper reflect 2002 landscape nutrient loadings but long-term average hydrologic conditions.

**TABLE 1:** Sources of Data Used to Estimate Calibration Stream Loads for the SPARROW Models Developed for the United States Pacific Northwest

Water-Quality Sites					Streamflow Sites		
	
Level	Agency	Number	Location	Number	Level	Agency	Number
Federal	U.S. Geological Survey	87	Washington	86	Federal	U.S. Geological Survey	206
	Bureau of Reclamation	19	Oregon	73		Bureau of Reclamation	16
	U.S. Environmental Protection Agency	4	Idaho	56	State	Oregon Water Resources Department	2
State	Oregon Department of Environmental Quality	60	Montana	10	Local	King County, Washington	6
	Washington Department of Ecology	53	Wyoming	4	Total		230
	Nevada Department of Environmental Quality	1	Nevada	1			
Local	King County, Washington	4	Total	230			
	Lincoln County Conservation District, Washington	1					
	Snohomish County, Washington	1					
Total		230					

Note: SPARROW, SPAtially Referenced Regressions On Watershed attributes.

#### Stream Reach Attributes

For this analysis, we used the 1:500,000 scale Enhanced River Reach File 2 (ERF1-2) to represent the stream network for the PNW. An incremental watershed was delineated for each of the 12,039 ERF1-2 stream reaches in the PNW from 1-km digital elevation models (Nolan *et al.*, 2002; Brakebill *et al.*, this issue). The incremental watershed for a reach is the area that drains directly to the reach without passing through another reach. The incremental watersheds were generally smaller on the west side of the Cascades (<1 to 618 km^2^ with an average size of 38.9 km^2^) compared to those on the east side (<1 to 9910 km^2^ with an average size of 66.5 km^2^).

Stream attenuation (removal) of nutrients through particulate settling, algal uptake, benthic denitrification (for nitrogen), or other natural processes is an important control on the transport of nutrients through large river basins (Alexander *et al.*, 2000). In the SPARROW model stream attenuation is estimated using a first-order decay process that is a function of the time of travel for each reach (reach length divided by estimated mean annual velocity) within different streamflow classes. The reservoir decay term in SPARROW represents the net effect of various processes that remove nutrients (e.g., particulate settling, algal uptake, and benthic denitrification) or add nutrients (e.g., nitrogen fixation and mineralization, phosphorus dissolution and resuspension) to surface water in an impoundment. Reservoir decay was modeled as an apparent settling velocity that was expressed in units of length per time and was a function of the areal hydraulic load (estimated mean annual streamflow through an impoundment divided by surface area). The reach length, mean annual velocity, mean annual streamflow, and impoundment area were attributes of the ERF1-2 stream network (Brakebill *et al.*, this issue).

Although the ERF1-2 stream network included the minimum reach attributes required for the SPARROW model, additional information was needed to account for the irrigation employed in much of the PNW during the growing season. This irrigation commonly requires major modifications in natural streamflow, and these modifications had to be included in our SPARROW models to properly estimate nutrient transport through the surface waters of the PNW. The SPARROW model includes a reach attribute that allows the user to simulate the diversion of streamflow in a hydrologic network. This is done by estimating the fraction of streamflow and, therefore, nutrient load, that is delivered from one reach to the reach immediately downstream. Nutrient load that is removed in this way is not returned to the stream network. A detailed description of the methodology used to estimate these fractional diversions is provided in the Supporting Information.

Each ERF1-2 reach in the PNW was assigned the recommended USEPA nutrient criteria for TN and TP for that reach. These recommended nutrient criteria were established on a regional scale to protect surface waters from the negative effects of nutrient enrichment (USEPA, 2000). The recommended nutrient criteria (Table 2) are equal to the 25th percentile of all available concentrations for each aggregated level III ecoregion and are intended to represent reference (i.e., minimally impacted) conditions for each ecoregion. Although the recommended nutrient criteria are not water-quality standards, they are intended to be starting points for states and tribes to set their own standards. Also, because they are determined in a consistent manner, the recommended nutrient criteria are useful for estimating nutrient enrichment across a large region like the PNW. Each ERF1-2 reach also was identified as being water-quality impaired (or not) due to nutrient enrichment, based on the most recent state 303(d) lists. These lists, which are submitted biannually by governing jurisdictions (states, territories, or covered tribal entities) to the USEPA, contain water bodies that are not meeting their designated uses. As the stream reaches on the 303(d) lists were not initially referenced to the ERF1-2 stream network, we needed to complete this task. This was done through name matching, location matching, and onscreen digitization. Table S1 in the Supplemental Information lists the parameters used by each state to assess nutrient enrichment.

**TABLE 2:** Recommended USEPA Total Nitrogen (TN) and Total Phosphorus (TP) Criteria for Aggregated Level III Ecoregions in the United States Pacific Northwest

	Nutrient	
	
Aggregated Level III Ecoregion	TN	TP
Willamette and Central Valleys	0.31	0.047
Western Forested Mountains	0.12	0.010
Xeric West	0.38	0.022

Notes: USEPA, U.S. Environmental Protection Agency.

Values are in mg/l. The recommended nutrient criteria are equal to the 25th percentile of all available concentrations for each aggregated level III ecoregion and are intended to represent reference (i.e., minimally impacted) conditions for each ecoregion.

#### Watershed Attributes

Nutrient source inputs considered in the modeling included discharge from permitted wastewater treatment facilities (point sources) and leaching from domestic septic tanks, runoff from developed and forest land, wet deposition of inorganic nitrogen, application of fertilizer and livestock manure to agricultural land, leaching from nitrogen fixing plants, and weathering of phosphorus from geologic material. The delivery of nutrients from land to water was modeled by considering climate, soil properties, geology, watershed hydrology (infiltration, groundwater recharge, and runoff), hydrologic manipulation for irrigation, and hydrologic landscape regions. Hydrologic landscape regions are groups of contiguous watersheds that share similar landscape and climate characteristics that represent factors assumed to affect hydrologic processes in the environment (Wolock, 2003).

The nutrient source and watershed properties used in the PNW SPARROW models were summarized for each one of the incremental watersheds, either as part of a national geospatial dataset (Wieczorek and Lamotte, 2011) or specifically for the PNW. Point-source nutrient loads for 2002 were estimated by multiplying measured discharge by source-specific or regionally averaged, industry-specific TN and TP concentrations (Maupin and Ivahnenko, this issue). Estimates of septic tank use were based on 1990 U.S. census data (U.S. Census Bureau, 1990). The areas of developed and forest land within each watershed were obtained from the National Land Cover Database (Homer *et al.*, 2004). The annual nitrogen loading from wet deposition of inorganic nitrogen was estimated from data obtained from the National Atmospheric Deposition Program (Wieczorek and Lamotte, 2011). The annual nitrogen and phosphorus loadings from fertilizer use and livestock waste were estimated by disaggregating county-level estimates to the agricultural land within each county (Ruddy *et al.*, 2006). Estimates of red alder distribution for western Oregon and Washington were obtained from the Landscape, Ecology, Modeling, Mapping and Analysis (LEMMA) project (LEMMA, 2008 for Oregon; Mathew Gregory, Oregon State University, written communication, September 8, 2008 for Washington). Estimates of the concentration of phosphorus in geologic materials (on a mass per mass basis) were based on measurements made by the National Geochemical Survey of the concentration of phosphorus in the bed sediment of streams that were minimally impacted by anthropogenic sources (USGS, 2004). The average value for each watershed was multiplied by the watershed area (km^2^) to obtain an index representing phosphorus contained in geologic material. Effective mean annual precipitation (30 year mean) for each watershed was estimated by summing the mean depth of local precipitation plus the depth of irrigation water. The values for local precipitation were part of a nationally compiled dataset and the depth of irrigation water was estimated by multiplying the percentage of irrigated agriculture in each watershed by 91 cm, which is a typical depth of irrigation water applied annually to crops located in arid areas of the PNW (NRCS, 1997).

Runoff from developed land mostly represented the export of nutrients in urban stormwater, but also other minor sources associated with urbanized areas such as onsite sewage systems (WRI, 2011). Runoff from forest land mostly represented the export of naturally occurring nitrogen (e.g., nitrogen that was fixed from the atmosphere by soil bacteria and introduced into the terrestrial ecosystem), but also nitrogen inputs from applying fertilizer to public and private timberlands. While some nitrogen export from forest land is due to atmospheric deposition, this was included in the model as a separate source. Although fertilization is used on a small area of the forest land in the PNW and applications are infrequent compared to agricultural crops, it has been shown to be an important source of nitrogen to some stream reaches in the Cascades and the Washington and Oregon Coast Ranges (Chappell *et al.*, 1991; Bisson *et al.*, 1992; Anderson, 2002). The forests on the east side of the Cascades were modeled separately from the forests on the west side because the drier east-side forests generally have less biomass and greater species diversity than the wetter west-side forests (del Moral and Fleming, 1979). We considered red alder trees (*Alnus rubra*) as a distinct nitrogen source term separate from forest land. Red alder is a fast-growing deciduous tree that is common in repeatedly disturbed areas in the low-elevation forests of western Oregon and Washington, and is unique among major tree species in the PNW in its ability to symbiotically fix atmospheric nitrogen (Harrington, 2006). As a result, red alder plays an important role in the export of nitrogen from many watersheds in the region (Compton *et al.*, 2003).

Although complete landscape data were not available for many watersheds included in the PNW models because they were partly located in Canada, we were still able to estimate stream loads entering the study area from these watersheds. The measured mean annual stream loads discharged from three of these watersheds (the Canadian portions of the Columbia River, Similkameen River, and Nine Mile Creek) were used as boundary conditions for the model because they corresponded to monitoring stations near the border. The stream load discharged from unmonitored cross-border watersheds where <50% of the area was in Canada was estimated by extrapolating the landscape data from the available U.S. datasets to the entire watershed. The stream load discharged from unmonitored cross-border watersheds where 50% or more of the area was in Canada was estimated by having SPARROW estimate a coefficient for watershed area, which was the only source term for these watersheds.

The Supporting Information includes additional details about the watershed attributes used in our models. The input data that were used in the calibration of the PNW SPARROW models can be obtained from the project web page (http://or.water.usgs.gov/sparrow/).

### Data Analysis

The coefficients included in our calibrated SPARROW models represented statistically significant or otherwise important geospatial variables. The statistical significance of each source coefficient (which was constrained to be positive) was determined by using a one-sided *t*-test and a significance level of 0.10. The statistical significance of each delivery coefficient (which could have been positive or negative, reflecting either limited or enhanced delivery) was determined by using a two-sided *t*-test and a significance level of 0.05. The streamflow classes used to model stream attenuation were chosen by repeatedly running the SPARROW model until significant stream attenuation coefficients were obtained with minimum model error. The statistical significance of the coefficients representing stream attenuation and reservoir removal (which was constrained to be positive) was determined by using a one-sided *t*-test and a significance level of 0.05.

The SPARROW model employs a weighted nonlinear least squares (NLLS) regression to estimate model coefficients (Schwarz *et al.*, 2006) and provides a way to correct for bias and assess uncertainty in these estimated coefficients. Because of the nonlinear manner in which the estimated coefficients enter the model, this bias and uncertainty needs to be evaluated using a bootstrap resampling method (Schwarz *et al.*, 2006). The method is implemented through repeated estimation of the SPARROW model (200 times for our application) to obtain a range of values for each coefficient, from which a mean value (the nonparametric bootstrap estimate) is estimated. The overall stability of each of our models was evaluated by comparing the NLLS estimates of the model coefficients to the nonparametric bootstrap estimates. The 90% confidence intervals for the NLLS coefficients in each model were generated by using the standard errors and a *t*-distribution with *N*− *k* degrees of freedom, where *N* was the number of calibration sites and *k* was the number of coefficients. The goodness of model fit was evaluated by calculating the *R*^2^, yield *R*^2^, root mean squared error (RMSE), and the model *p*-value. The yield *R*^2^ is the *R*^2^ value for the natural logarithm of yield, and is considered a better measure of goodness of fit than *R*^2^ because it accounts for the effect of contributing area (which can explain much of the variation in stream load).

The SPARROW model was used to predict the mean annual incremental yield for each of the 12,039 modeled watersheds. Incremental yield is equal to the stream load per unit area that is attributable to landscape features (sources, delivery mechanisms, and attenuation processes) exclusively within a watershed, and is a useful tool for comparing the relative intensity of stream load between watersheds because it normalizes for contributing area. SPARROW was also used to predict the mean annual concentrations of TN and TP for each ERF1-2 stream reach. Mean annual concentrations were calculated by dividing the predicted mean annual stream load in each reach by the estimated mean annual streamflow. We calculated the probabilities that the mean annual concentrations of TN and TP in each ERF1-2 stream reach were greater than the applicable USEPA reference criteria. This value, however, applied only to mean annual concentrations and was not the probability that any single concentration measurement in a particular reach would be greater than a reference criterion. The probability that the predicted mean annual concentration in a reach exceeded a reference criterion was given by 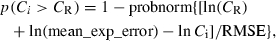
(1)

where *C*_*i*_ is the predicted mean annual concentration (mg/l); *C*_R_ is the USEPA reference criterion (mg/l); *p*(*C*_i_ > *C*_R_) is the probability that the *C*_*i*_ is greater than *C*_R_; probnorm{.} is the probability that an observation from the standard normal distribution is less than or equal to the value in the brackets; mean_exp_error is the mean of the exponentially transformed model residuals, which is used to correct for retransformation bias associated with model error in converting results in natural logarithm space to linear space; and RMSE is the root mean squared error from the model calibration which, when multiplied by 100, can be interpreted as the one standard deviation percent error associated with an estimation for any single reach.

An estimate of TN and TP enrichment for each eight-digit hydrologic unit code (HUC8) watershed was made by summing the length of all reaches with more than a 90% probability that the mean annual concentration of TN or TP exceeded the reference criterion and dividing by the total length of stream reaches in the watershed. Very high enrichment corresponded to a ratio >0.75, high enrichment corresponded to a ratio between 0.50 and 0.75, low enrichment corresponded to a ratio between 0.25 and 0.50, and very low enrichment corresponded to a ratio <0.25. We also compared these SPARROW-based estimates of nutrient enrichment in PNW HUC8 watersheds to the prevalence of state 303(d) listings.

## Results

### Calibration Results

**FIGURE 2:**
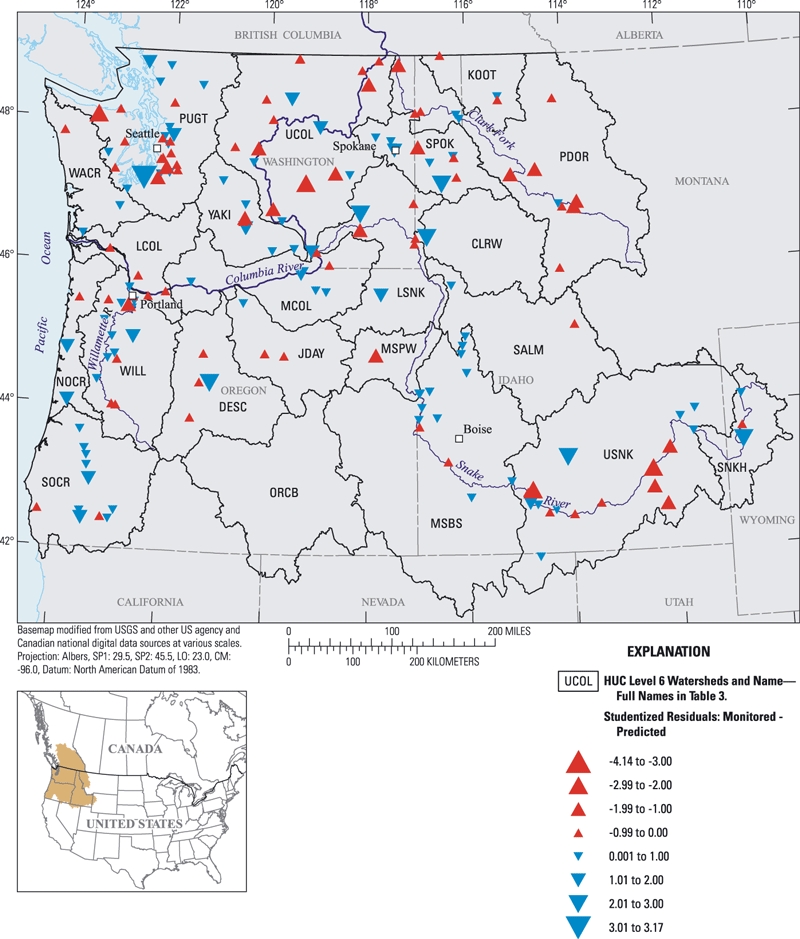
Spatial Distribution of Residual Stream Load for the Total Nitrogen SPARROW Model Developed for the United States Pacific Northwest.

**FIGURE 3:**
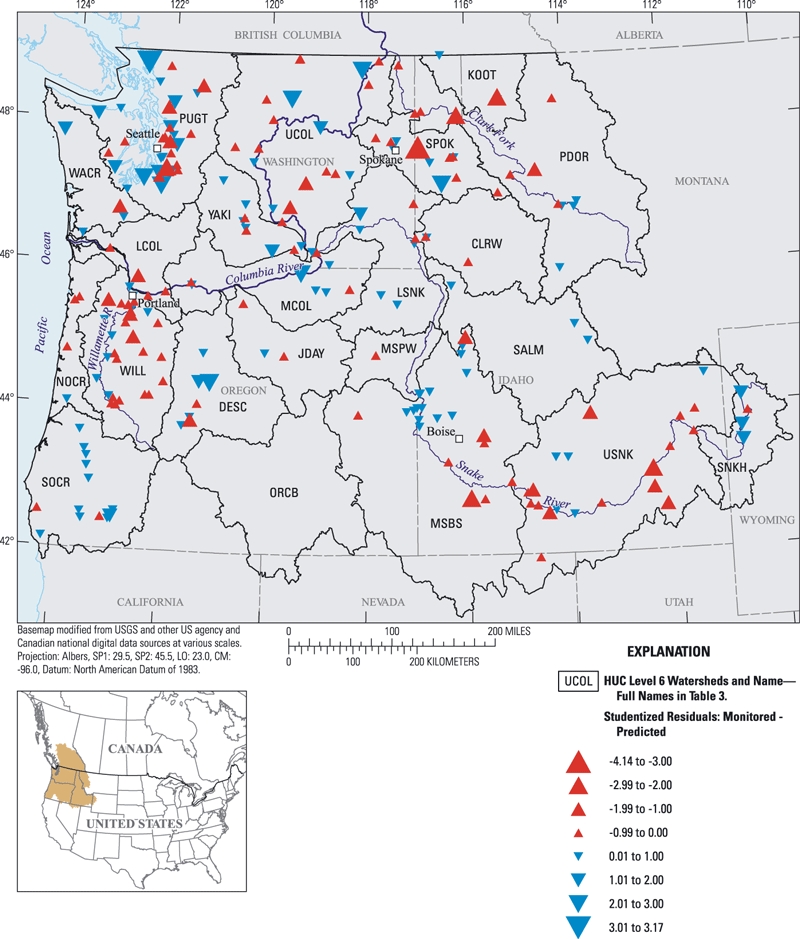
Spatial Distribution of Residual Stream Load for the Total Phosphorus SPARROW Model Developed for the United States Pacific Northwest.

Both the TN and TP models accounted for the spatial variability in monitored loads reasonably well (*R*^2^ = 0.892 for TN; *R*^2^ = 0.858 for TP; based on log-transformed data). Figures 2 and 3 show the studentized residuals, with the red triangles indicating overprediction and the blue triangles indicating underprediction. Table 3 lists the full names for the six-digit hydrologic unit code (HUC6) watersheds shown on Figures 2 and 3. The poorest model fit for TN was in the Upper Snake River (USNK) basin, generally because of overprediction; and the poorest model fit for TP was in the PUGT watershed, but with no bias toward under or overprediction. The best model fit for both TN and TP was in the Willamette River (WILL) basin. Neither model showed substantial collinearity among variables. The Supporting Information contains graphs comparing predicted and measured stream loads, predicted stream loads and model residuals, and predicted yields and model residuals.

**TABLE 3:** Full Names for Abbreviations Used for Six-Digit Hydrologic Unit Code (HUC6) Watersheds in the United States Pacific Northwest

Abbreviation	HUC6 Watershed
CLRW	Clearwater River
DESC	Deschutes River
JDAY	John Day River
KOOT	Kootenai River
LCOL	Lower Columbia River
LSNK	Lower Snake River
MCOL	Middle Columbia River
MSBS	Middle Snake - Boise Rivers
MSPW	Middle Snake - Powder Rivers
NOCR	Northern Oregon Coastal Rivers
ORCB	Oregon Closed Basins
PDOR	Pend Oreille River
PUGT	Puget Sound
SALM	Salmon River
SNKH	Snake River Headwaters
SOCR	Southern Oregon Coastal Rivers
SPOK	Spokane River
UCOL	Upper Columbia River
USNK	Upper Snake River
WACR	Washington Coastal Rivers
WILL	Willamette River
YAKI	Yakima River

The TN model identified eight source terms and two delivery terms that were statistically significant (Table 4). Although the term representing atmospheric deposition was not significant (*p* = 0.105), it was included in the model because it represented a background source of nitrogen for the entire region and was the only source of nitrogen in some watersheds. Stream attenuation was not identified as statistically significant in the TN model. The TP model identified five source terms and three delivery terms that were statistically significant (Table 4). We attempted to model phosphorus input from agricultural fertilizer use and manure from livestock production separately in the TP model (as we had done in the TN model), but only the latter was significant. We decided to combine these sources into one term in order to explicitly account for all the agricultural phosphorus input. Stream attenuation was a significant term in the TP model for reaches in which the estimated mean annual streamflow was <13.4 m^3^/s (meaning stream attenuation of TP was estimated in 80% of the stream reaches). Reservoir decay was not a significant term in either the TN model (coefficient = 2.21 m/year, *p* = 0.10) or the TP model (coefficient = 1.50 m/year, *p* = 0.15). The NLLS coefficient estimates for the TN and TP models were generally close in value to the mean coefficients estimates obtained from the bootstrap analysis, meaning that the uncertainty for the NLLS coefficients was low. The exception was the term representing the TP load exported from watersheds located primarily in Canada, which was an indication of high uncertainty for this coefficient.

**TABLE 4:** Model Statistics for the Total Nitrogen and Total Phosphorus SPARROW Models Developed for the United States Pacific Northwest

			90% Confidence Interval for the Model Coefficient				
						
Parameter	Model Coefficient Units	Model Coefficient	Lower	Upper	Standard Error of the Model Coefficient	Probability Level (*p*-value)	Nonparametric Bootstrap Estimate of Coefficient (mean)
**Total Nitrogen**							
*Sources*							
Primarily Canadian drainages^1^ (km^2^)	kg/km^2^/year	120	19	220	61	0.0256	107
Atmospheric deposition^2^ (kg/year)	Dimensionless	0.10	0.00	0.23	0.08	0.1050	0.11
Point sources^3^ (kg/year)	Dimensionless	1.60	0.24	2.96	0.82	0.0268	1.59
Farm fertilizer^4^ (kg/year)	Dimensionless	0.048	0.015	0.081	0.020	0.0080	0.047
Livestock manure^5^ (kg/year)	Dimensionless	0.113	0.034	0.192	0.048	0.0098	0.122
Developed land^6^ (km^2^)	kg/km^2^/year	941	495	1,387	270	0.0003	938
Forest land (west)^7^ (km^2^)	kg/km^2^/year	78	27	129	31	0.0060	79
Forest land (east)^8^ (km^2^)	kg/km^2^/year	115	73	156	25	<0.0001	113
Red alder trees^9^ (m^2^)	kg/m^2^/year	0.31	0.00	0.64	0.20	0.0597	0.31
*Land-to-water delivery*							
Effective precipitation^10^ (cm)	cm^−1^	1.07	0.69	1.46	0.23	<0.0001	1.46
Hydrologic landscape region 20^11^ (%)	Dimensionless	−1.19	−1.82	−0.57	0.38	0.0020	−0.57
*Aquatic loss*							
Instream	-	-	-	-	-	-	-
Reservoir	-	-	-	-	-	-	-
*Model diagnostics*							
MSE		0.410				*R*^2^ load	0.892
RMSE		0.640				*R*^2^ yield	0.759
Number of observations		178					
**Total Phosphorus**							
*Sources*							
Primarily Canadian drainages^1^ (km^2^)	kg/km^2^/year	3.80	−0.39	7.99	2.54	0.0679	0.09
Geologic material^12^ (ppm·km^2^)	Dimensionless	0.0155	0.0115	0.0194	0.0024	<0.0001	0.0151
Point sources^3^ (kg/year)	Dimensionless	1.03	0.21	1.86	0.50	0.0195	1.05
Farm fertilizer^4^ and livestock manure^5^ (kg/year)	Dimensionless	0.018	0.005	0.032	0.008	0.0122	0.017
Developed land^6^ (km^2^)	kg/km^2^/year	61.7	22.7	100.7	23.6	0.0048	53.0
*Land-to-water delivery*							
Effective precipitation^10^ (cm)	cm^−1^	1.41	1.15	1.66	0.15	<0.0001	1.42
Permeability^13^ (cm/hour)	hour/cm	−0.33	−0.49	−0.17	0.10	0.0008	−0.32
Hydrologic landscape region 20^11^ (%)	Dimensionless	−0.68	−1.13	−0.24	0.27	0.0122	−0.64
*Aquatic loss*							
Instream^14^	day^−1^	0.093	0.007	0.180	0.052	0.0383	0.083
Reservoir	-	-	-	-		-	-
*Model diagnostics*							
MSE		0.480				*R*^2^ load	0.858
RMSE		0.693				*R*^2^ yield	0.712
Number of observations		228					

Notes: SPARROW, SPAtially Referenced Regressions On Watershed attributes; MSE, mean squared error; RMSE, root mean squared error; -, not applicable.

1 Drainage area for watersheds with more than 50% located in Canada.

2 Annual wet deposition of inorganic nitrogen (ammonia and nitrate), 2002.

3 Surface-water discharges from permitted wastewater discharge, 2002.

4 Commercial fertilizer applied to agricultural land, 2002.

5 Manure produced by livestock animals, 2002.

6 Area of developed land, 2001.

7 Area of forest land, west side of Cascade Range, 2001.

8 Area of forest land, east side of Cascade Range, 2001.

9 Basal area of red alder trees, 2001.

10 Natural log of effective mean annual precipitation (local precipitation plus the depth of irrigation water).

11 Percentage of land that is hydrologic landscape region 20 (humid mountains with permeable soil and impermeable bedrock).

12 Phosphorus content of geologic material, mass ppm scaled by catchment area.

13 Natural log of mean soil permeability.

14 Phosphorus loss in stream with mean annual streamflow <13.4 m^3^/s.

The *p*-values for the source variables are based on a one-sided *t*-test; the *p*-values for the land-to-water delivery variables are based on a two-sided *t*-test.

### Prediction Results

#### Nutrient Yields

**FIGURE 4:**
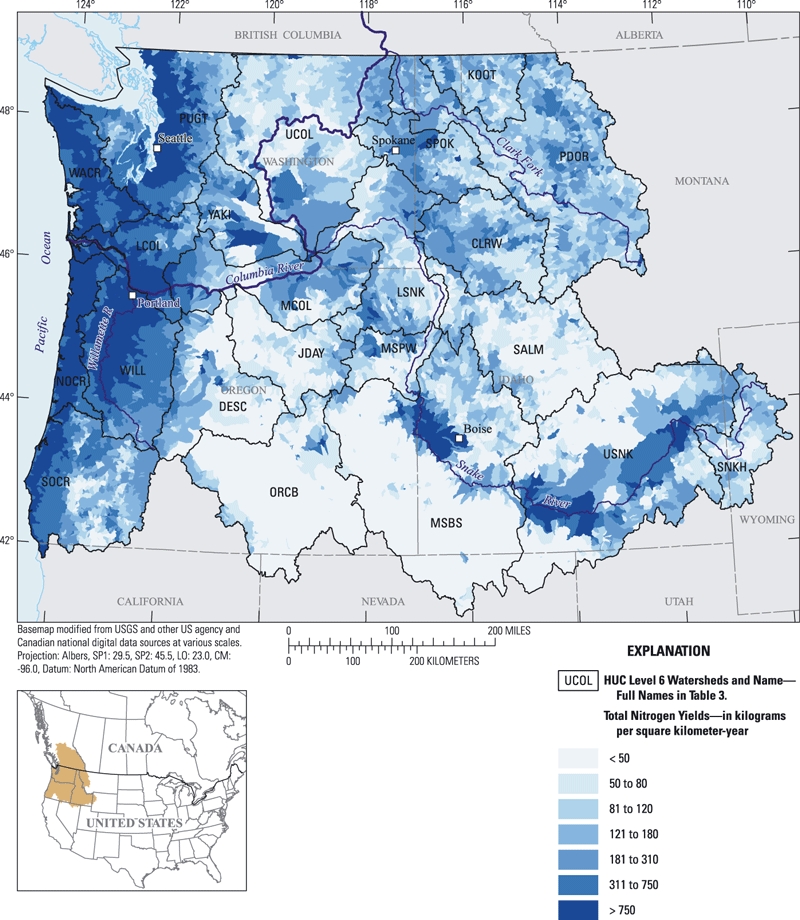
Incremental Total Nitrogen Yields in the United States Pacific Northwest (2002 conditions).

**FIGURE 5:**
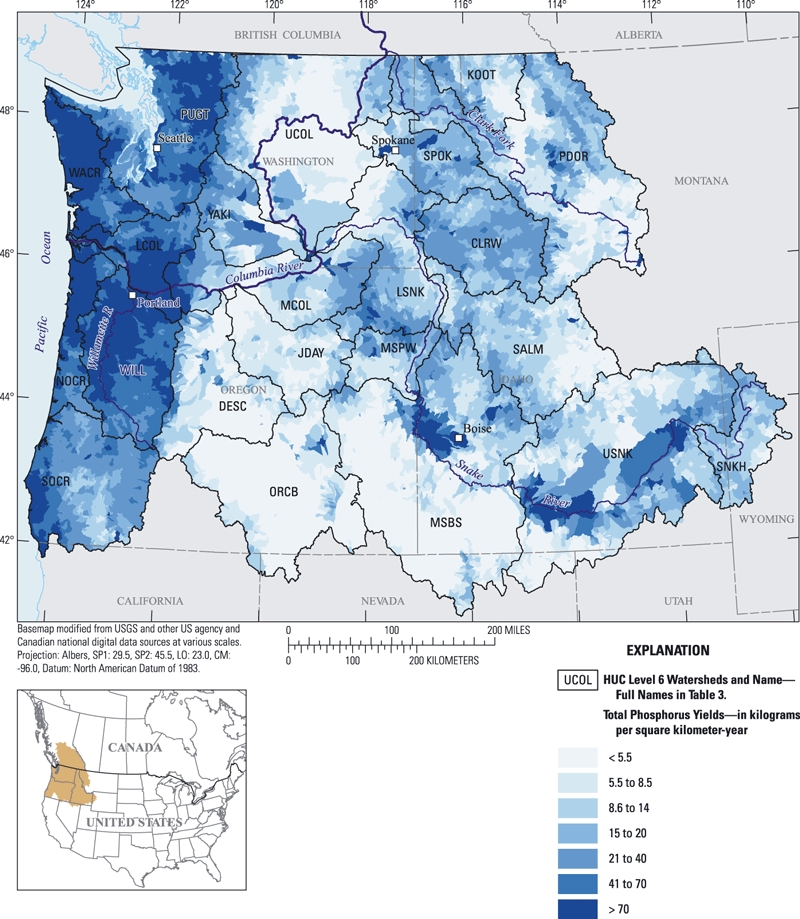
Incremental Total Phosphorus Yields in the United States Pacific Northwest (2002 conditions).

Table 5 summarizes the incremental TN and TP yields predicted for the PNW. The highest annual yields of TN and TP were predicted on the west side of the Cascades in the WILL and Lower Columbia River basins, the PUGT watershed, and the coastal drainages of Washington and Oregon (WACR, NOCR, and SOCR) (Figures 4 and 5). The lowest annual yields of TN and TP were predicted on the east side of the Cascades in the headwater basin for the Snake River, the John Day River and Deschutes River basins, and the Oregon closed basins. These patterns resulted from the large difference in precipitation between the two sides of the Cascades. Some high annual TN and TP yields, however, were predicted on the east side of the Cascades, in the Spokane River (SPOK), Yakima River (YAKI), USNK, and Middle Columbia River (MCOL) basins. These basins had large inputs of fertilizer and manure, and, in the case of the SPOK and Middle Snake River (MSPW) basins, large point-source inputs. The highest median annual TN and TP yields were in watersheds on the west side of the Cascades that were mostly (>75%) developed or agricultural land (Table 6). The lowest median annual TN and TP yields were in watersheds that were mostly range land and watersheds on the east side of the Cascades that were mostly forest land.

**TABLE 5:** Summary Statistics of Yields and Source Shares From Incremental Catchments in the United States Pacific Northwest (2002 conditions)

	Total Nitrogen							Total Phosphorus						
		
			Percentiles							Percentiles				
						
	Mean	SD	10th	25th	50th	75th	90th	Mean	SD	10th	25th	50th	75th	90th
Incremental yield (kg/km^2^/year)	475	5,910	17.5	53.9	121	301	846	72.2	188	5.82	8.60	15.7	39.1	76.80
Source shares (%)														
Point sources	1.0	8.3	0.0	0.0	0.0	0.0	0.0	3.9	10.6	0.0	0.0	0.1	0.0	0.0
Developed land	6.0	14.4	0.0	0.0	0.1	4.4	17.4	1.0	8.7	0.0	0.0	0.0	2.5	10.9
Primarily Canadian drainages	0.2	4.4	0.0	0.0	0.0	0.0	0.0	0.2	4.2	0.0	0.0	0.0	0.0	0.0
Forest land (east)	33.4	37.3	0.0	0.0	8.1	75.5	87.5	-	-	-	-	-	-	-
Forest land (west)	12.2	24.8	0.0	0.0	0.0	6.3	54.5	-	-	-	-	-	-	-
Farm fertilizer	8.3	17.0	0.0	0.0	0.1	6.3	32.5	-	-	-	-	-	-	-
Livestock manure	18.4	22.5	0.1	1.4	8.6	27.9	53.7	-	-	-	-	-	-	-
Farm fertilizer and livestockmanure	-	-	-	-	-	-	-	10.7	16.7	0.1	0.5	3.0	12.2	36.2
Geologic material	-	-	-	-	-	-	-	84.2	22.2	47.8	80.0	94.6	99.1	99.9
Atmospheric deposition	14.4	17.9	3.0	5.6	9.7	14.0	26.7	-	-	-	-	-		-
Red alder trees	6.3	15.2	0.0	0.0	0.0	0.5	28.7	-	-	-	-	-		-

Notes: SD, standard deviation; -, not applicable.

Incremental yields represent the load generated within an incremental watershed (the area that drains directly to a stream reach without passing through another stream reach) divided by the area of the incremental watershed. Incremental yield accounts for the effects of instream aquatic loss associated with one-half the reach time of travel (if applicable). Source shares represent the contribution from each source as a percentage of the incremental yield.

**TABLE 6:** SPARROW Predictions of Nutrient Yields From Major Land Cover Types in the United States Pacific Northwest (2002 conditions) Compared to Literature Estimates

					Nutrient Yields Obtained From the Literature (kg/ha/year)					
					
Median Yields Predicted by the PNW SPARROW Model (kg/ha/year)					Regional Values			National Values		
		
Dominant Land Cover Type	Location	Number	TN	TP	Nitrogen	Phosphorus	Data Sources	Nitrogen	Phosphorus	Data Sources
Agricultural land	East side^1^	180	3.78	0.37	6.33	0.55	a	14.0-22.0	0.90	l, m
	West side^2^	57	14.90	0.99	7.82	0.92	b			
Forest land	East side	1,901	1.27	0.13	0.14-0.68	nd	c, d	2.50-4.50	0.20	l, m
	West side	1,214	2.78	0.47	0.10-3.15	0.04-0.30	b, e, f			
	Coastal^3^	251	9.42	0.91	7.13-25.2	0.20-0.70	g, h, i, j, k	na	na	l, m
Developed land	East side	0	na	na	na	na	na	5.00-31.6	1.0	l, m
	West side	25	16.8	1.25	2.80	0.44	b			
Range land	East side	2,181	0.18	0.08	nd	nd	na	2.90-5.00	0.80	l, m
	West side	19	1.27	0.31						

Notes: SPARROW, SPAtially Referenced Regressions On Watershed attributes; PNW, Pacific Northwest; TN, total nitrogen; TP, total phosphorus; Number, number of incremental watersheds where at least 75% of the land consisted of a particular land cover type; na, not applicable; nd, no data available.

1 Watersheds located on the east side of the Cascade Range.

2 Watersheds located on the west side of the Cascade Range, but not in the Washington and northern Oregon coastal drainages.

3 Watersheds located in the Washington and northern Oregon coastal drainages.

Data sources for nutrient yields obtained from the literature: (a) Wise (2004) (unpublished data); (b) Embrey and Inkpen (1998); (c) Gravelle *et al.* (2009); (d) Schaefer and Hollibaugh (2009); (e) Martin and Harr (1988); (f) Vanderbilt *et al.* (2003); (g) Brown *et al.* (1973); (h) Edmonds *et al.* (1995); (i) Compton *et al.* (2003); (j) Brown and Ozretich (2009); (k) Schaefer and Hollibaugh (2009); (l) Alexander *et al.* (2001); (m) Beaulac and Reckhow (1982).

#### Nutrient Enrichment

**FIGURE 6:**
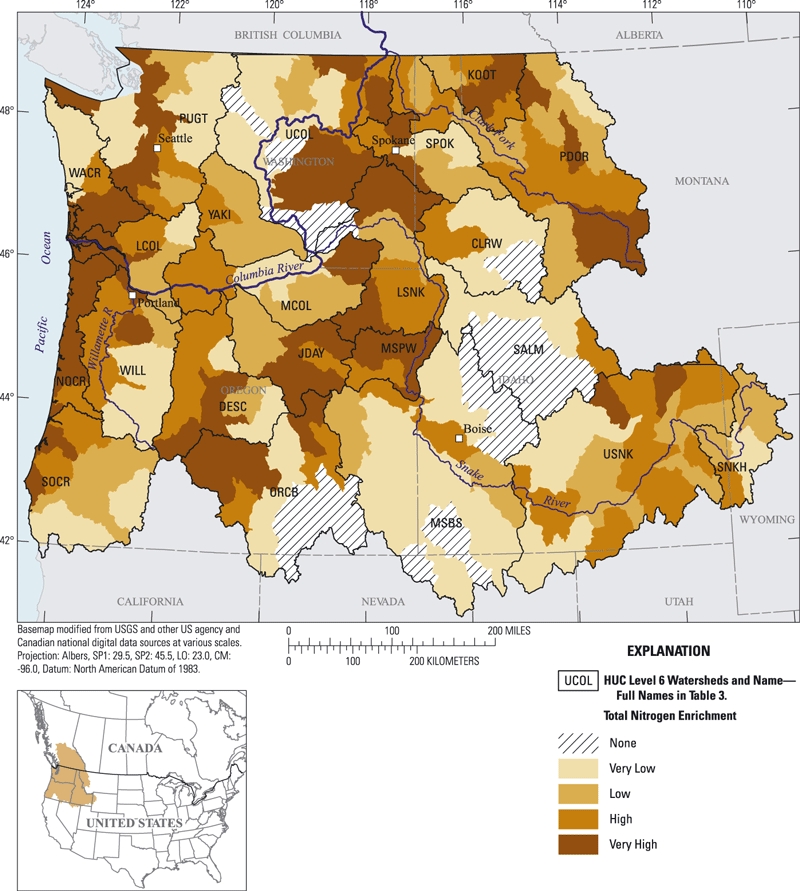
Estimated Total Nitrogen Enrichment for the United States Pacific Northwest (2002 conditions). Results are shown for each HUC8 watershed.

**FIGURE 7:**
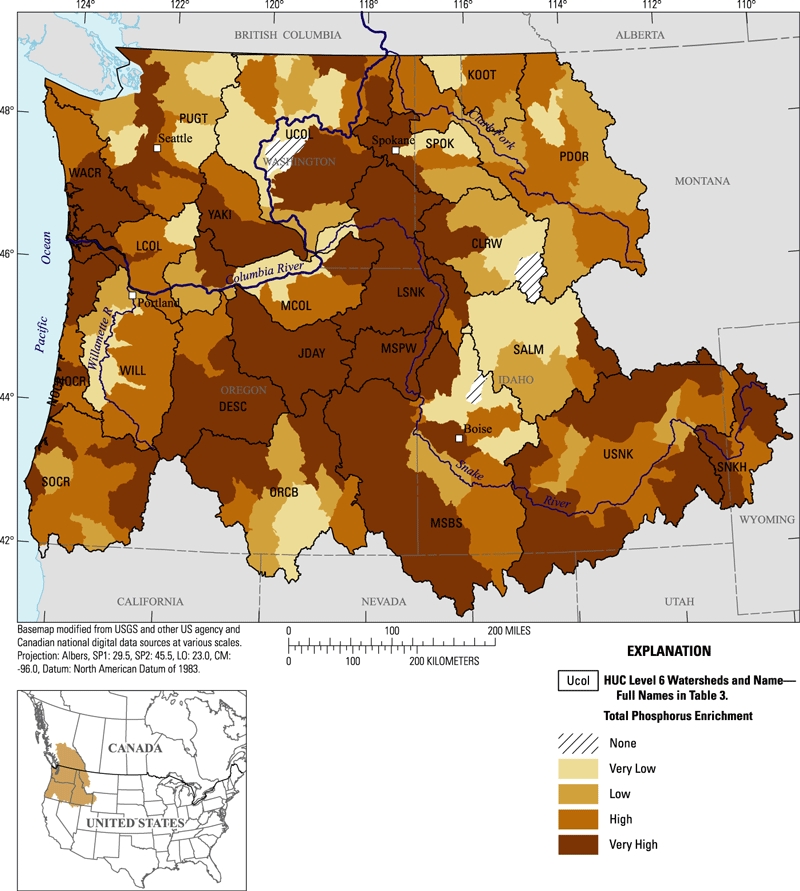
Estimated Total Phosphorus Enrichment for the United States Pacific Northwest (2002 conditions). Results are shown for each HUC8 watershed.

Our results comparing mean annual concentrations estimated by SPARROW to the recommended USEPA nutrient criteria (Table 2) are shown on Figures 6 and 7. High nutrient enrichment was estimated throughout the PNW, with the highest TN enrichment estimated in watersheds along the northern Oregon coast (NOCR) and the highest TP enrichment estimated in the MSPW basin. The lowest nutrient enrichment was estimated in the Salmon River (SALM), Clearwater River (CLRW), and Upper Columbia River (UCOL) basins (many of the HUC8 watersheds in these basins had very low or no estimated nutrient enrichment). TP enrichment was generally higher and more widespread than TN enrichment. Very high TP enrichment was estimated in 68 of the 219 HUC8 watersheds compared to 45 watersheds for TN, and very low or no TP enrichment was estimated in only 36 HUC8 watersheds compared to 85 watersheds for TN. TP enrichment was greater than TN enrichment in 67% of the HUC8 watersheds. The Supporting Information contains the nutrient enrichment results for each HUC8 watershed in the PNW.

#### Nutrient Sources

**FIGURE 8:**
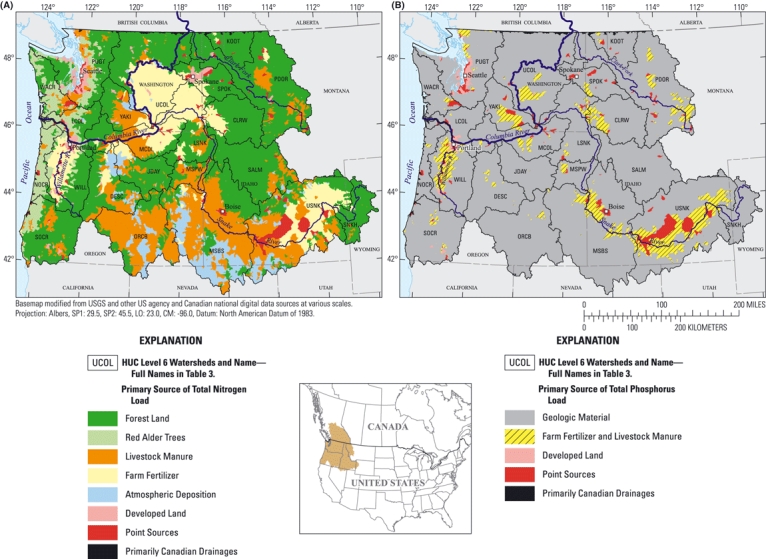
Primary Sources of Total Nitrogen (A) and Total Phosphorus (B) Exported From Incremental Watersheds in the United States Pacific Northwest (2002 conditions).

**FIGURE 9:**
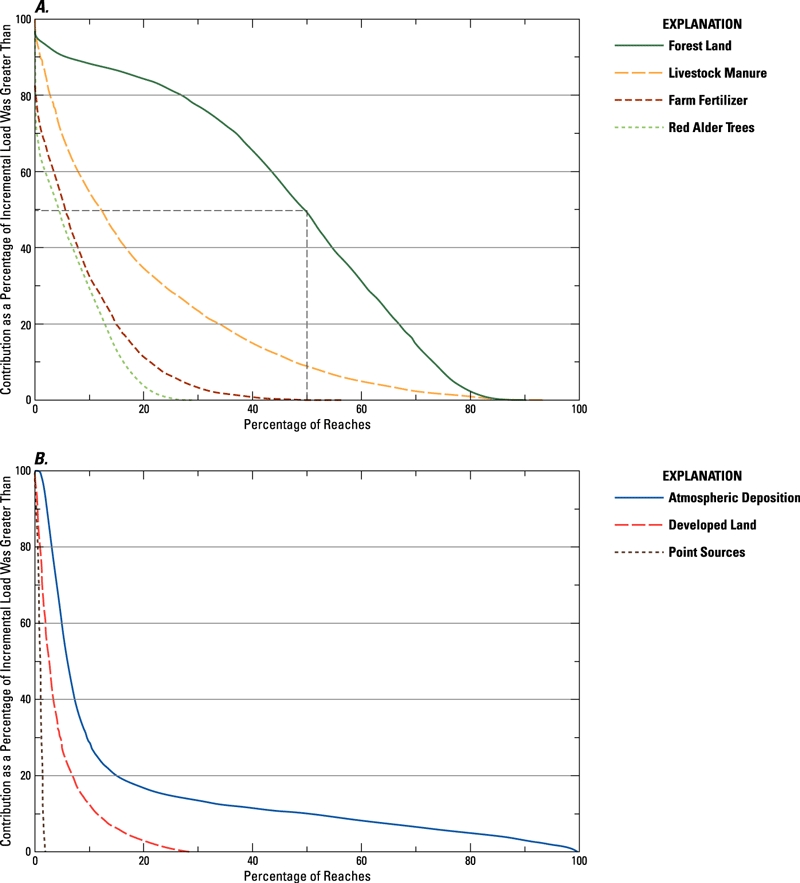
Share of Total Nitrogen Stream Exported From Incremental Watersheds in the United States Pacific Northwest Attributed to (A) Forest Land, Animal Manure, Farm Fertilizer, and Red Alder Trees, and (B) Atmospheric Deposition, Developed Land, and Point Sources (2002 conditions).

**FIGURE 10:**
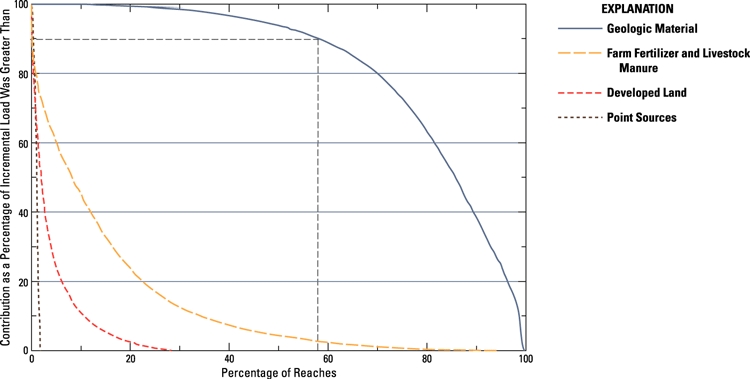
Share of Total Phosphorus Stream Exported From Incremental Watersheds in the United States Pacific Northwest Attributed to Farm Fertilizer and Animal Manure, Developed Land, Geologic Material, and Point Sources (2002 conditions).

Forest land was the largest contributor to the incremental TN stream load (the stream load generated in and exported from each incremental watershed), and geologic material was the largest contributor to the incremental TP stream load (Table 5 and Figure 8). Other nutrient sources besides forest land and geologic material, however, dominated in selected areas of the region. Animal manure and agricultural fertilizer were the largest contributors to incremental nutrient stream load in much of the WILL, YAKI, UCOL, MCOL, MSPW, MSBS, and USNK basins; alder trees were the largest contributor to incremental TN stream load in much of the WACR, NCOR, and SOCR basins; developed land was the largest contributor to incremental TN and TP stream load in the urban areas of the PUGT basin and around Portland and Spokane; and atmospheric deposition was the largest contributor to incremental TN stream load along the southern edge of the PNW where there was very little input from other nitrogen sources. Figures 9 and 10 provide more detail about the relative contribution from different nutrient sources to the incremental nutrient stream loads. The *x*-axis on each graph shows the percentage of reaches where the contribution from each source was greater than the percentage indicated on the y axis. Forest land was the source of more than 50% of the incremental TN stream load in 50% of the reaches and geologic material was the source of more than 90% of the incremental TP stream load in 58% of the reaches (as indicated by the lines on the plots). The shapes of the individual graphs can also be used to compare the relative importance of different sources. For example, although both atmospheric deposition and forest land contributed some of the TN stream load generated within almost all of the incremental watersheds, atmospheric deposition was responsible for more than 50% of the stream load in very few watersheds compared to forest land (<10 *vs.* 50%).

While the results presented in Figure 8 are useful for showing where different nutrient sources were important, they do not show the impact that large, locally important sources had on downstream receiving waters. SPARROW can estimate these impacts because of the spatially explicit nature of the model. Tables 7 and 8, respectively, show the predicted TN and TP stream loads discharged from 21 of the 22 six-digit hydrologic unit code (HUC6) watersheds in the PNW (one HUC6 watershed drains internally) and the relative contribution to those stream loads estimated from all upstream sources (some of which are in upstream HUC6 watersheds). The combined input from agriculture, point sources, and developed land was responsible for much of the nutrient stream load discharged from the HUC6 watersheds. In 11 HUC6 watersheds these sources in aggregate contributed most of the TN stream load, and in 9 HUC6 watersheds they contributed most of the TP stream load. Point sources are an example of an important local nutrient source that also affected downstream receiving waters. Although only 2% of the incremental watersheds in the PNW received nutrient input from point sources, point sources were the largest source of TN stream load discharged from five HUC6 watersheds and the largest source of TP stream load discharged from three HUC6 watersheds.

**TABLE 7:** Estimated Stream Loads and Source Shares of Total Nitrogen Discharged From Six-Digit Hydrologic Unit Code (HUC6) Watersheds in the United States Pacific Northwest (2002 conditions)

Six-Digit Hydrologic Unit Code (HUC6) Watershed	Estimated Load (metric ton/year)	Share From Primarily Canadian Drainages (%)	Share From Forest Land (%)	Share From Atmospheric Deposition (%)	Share From Farm Fertilizer (%)	Share From Livestock Manure (%)	Share From Point Sources (%)	Share From Developed Land (%)	Share From Alder Trees (%)
Kootenai River (170101)	2,115	66.3	24.7	3.7	2.0	1.7	0.8	0.9	0.0
Pend Oreille River (170102)	2,166	2.0	55.4	9.0	4.9	13.9	11.5	3.3	0.0
Spokane River (170103)	5,452	0.0	28.0	4.9	8.5	5.8	40.8	11.9	0.0
Yakima River (170300)	3,205	0.0	12.5	5.0	26.1	43.5	5.2	7.7	0.0
Snake River Headwaters (170401)	1,326	0.0	65.3	15.3	6.0	11.9	0.8	0.7	0.0
Upper Snake River (170402)	7,747	0.0	1.5	1.8	19.4	36.9	38.9	1.5	0.0
Middle Snake-Boise Rivers (170501)	19,145	0.0	4.2	2.0	20.7	40.3	30.6	2.3	0.0
Middle Snake-Powder Rivers (170502)	23,324	0.0	6.7	2.2	19.8	40.8	28.3	2.2	0.0
Salmon River (170602)	2,741	0.0	72.6	9.3	2.2	12.9	2.3	0.7	0.0
Clearwater River (170603)	4,218	0.0	57.0	8.3	16.4	5.9	11.3	1.1	0.0
Lower Snake River (170601)	21,314	0.0	22.0	4.1	20.2	31.0	20.7	2.0	0.0
Upper Columbia River (170200)	82,592	1.8	21.7	4.8	21.5	26.9	19.5	3.9	0.0
John Day River (170702)	851	0.0	61.2	8.3	7.2	23.0	0.0	0.3	0.0
Deschutes River (170703)	1,945	0.0	44.7	9.1	20.7	21.2	0.0	4.3	0.0
Middle Columbia River (170701)	91,490	1.5	24.0	5.2	22.1	25.5	17.3	4.1	0.3
Willamette River (170900)	25,243	0.0	12.9	2.8	27.2	10.9	30.0	9.3	6.8
Lower Columbia River (170800)	97,507	0.7	18.9	4.4	19.7	17.2	26.0	6.5	6.4
Puget Sound (171100)	30,685	0.6	13.9	4.0	2.9	10.9	36.1	16.8	14.8
Washington Coastal Rivers (171001)	13,932	0.0	34.3	6.1	2.5	9.5	5.1	5.5	37.0
Northern Oregon Coastal Rivers (171002)	13,719	0.0	23.5	4.6	1.5	11.0	1.2	2.7	55.5
Southern Oregon Coastal Rivers (171003)	12,452	0.0	39.3	4.1	4.2	14.1	8.4	4.7	25.3
Oregon Closed Basins (171200)	na	na	na	na	na	na	na	na	na

Notes: Source shares represent contributions from all upstream sources; na, not applicable because there is no surface water outlet from the watershed.

**TABLE 8:** Estimated Stream Loads and Source Shares of Total Phosphorus Discharged From Six-Digit Hydrologic Unit Code (HUC6) Watersheds in the United States Pacific Northwest (2002 conditions)

Six-Digit Hydrologic Unit Code (HUC6) Watershed	Estimated Load (metric ton/year)	Share From Primarily Canadian Drainages (%)	Share From Geologic Material (%)	Share From Farm Fertilizer and Livestock Manure (%)	Share From Point Sources (%)	Share From Developed Land (%)
Kootenai River (170101)	174	42.1	49.9	4.9	1.9	1.2
Pend Oreille River (170102)	235	0.8	75.4	11.0	10.5	2.3
Spokane River (170103)	186	0.0	45.9	5.9	42.6	5.6
Yakima River (170300)	670	0.0	52.4	23.1	18.3	6.2
Snake River Headwaters (170401)	299	0.0	94.1	5.5	0.1	0.3
Upper Snake River (170402)	718	0.0	13.0	22.7	63.5	0.7
Middle Snake-Boise Rivers (170501)	1,242	0.0	25.3	26.3	47.2	1.2
Middle Snake-Powder Rivers (170502)	1,785	0.0	31.7	25.5	41.7	1.2
Salmon River (170602)	341	0.0	93.2	4.3	2.0	0.5
Clearwater River (170603)	375	0.0	75.2	14.0	10.3	0.5
Lower Snake River (170601)	1,630	0.0	51.1	20.4	27.5	1.0
Upper Columbia River (170200)	3,677	0.9	52.5	18.6	26.1	2.0
John Day River (170702)	107	0.0	91.9	7.9	0.0	0.3
Deschutes River (170703)	366	0.0	84.6	13.5	0.0	1.9
Middle Columbia River (170701)	5,024	0.7	55.4	16.9	24.7	2.4
Willamette River (170900)	2,438	0.0	45.2	19.7	27.5	7.5
Lower Columbia River (170800)	10,272	0.3	50.0	14.5	30.5	4.6
Puget Sound (171100)	3,283	0.2	44.9	5.0	39.9	10.0
Washington Coastal Rivers (171001)	1,563	0.0	78.9	4.6	12.2	4.3
Northern Oregon Coastal Rivers (171002)	880	0.0	86.9	8.2	1.4	3.4
Southern Oregon Coastal Rivers (171003)	1,470	0.0	80.3	9.0	7.4	3.4
Oregon Closed Basins (171200)	na	na	na	na	na	na

Notes: Source shares represent contributions from all upstream sources; na, not applicable because there is no surface water outlet from the watershed.

Our SPARROW models did not include separate regression coefficients for each of the different types of point sources (e.g., sewage treatment facilities, animal aquaculture and fish hatcheries, and pulp and paper mills). We estimated the contribution to stream load from different types of point sources in each HUC6 watershed, however, by calculating the percentage of the total input of nitrogen and phosphorus in each watershed from each type and assuming that the point-source contribution at each HUC6 outlet reflected these percentages. Sewage treatment facilities contributed most of the point-source load discharged from the HUC6 watersheds in the PNW (Tables 9 and 10). The exception was the USNK basin, where aquaculture facilities and fish hatcheries contributed more than 50% of the point-source load. Most of these point sources, however, used water from the Snake River and its tributaries, meaning that most of the nutrient load discharged from these facilities was simply diverted stream load. Sampling of influent and effluent at the largest USNK aquaculture facilities and fish hatcheries (>1,000 kg/year) indicated that, on average, about 50% of the discharged TP load was due to influent load (sampling data were obtained from Molly Maupin of the USGS, written communication August 26, 2009; there were not enough TN samples to estimate the contribution of influent load to discharged TN load). The input from the USNK aquaculture facilities and fish hatcheries was also an important contributor to stream load in the Lower Snake River and, to a lesser degree, in the Columbia River below its confluence with the Snake River.

**TABLE 9:** Summary of Point-Source Total Nitrogen Input to Selected Six-Digit Hydrologic Unit Code (HUC6) Watersheds in the United States Pacific Northwest (2002 conditions)

		Contribution to Total Nitrogen Point-Source Loading (%)				
		
Six-Digit Hydrologic Unit Code Watershed	Point-Source Contribution to Discharged Total Nitrogen Load (%)	Sewage Treatment Facilities	Animal Aquaculture and Fish Hatcheries	Pulp and Paper Mills	Food Processing	Other
Spokane River (170103)	42.0	99.0	0.0	1.0	0.0	0.0
Upper Snake River (170402)	38.7	35.1	52.2	0.0	12.7	0.0
Middle Snake-Boise Rivers (170501)	30.4	53.4	35.6	0.0	10.9	0.0
Middle Snake-Powder Rivers (170502)	28.2	54.3	35.0	0.0	10.8	0.0
Lower Snake River (170601)	20.9	55.0	31.8	3.5	9.8	0.0
Upper Columbia River (170200)	20.0	68.0	21.7	3.5	6.7	0.1
Willamette River (170900)	30.1	96.8	0.0	3.1	0.0	0.1
Lower Columbia River (170800)	26.3	83.0	7.4	5.6	2.3	1.7
Puget Sound (171100)	37.4	95.7	0.2	3.4	0.0	0.6

Notes: Values indicate the percentage of the total point-source loading from each category, and reflect the total upstream area draining to the outlet of each watershed. Results are only shown for watersheds where point sources contributed at least 20% of the total nitrogen load discharged from the watershed.

**TABLE 10:** Summary of Point-Source Total Phosphorus Input to Selected Six-Digit Hydrologic Unit Code (HUC6) Watersheds in the United States Pacific Northwest (2002 conditions)

		Contribution to Total Phosphorus Point-Source Loading (%)				
		
Six-Digit Hydrologic Unit Code Watershed	Point-Source Contribution to Discharged Total Phosphorus Load (%)	Sewage Treatment Facilities	Animal Aquaculture and Fish Hatcheries	Pulp and Paper Mills	Food Processing	Other
Spokane River (170103)	42.6	96.0	0.0	3.7	0.0	0.3
Upper Snake River (170402)	63.5	42.6	54.0	0.0	3.4	0.0
Middle Snake-Boise Rivers (170501)	47.2	61.2	35.8	0.0	3.0	0.0
Middle Snake-Powder Rivers (170502)	41.7	62.3	34.7	0.0	2.9	0.0
Lower Snake River (170601)	27.5	62.8	30.7	3.9	2.6	0.0
Upper Columbia River (170200)	26.1	71.3	21.1	5.2	1.8	0.7
Middle Columbia River (170701)	24.7	60.7	16.5	20.8	1.4	0.5
Willamette River (170900)	27.5	83.7	0.0	16.1	0.0	0.2
Lower Columbia River (170800)	30.5	61.2	5.6	30.2	0.5	2.5
Puget Sound (171100)	39.9	72.3	0.1	27.5	0.0	0.1

Notes: Values indicate the percentage of the total point-source loading from each category, and reflect the total upstream area draining to the outlet of each watershed. Results are only shown for watersheds where point sources contributed at least 20% of the total phosphorus load discharged from the watershed.

## Discussion

### Model Calibration

The source coefficients used in our SPARROW models represented the export of nutrients (e.g., kg N/km^2^/year) from a hypothetical incremental watershed where the values for all the delivery terms included in the model were equal to the average values for all the incremental watersheds. As a result, it is difficult to interpret them in relation to nutrient export coefficients obtained from the literature. However, they should still be consistent with our understanding of the sources that were modeled. For example, because permitted wastewater discharges release nutrients directly to streams rather than on land, the expected coefficient for the point-source terms in our SPARROW models was 1.0. The coefficient of 1.59 for the point-source term in our TN model, rather than a value closer to 1.0, could be due to underestimation of TN discharge from these sources. As TN and the nitrogen species needed to calculate TN are often not measured in wastewater effluent, a regional average based on a limited number of TN measurements was used to estimate the TN discharge from many of the point sources in the PNW (Maupin and Ivahnenko, this issue). To the extent that this regional average was less than the actual concentration of TN at facilities where data were lacking, the TN discharged from these facilities would have been underestimated.

Other examples are the source terms that are expressed on a mass per mass basis. The coefficient of 0.10 for wet deposition of inorganic nitrogen appears reasonable given the capacity of forest ecosystems to assimilate and retain added nitrogen (Aber, 1992). The farm fertilizer coefficients (<0.05 for both nitrogen and phosphorus) appear reasonable if we assume that fertilizer application rates were generally close to crop requirements (our models did not explicitly account for nutrient loss through fertilizer uptake and crop harvesting). The higher coefficient for nitrogen from livestock manure compared to nitrogen from farm fertilizer also appears reasonable given that the nitrogen in animal waste is primarily in the organic form (which becomes available to plants only after microbial mineralization) and the nitrogen in farm fertilizer is in the inorganic form (which can be taken up directly by crops).

The positive or negative values for the land-to-water delivery coefficients, rather than their magnitudes, provide information on how they influenced the model. The positive coefficient for effective precipitation in both the TN and TP models was expected, because areas with high precipitation experience more runoff than areas with low precipitation; and the extremely high significance of this delivery term was due to the very large precipitation gradient across the PNW. The hydrologic landscape region that was a significant delivery term in both models (humid mountains with permeable soil and impermeable bedrock; hydrologic landscape region 20) represented high elevation watersheds (generally above 1,000 m). These watersheds were mostly located in the coastal mountains in southwestern Oregon, the Cascade Range in Washington, and the Rocky Mountains in Idaho. The negative coefficient for this hydrologic landscape region was consistent with our understanding of the dominant hydrologic processes in these areas. An elevation of 1,000 m generally is the transition point between rain and snow during the wet season in the PNW (November-March). Therefore, most precipitation above 1,000 m falls as snow and is released slowly during the spring and early summer melt, with much of it infiltrating into the soil rather than running off directly to streams. This increased infiltration could result in greater biological processing of nitrogen and phosphorus in the soil and less delivery to streams than in other watersheds. The negative coefficient for mean permeability in the TP model was consistent with the presumed effect that this soil property has on the delivery of phosphorus to streams. Areas with high permeability soils should experience greater infiltration and less surface runoff than areas with low permeability soils. As a result, there would be less erosion and delivery of soil to streams in areas with high permeability soils than in areas with low permeability soils and, because phosphorus tends to attach to soil particles, less delivery of phosphorus to streams.

Stream attenuation is usually identified as statistically significant in SPARROW nutrient models (e.g., Smith *et al.*, 1997; Preston and Brakebill, 1999; Moore *et al.*, 2004; Hoos and McMahon, 2009). While this was true for our TP model, it was not the case for the TN model (*p* > 0.25). This result does not mean that nitrogen attenuation does not occur in PNW streams; rather, it could have been due to an interaction between the stream attenuation term and the source terms representing nitrogen export from forest land. Stream attenuation is greater in smaller streams compared to larger streams (Alexander *et al.*, 2000; Peterson *et al.*, 2001), and most of the smallest stream reaches in the PNW (i.e., the first and second order streams) are located in watersheds that generally included a high percentage of forest land (>50%). The coefficients for the forest land terms may account for nitrogen attenuation in lower order reaches, thus eliminating the need to account for these processes through an attenuation term applied to small streams. The lack of a stream attenuation term could also have resulted, however, from the short residence times (due to high slopes), cool water temperatures, low organic material, rocky substrates, and low solar energy (due to riparian canopy cover) found in forested streams.

The SPARROW models we developed for the PNW were configured differently than those that were developed for the U.S. using the same stream network (Smith *et al.*, 1997). While some of the source coefficients we used in our models (atmospheric deposition, farm fertilizer, livestock manure, and point sources) were the same ones used in the national SPARROW models, others were not. Where the national models used nonagricultural land to represent runoff from urban, forest, and range land, we included urban (developed) and forest land explicitly in our models. We also included two source terms that were not included in the national models: fixation of nitrogen by red alder trees and weathering of geologic phosphorus. We believe that these refinements and the fact that our models were calibrated to the PNW resulted in more accurate predictions for our region compared to those from the national models. One way to evaluate spatially overlapping SPARROW models is to compare how well they predicted the monitored loads at the same water-quality stations. There were 14 calibration stations that were used in our PNW models as well as in national TN and TP models. The differences between the predicted and monitored loads at these stations was generally less for our models compared to the national models, based on the average absolute value for the log of the residuals (0.27 *vs.* 0.51 for TN; 0.36 *vs.* 0.46 for TP).

### Model Predictions

The nutrient yields we predicted for different land cover types were close to or within the range of estimated values for the PNW and the nation and showed the same spatial patterns, that is, the highest nutrient export was from agricultural and urban land and the lowest was from forest and range land (Table 6; the Supporting Information provides details for each of the regional and national studies). The exception to this pattern were the TN yields we predicted for forest land in the WACR and NOCR watersheds, which were much higher than the yields estimated for forest land across the nation but close to or within the range of values that have been measured within these watersheds. The high yields we predicted for the forested coastal watersheds (they were almost as high as those for west side agricultural and developed land) was likely due to the combination of relatively high precipitation and the inclusion of red alder trees as a source term in the model. On average, red alder trees contributed almost one-half the TN load discharged from the Washington and northern Oregon coastal drainages (Table 8).

The differences between our nutrient yields and the regional and national nutrient yields might be attributed to several factors. Our predictions reflected the conditions across many watersheds in our region, whereas the nutrient yields measured in the PNW by other researchers were typically based on studies of a small number of watersheds that met certain criteria (e.g., undisturbed forested watersheds). The nutrient yields generalized for the nation, on the other hand, reflected a broad range of watersheds that were likely very different from those typically found in the PNW (e.g., those with different crop types and range land with different grazing patterns). Finally, other watershed properties besides the type of land cover can affect nutrient export. These include nutrient loading rates, climate, landscape characteristics, and stream properties (Beaulac and Reckhow, 1982).

Our SPARROW models included two terms that represented background nutrient sources that were spatially continuous across the region. These two sources, however, differed substantially in their contribution to nutrient loads. On average, weathering of geologic phosphorus contributed 84.2% of the TP load exported from the incremental watersheds and 60.8% of the TP load exported from the HUC6 watersheds, whereas wet deposition of inorganic nitrogen contributed 14.4% of the TN load exported from the incremental watersheds and 5.7% of the TN load exported from the HUC6 watersheds. We used our model results to calculate the median TP yield due solely to weathering of geologic phosphorus for the 13 self-contained HUC6 watersheds in the PNW (i.e., those without HUC6 watersheds immediately upstream). While no comparable data for phosphorus was available for the PNW, our value of 0.19 kg/ha/year was close to the median value of 0.11 kg/ha/year calculated from available estimates of phosphorus release rates due to weathering for 12 river basins around the world (Gardner, 1990). The only data we found for the export of atmospherically derived nitrogen from PNW watersheds was estimated for the PUGT basin using the nationally calibrated SPARROW model (Alexander *et al.*, 2001). The atmospherically derived TN yield predicted by our model (0.35 kg/ha/year) was less than the value predicted by the national model (0.8 kg/ha/year), and the contribution from atmospherically derived nitrogen to the TN load discharged to the PUGT was less using our model (4.0%) compared to the national model (12.0%). The differences between the two models are not surprising given that the national model needed to account for the variations in atmospheric nitrogen deposition across the U.S. (NADP, 2011).

### Model Uncertainty

Successful calibration of the SPARROW model requires appropriate selection and accurate quantification of monitored loads, sources, land-to-water delivery factors, and instream attenuation processes. Notwithstanding our use of the best available information as input to the models described here, we recognize that certain nutrient sources and ecosystem processes are not explicitly accounted for in the model terms. In some cases, we did not have enough information to quantify an important source or process. In other cases, we were able to quantify a variable, but the variable was not found to be statistically significant because it was strongly correlated with another significant variable (e.g., septic tank density was negatively related to the area of developed land). During calibration, these unspecified sources and processes were indirectly accounted for in the estimated values of more broadly defined coefficients (those representing point sources, for example). Also, uncertainty in the estimated model coefficients might have been due to the finite sample sizes of the models and measurement errors associated with model input data. Such uncertainty might be reduced through the collection of additional water-quality data (especially in currently unmonitored areas) and refinements in the estimates of landscape attributes (especially nutrient source loadings).

Although we were not able to quantify the errors in the nutrient source loadings, we have some information about where they might have occurred when the data were compiled. The point-source loads, for example, were often based on a regionally averaged TN or TP concentration rather than on measured values. In addition, the point-source data consisted almost entirely of major sources (those permitted at more than 1 million gallons per day), and did not include fish hatcheries and aquaculture facilities located outside of Idaho or wastewater facilities that discharge to land rather than directly to streams. Another example was the parameterization of phosphorus in geologic material, which was based on streambed sediment sampling results that were extrapolated across the PNW rather than on soil and rock analyses.

The national geospatial dataset of nutrient loadings from livestock that we used in our models might have underestimated these loadings in some areas of the PNW and overestimated them in other areas. The county-level nutrient loadings from confined livestock animals were equally distributed to row crops, pasture, and hay fields within each county without considering the proximity of that farmland to animal feeding operations, which typically apply animal wastes to nearby fields that are used to grow crops for animal feed (Personal conversation with Joseph Harrison, Washington State University Extension Service, December 21, 2010). Also, animal waste from poultry farms was included in the county-level nutrient loadings from confined livestock animals even though most of that waste is shipped out of the counties in which the poultry farms are located (Personal conversation with Julie Walker, Washington State Department of Agriculture, January 11, 2011). The county-level nutrient loadings from unconfined livestock animals were equally distributed to row crops, pasture and hay fields, and grassland within each county, even though animal grazing on row crops is very uncommon in the PNW (USDA, 2009) and PNW rangeland also includes forest and shrub land (Personal conversation with David Ganskopp, USDA Agricultural Research Service, January 13, 2010). Also, the distribution of nutrient loadings from unconfined livestock animals did not consider the suitability of the land for grazing (e.g., vegetation abundance, access to water, and slope), and the methods used to distribute county-level nutrient loadings from livestock animals and fertilizer to farm land did not account for the nutrient requirements of individual crops.

The estimates of wet inorganic nitrogen deposition that we used in our models were based on interpolated data collected at stations (21 in the PNW) generally located away from developed and agricultural land (NADP, 2011). Therefore, these data most likely did not fully account for localized impacts from urban areas (e.g., emissions from automobiles and stationary combustion sources) or agricultural areas (e.g., volatilized ammonia from confined livestock). Also, the estimates of atmospheric nitrogen deposition did not include organic nitrogen, which may constitute a substantive input of atmospheric nitrogen to terrestrial and aquatic ecosystems (Neff *et al.*, 2002), nor did they account for dry or fog deposition, which might be important mechanisms for atmospheric deposition in areas of the PNW with low precipitation.

Our estimates of nutrient enrichment were based on the assumption that SPARROW can accurately predict mean annual TN and TP concentrations. We can test this assumption by comparing the SPARROW-predicted mean annual concentrations to the mean annual concentrations at our calibration stations. Both sets of data represented flow-weighted concentrations (i.e., the cumulative load for the period of record divided by the cumulative streamflow) and were detrended to the 2002 base year. The SPARROW-predicted mean annual TN and TP concentrations agreed well with the mean annual concentrations obtained from Fluxmaster (*R*^2^ = 0.65 for TN; *R*^2^ = 0.69 for TP; based on log-transformed data). Most of the difference between the two sets of data was due to SPARROW model error, which we accounted for in our estimates of nutrient enrichment (see Equation 1).

### Water-Quality Management

The SPARROW models we developed for the PNW, as well as future SPARROW models (whether calibrated for the entire PNW or just parts of the region) could be used as tools for designing water-quality assessment programs and making water-quality management decisions. Water-quality models play a central role in the Total Maximum Daily Load (TMDL) process by providing a means for predicting water-quality conditions and assessing the effectiveness of water-quality improvement strategies (National Research Council, 2001).

One example of how SPARROW could be used as a tool for informing water-quality assessments is by comparing the SPARROW-based estimates of nutrient enrichment to the prevalence of state 303(d) listings. We have used TP enrichment as an example because it was generally higher and more widespread than TN enrichment. Sixty-seven percent of the HUC8 watersheds in the PNW had at least one ERF1-2 stream reach on a state 303(d) list because of water-quality impairment related to nutrient enrichment, and these watersheds were evenly distributed throughout the region (the Supporting Information has a map showing the prevalence of 303(d) listings in PNW HUC8 watersheds). There were eight HUC8 watersheds where TP enrichment was estimated by SPARROW to be high or very high and most of the stream length had been placed on a 303(d) list, indicating that existing nutrient assessment programs adequately identified nutrient-enriched stream reaches in those watersheds (Table 11). There were 40 HUC8 watersheds, however, where TP enrichment was estimated to be high or very high but no stream reaches had been placed on a state 303(d) list.

**TABLE 11:** Summary of SPARROW-Estimated Total Phosphorus Enrichment and 303(d) Listings in HUC8 Watersheds in the United States Pacific Northwest (2002 conditions)

	TP Enrichment Estimated by SPARROW					
		
Percentage of Stream Reach Length Placed on State 303(d) Lists Because of Impairment Related to Nutrient Enrichment	None	Very Low	Low	High	Very High	Total
None	4	12	16	21	19	72
<50%	3	17	28	46	44	138
>50%	0	0	0	3	5	8

Notes: SPARROW, SPAtially Referenced Regressions On Watershed attributes; TP, total phosphorus. Values indicate the number of watersheds.

There are at least two explanations for the inconsistency between our results and the prevalence of state 303(d) listings. One is that water-quality monitoring programs in the PNW have not collected the water-quality data needed to adequately assess all watersheds with regards to nutrient enrichment. The U.S. Government Accountability Office (USGAO) found that only six states in the country reported having a majority of the data needed to fully assess all their waters, and that less than half the states had a majority of the data needed to determine if the waters that have been assessed should be placed on their 303(d) lists (USGAO, 2001). A second reason could be that the states in the PNW are using criteria besides nutrient concentrations to assess the impacts from nutrient enrichment, since water-quality impairment is not necessarily related to high nutrient concentrations. In addition to primary chemical indicators of nutrient enrichment (nitrogen and phosphorus concentrations), all states in the PNW consider secondary chemical indicators (dissolved oxygen concentration and pH) when assessing the impacts from nutrient enrichment and two states also consider biological indicators (algal growth and chlorophyll *a* concentration). Also, our compilation of 303(d)-listed streams likely underestimated the extent of nutrient impairment identified by the states because we only included reaches at the highest level of concern (i.e., impairment had been identified but no TMDL had been implemented). The state 303(d) lists actually have five categories, one of which includes impaired water bodies that do not require a TMDL because other control strategies are in place or a TMDL has already been implemented (Personal conversation with Tracy Chellis, USEPA, January 31, 2011).

One limitation of the USEPA criteria is that they do not account for the seasonal variability in stream conditions. The lowest streamflows throughout the PNW typically occur during summer (some exceptions are agricultural return drains in irrigated arid areas). The potential for water-quality impairment from nutrient enrichment is greatest during the summer low-flow period because solar radiation available for plant growth and water temperatures are higher than during other times of the year. As a result, mean seasonal, rather than mean annual, concentrations of nutrients might be more appropriate benchmarks for assessing nutrient enrichment in surface waters of the PNW. Future assessments of nutrient conditions in the PNW might address this limitation by calibrating SPARROW models for mean seasonal conditions. It is worth noting, however, that season-specific SPARROW calibrations are not currently documented.

Another limitation of USEPA nutrient criteria is that, even though they are intended to protect surface waters from the negative effects of nutrient enrichment, they may not represent reference conditions (i.e., those in undisturbed watersheds) because they are based solely on the 25th percentile of all the concentration data that were available in each level III ecoregion. Alternative approaches to developing nutrient criteria have been proposed to address this limitation (Smith *et al.*, 2003; Donigian *et al.*, 2005; Reckhow *et al.*, 2005; Herlihy and Sifneos, 2008). These approaches include the use of concentrations measured in reference streams coupled with predictive ecosystem models that relate the protection of designated uses to specific concentrations of nutrients. Also, there are limitations to using TN and TP as indicators of nutrient enrichment since they include some nitrogen and phosphorus that is not available for biological uptake. While we acknowledge that the recommended USEPA nutrient criteria have some limitations and that refined criteria might be more useful to water-quality managers, the USEPA criteria did provide a consistent and systematic baseline for assessing nutrient enrichment across the PNW.

The focus of this paper was nutrient conditions in freshwater streams, but the models we developed have applicability to other regional issues as well. For example, nutrient enrichment has been identified as a problem in some of the estuaries along the Pacific coasts of Washington and Oregon (especially the PUGT basin), but not all estuaries have detailed data on nutrient inputs (Bricker *et al.*, 2007; King County, 2010). Some of the nutrient load entering these estuaries is from watershed runoff, but much of it may come from the ocean (Brown and Ozretich, 2009). Our SPARROW models can help fill these data gaps by providing estimates of the nutrient loads to PNW estuaries from the land and the contribution from different sources to these nutrient loads.

## Concluding Remarks

We used the SPARROW model to complete the first regional assessment of surface-water nutrient conditions in the PNW. Our results clearly showed the spatial variation in nutrient yields and the relative levels of nutrient enrichment, as well as the primary sources of surface-water nutrients across the region. This regional interpretation of water-quality data could provide valuable information for water-quality management decisions in the PNW. Our predictions, however, might be improved through refinements in the stream network, the estimated stream loads, and the geospatial datasets used as inputs to the model.

## SUPPORTING INFORMATION

**Data S1.** SPARROW model – a detailed description of how the reach and watershed attribute data were compiled, and additional calibration and prediction results from the PNW SPARROW models.

Please note: Neither AWRA nor Wiley-Blackwell is responsible for the content or functionality of any supporting materials supplied by the authors. Any queries (other than missing material) should be directed to the corresponding author for the article.

## References

[b1] Aber JD (1992). Nitrogen Cycling and Nitrogen Saturation in Temperate Forest Ecosystems. Trends in Ecology and Evolution.

[b2] Alexander RB, Smith RA, Schwarz GE (2000). Effect of Stream Channel Size on the Delivery of Nitrogen to the Gulf of Mexico. Nature.

[b3] Alexander RB, Smith RA, Schwarz GE, Preston SD, Brakebill JW, Srinivasan R, Pacheco PA, Valigura Richard, Alexander Richard, Castro Mark, Meyers Tilden, Paerl Hans, Stacey Paul, Eugene Turner R (2001). Atmospheric Nitrogen Flux From the Watersheds of Major Estuaries of the United States: An Application of the SPARROW Watershed Model. Nitrogen Loading in Coastal Water Bodies: An Atmospheric Perspective.

[b4] Anderson CW (2002). Ecological Effects on Streams From Forest Fertilization—Literature Review and Conceptual Framework for Future Study in the Western Cascades.

[b5] Beaulac MN, Reckhow KH (1982). An Examination of Land Use – Nutrient Export Relationships. Water Resources Bulletin.

[b6] Bisson PA, Ice GI, Perrin CJ, Bilby RE, Miller RE (1992). Effects of Forest Fertilization on Water Quality and Aquatic Resources in the Douglas-Fir Region. Forest Fertilization: Sustaining and Improving Nutrition and Growth of Western Forests.

[b7] Brakebill JW, Wolock DM, Terziotti SE Digital Hydrologic Networks Supporting Applications Related to Spatially Referenced Regression Modeling. Journal of the American Water Resources Association.

[b8] Bricker S, Longstaff B, Dennison W, Jones A, Boicourt K, Wicks C, Woerner J (2007). Effects of Nutrient Enrichment in the Nation's Estuaries: A Decade of Change.

[b9] Brown CA, Ozretich RJ (2009). Coupling Between the Coastal Ocean and Yaquina Bay, Oregon: Importance of Oceanic Inputs Relative to Other Nitrogen Sources. Estuaries and Coasts.

[b10] Brown GW, Gahler AR, Marston RB (1973). Nutrient Losses After Clear-Cut Logging and Slash Burning in the Oregon Coast Range. Water Resources Research.

[b11] Chappell HN, Cole DW, Gessel SP, Walker RB (1991). Forest Fertilization Research and Practice in the Pacific Northwest. Fertilizer Research.

[b12] Clark GM (1997). Assessment of Nutrients, Suspended Sediment and Pesticides in Surface Water of the Upper Snake River Basin, Idaho and Western Wyoming, Water Years 1991-95.

[b13] Compton JA, Church MR, Larned ST, Hogsett WE (2003). Nitrogen Export From Forested Watersheds in the Oregon Coast Range: The Role of N_2_-Fixing Red Alder. Ecosystems.

[b14] Donigian AS, Love JT, Clough JS, Park RA, Carleton JN, Cocca PA, Imhoff JC (2005). Nutrient Criteria Development With a Linked Modeling System: Watershed and Ecological Model Application and Linkage.

[b15] Ebbert JC, Embrey SS, Kelley JA (2003). Concentrations and Loads of Sediment and Nutrients in Surface Water of the Yakima River Basin, Washington, 1999-2000 – With an Analysis of Trends in Concentrations.

[b16] Edmonds RL, Thomas TB, Blew RD (1995). Biogeochemistry of an Old-Growth Forested Watershed, Olympic National Park, Washington. Water Resources Bulletin.

[b17] Embrey SS, Inkpen EL (1998). Nutrient Transport in Rivers of the Puget Sound Basin, Washington 1980-1993.

[b18] Gardner RG (1990). The Role of Rock Weathering in the Phosphorus Budget of Terrestrial Watersheds. Biogeochemistry.

[b19] Gravelle JA, Ice G, Link TE, L Cook D (2009). Nutrient Concentration Dynamics in an Inland Pacific Northwest Watershed Before and After Timber Harvest. Forest Ecology and Management.

[b20] Harrington CA, Deal RL, Harrington CA (2006). Biology and Ecology of Red Alder. Red Alder: A State of Knowledge.

[b21] Herlihy AT, Sifneos JC (2008). Developing Nutrient Criteria and Classification Schemes for Wadeable Streams in the Conterminous U.S. Journal of the North American Benthological Society.

[b22] Homer C, Huang C, Yang L, Wylie B, Coan M (2004). Development of a 2001 National Landcover Database for the United States. Photogrammetric Engineering and Remote Sensing.

[b23] Hoos AB, McMahon G (2009). Spatial Analysis of Instream Nitrogen Loads and Factors Controlling Nitrogen Delivery to Streams in the Southeastern United States Using Spatially Referenced Regressions on Watershed Attributes (SPARROW) and Regional Classification Frameworks. Hydrological Processes.

[b24] Keller AK, Cavallaro L (2007). Assessing the U.S. Clean Water Act 303(d) Listing Process for Determining Impairment of a Waterbody. Journal of Environmental Management.

[b25] King County (2010). Initial Assessment of Nutrient Loading to Quartermaster Harbor.

[b26] LEMMA (Landscape, Ecology, Modeling, Mapping and Analysis) (2008). Interagency Mapping and Assessment Project. http://www.fsl.orst.edu/lemma/main.php?project=imap&id=studyAreas.

[b27] Martin CW, Harr RD (1988). Precipitation and Streamwater Chemistry From Undisturbed Watersheds in the Cascade Mountains of Oregon. Water, Air, and Soil Pollution.

[b28] Maupin MA, Ivahnenko T Nutrient Loadings to Streams of the Continental United States From Municipal and Industrial Effluent. Journal of the American Water Resources Association.

[b29] Moore RB, Johnston CM, Robinson KW, Deacon JR (2004). Estimation of Total Nitrogen and Phosphorus in New England Streams Using Spatially Referenced Regression Models.

[b30] del Moral R, Fleming RS (1979). Structure of Coniferous Forest Communities in Western Washington: Diversity and Ecotype Properties. Vegetation.

[b31] NADP (National Atmospheric Deposition Program) (2011). http://nadp.sws.uiuc.edu/.

[b32] National Research Council (2001). Assessing the TMDL Approach to Water Quality Management.

[b33] Neff JC, Holland EA, Dentener FJ, McDowell WH, Russell KM (2002). The Origin, Composition and Rates of Organic Nitrogen Deposition: A Missing Piece of the Nitrogen Cycle?. Biogeochemistry.

[b34] Nolan JV, Brakebill JW, Alexander RB, Schwarz GE (2002). Enhanced River Reach File 2. http://water.usgs.gov/GIS/metadata/usgswrd/XML/erf1_2.xml.

[b35] NRCS (Natural Resource Conservation Service) (1997). Washington Supplement to the National Irrigation Handbook, Part 652, Irrigation Guide.

[b36] Peterson BJ, Wollheim WM, Mulholland PJ, Webster JR, Meyer JL, Tank JL, Marti E, Bowden WB, Valett HM, Hershey AE, McDowell WH, Dodds WK, Hamilton SK, Gregory Stanley, Morrall DD (2001). Control of Nitrogen Export From Watersheds by Headwater Streams. Science.

[b37] Preston SD, Alexander RB, Woodside MD, Hamilton PA (2009). SPARROW Modeling—Enhancing Understanding of the Nation's Water Quality.

[b38] Preston SD, Brakebill JW (1999). Application of Spatially Referenced Regression Modeling for the Evaluation of Total Nitrogen Loading in the Chesapeake Bay Watershed.

[b39] Reckhow KH, Arhonditsis GB, Kenney MA, Hauser L, Tribo J, Wu C, Elcock KJ, Steinberg LJ, Stow CA, McBride SJ (2005). A Predictive Approach to Nutrient Criteria. Environmental Science & Technology.

[b40] Ruddy BC, Lorenz DL, Mueller DK (2006). County-Level Estimates of Nutrient Inputs to the Land Surface of the Conterminous United States, 1982-2001.

[b41] Rupert MG (1996). Major Sources of Nitrogen Input and Loss in the Upper Snake River Basin, Idaho and Western Wyoming, 1990.

[b42] Saad DA, Schwarz GE, Robertson DM, Booth NL A Multi-Agency Nutrient Dataset Used to Estimate Loads, Improve Monitoring Design, and Calibrate Regional Nutrient SPARROW Models. Journal of the American Water Resources Association.

[b43] Schaefer SC, Hollibaugh JT (2009). Watershed Nitrogen Input and Riverine Export on the West Coast of the U.S. Biogeochemistry.

[b44] Schwarz GE, Hoos AB, Alexander RB, Smith RA (2006). The SPARROW Surface Water-Quality Model—Theory, Applications and User Documentation.

[b45] Smith RA, Schwarz GE, Alexander RB (1997). Regional Interpretation of Water-Quality Monitoring Data. Water Resources Research.

[b46] Smith RA, Schwarz GE, Alexander RB (2003). Natural Background Concentrations of Nutrients in Streams and Rivers of the Conterminous United States. Environmental Science & Technology.

[b47] U.S. Census Bureau (1990). Summary Tape File 3 (STF 3) – Sample Data. http://factfinder.census.gov/servlet/DatasetMainPageServlet?_ds_name=DEC_1990_STF3_&_program=DEC&_lang=en.

[b48] USDA (United States Department of Agriculture) Economic Research Service (2009). Environmental Interactions With Agricultural Production: Grazing Lands and Environmental Quality. http://www.ers.usda.gov/briefing/agandenvironment/grazinglands.htm.

[b49] USEPA (U.S. Environmental Protection Agency) (1997). Level III Ecoregions of the Conterminous United States (Revision of Omernik, 1987).

[b50] USEPA (U.S. Environmental Protection Agency) (2000). Nutrient Criteria Technical Guidance Manual—Rivers and Streams.

[b51] USGAO (U.S. General Accounting Office) (2001). Water Quality: Key EPA and State Decisions Limited by Inconsistent and Incomplete Data.

[b52] USGS (United States Geological Survey) (2004). The National Geochemical Survey – Database and Documentation. http://tin.er.usgs.gov/geochem/.

[b53] Vanderbilt KS, Lajtha K, Swanson FJ (2003). Biogeochemistry of Unpolluted Forested Watersheds in the Oregon Cascades: Temporal Patterns of Precipitation and Stream Nitrogen Fluxes. Biogeochemistry.

[b54] Wentz DA, Bonn BA, Carpenter KD, Hinkle SR, Janet ML, Rinella FA, Uhrich MA, Waite IR, Laenen A, Bencala KE (1998). Water Quality in the Willamette Basin, Oregon, 1991-95.

[b55] Wieczorek M, Lamotte A (2011). Attributes for MRB_E2RF1 Catchments by Major River Basins in the Conterminous United States (U.S. Geological Survey Digital Data Series DS-491). http://water.usgs.gov/nawqa/modeling/rf1attributes.html.

[b56] Williamson AK, Munn MD, Ryker SJ, Wagner RJ, Ebbert JC, Vanderpool AM (1998). Water Quality in the Central Columbia Plateau, Washington and Idaho, 1992-95.

[b57] Wise DR, Rinella FA, Rinella JR, Fuhrer GJ, Embrey SS, Clark GM, Schwarz GE, Sobieszczyk S (2007). Nutrient and Suspended-Sediment Transport and Trends in the Columbia River and Puget Sound Basins, 1993-2003.

[b58] Wolock DM (2003). Hydrologic Landscape Regions of the United States. http://water.usgs.gov/lookup/getspatial?hlrus.

[b59] WRI (World Resources Institute) (2011). Eutrophication and Hypoxia – Sources of Nutrient Pollution. http://www.wri.org/project/eutrophication/about/sources.

