# Supplementary material for: Surface-Water Nutrient Conditions and Sources in the United States Pacific Northwest
Source: J Am Water Resour Assoc. 2011 Oct;47(5):1110–35. doi: 10.1111/j.1752-1688.2011.00580.x (PMC3307616; doi:10.1111/j.1752-1688.2011.00580.x)
Supplement: Supplementary file 1 [file jawr0047-1110-SD1.pdf]

Supplemental Information

SPARROW Model Background

SPARROW models are calibrated by estimating model coefficients that minimize the difference between the predicted and observed mean annual stream loads at a large number of fixed monitoring stations that are widely distributed throughout and representative of a hydrologic region. To maximize the explanatory power of the models, the mean annual stream loads must be compiled from as many sites as possible and often including sites where data are not collected contemporaneously. To account for differences in record length and sample size between stations, the water-quality records for the stations are detrended to reflect conditions during a single base year (Preston *et al*., 2009). The base year (2002 for our analysis) is selected to provide the greatest overlap between water-quality and streamflow records and is usually consistent with the temporal availability of the explanatory data used in the model. The detrended, mean annual stream loads are also normalized to long-term, mean hydrologic conditions to account for short-term spatial variability in precipitation across a region. This means that the measured stream loads used to calibrate a SPARROW model can be interpreted as the mean annual stream load that would have occurred in a specified base year if the mean annual streamflow for that year was equal to the long-term mean annual streamflow for the period of record.

Stream Reach Attributes

The SPARROW model includes a variable that allows the user to simulate the diversion of streamflow in a hydrologic network (the “frac value”, which is the fraction of streamflow that is delivered from one reach to the reach immediately downstream). We employed three methods for estimating frac values. The first, and preferred method, was to compare the measured, long-

term, mean annual streamflow between gages located upstream and downstream from known irrigation diversions and to calculate the ratio between the downstream and upstream values. When the upstream and downstream gages were separated by more than one reach, the frac value was scaled equally over the intervening reaches. When only one gage was available with long-term, mean annual streamflow and it was located above known irrigation diversions for which estimates could be made of the long-term mean annual discharge, the frac value was calculated by subtracting the total discharge for the diversions from the value for the upstream gage and dividing this value by the value for the upstream gage. The last, and least preferred method, was to compare the estimated long-term, mean streamflow for sequential reaches on the RF1 network (Brakebill *et al*., 2011). A frac value less than 1.0 was assigned to any reach in which there was a decrease in estimated streamflow between that reach and the reach immediately upstream. The approach we used to account for irrigation diversions in the PNW caused nutrient stream load to be removed from the modeled stream network, but did not return any of that stream load back to the network because we did not have the data needed to estimate this. Most of the water used for irrigation is taken up by crops or is lost through evaporation, and some leaches into deep groundwater. The remaining water, along with nutrients originally in the water and nutrients collected as it passes over agricultural land, is returned directly to streams through irrigation return drains or through shallow groundwater. Although our estimates of the frac values likely did not provided a full accounting of the hydrologic budget for the PNW, the values were based on the best available information and led to a more accurate model than otherwise would have been possible without including them.

Table S1 and Figure S1 provide details on the 303(d) listing in the PNW related to nutrient enrichment.

Table S1. Compilation of Stream Reaches Placed on State 303(d) Lists in the United States Pacific Northwest Because of Impairment Related to Nutrient Enrichment.

Figure S1. Prevalence of 303(d) Listings Because of Impairment Related to Nutrient Enrichment in the United States Pacific Northwest.

Watershed Attributes

The watershed attributes used in the PNW SPARROW models are shown in Figures S2 – S14.

Figure S2. Dominant Land Use in the United States Pacific Northwest (2001).

Figure S3. Point Source Discharges of Total Nitrogen in the United States Pacific Northwest (2002 estimates).

Figure S4. Point Source Discharges of Total Phosphorus in the United States Pacific Northwest (2002 estimates).

Figure S5. Mean Annual Wet Deposition of Inorganic Nitrogen in the United States Pacific Northwest.

Figure S6. Nitrogen Input from Farm Fertilizer in the United States Pacific Northwest (2002 estimates).

Figure S7. Phosphorus Input from Farm Fertilizer in the United States Pacific Northwest (2002 estimates).

Figure S8. Nitrogen Input from Manure from Livestock Production in the United States Pacific Northwest (2002 estimates).

Figure S9. Phosphorus Input from Manure from Livestock Production in the United States Pacific Northwest (2002 estimates).

Figure S10. Mean Soil Permeability in the United States Pacific Northwest.

Figure S11 Percentage of Land Area Consisting of Hydrologic Landscape Region 20 in the United States Pacific Northwest.

Figure S12. Effective Mean Annual Precipitation in the United States Pacific Northwest.

Figure S13. Basal Density of Red Alder Trees in the United Stated Pacific Northwest (2001 estimates).

Figure S14. Estimated Concentration of Phosphorus in Surficial Geologic Material in the United States Pacific Northwest.

The dominant land use was determined using data from the National Land Cover Database (NLCD (Homer *et al*., 2004). The area of forest land in each watershed was estimated by summing the area of land classified as deciduous, evergreen, and mixed forests in the NLCD. The area of developed land in each watershed was estimated by summing the area of land classified as low, medium, and highly developed in the NLCD. Developed land classified as open space in the NLCD was not included in our estimate because this category included roads in nondeveloped watersheds. In addition, the area of developed land in watersheds where the population density was less than 10 people per km^2 was set to zero.

The total red alder basal area within each SPARROW watershed was estimated for 2001. The red alder species models obtained from the Landscape, Ecology, Modeling, Mapping and Analysis (LEMMA) project (LEMMA, 2008 for Oregon; Mathew Gregory, Oregon State University, written commun., September 8, 2008 for Washington)were raster files with 30 meter cells. Estimates of red alder basal area density were extracted from each of the 5 raster files and

merged into a single raster file, from which the total red alder basal area in each SPARROW watershed was estimated.

The phosphorus sampling performed by the National Geochemical Survey (USGS, 2004) was limited to select streams in the PNW. Therefore, a value for each SPARROW watershed needed to be estimated by using landscape data that were spatially continuous across the region. This was done by combining data describing surface geology (Schruben *et al*., 1997) and USEPA level 4 ecoregion (Plate 1) (USEPA, 2008). The different surficial geologic materials were presumed to have different phosphorus contents (because of different mineral compositions) and the different USEPA level 4 ecoregions were presumed to have different potential for weathering of surficial geologic material (because of differences in geology, physiography, vegetation, climate, hydrology, terrestrial and aquatic fauna, and soil). We developed a layer of "geo-eco regions," calculated an average concentration of phosphorus for each geo-eco region from the available bed sediment data (expressed in parts per million), and calculated a value for each SPARROW watershed by using a weighted average for each geo-eco region in that watershed.

Plate 1. U.S. EPA Level 3 and Level 4 Ecoregions in the United States Pacific Northwest.

Calibration Results

Figures S15 – S22 compare predicted stream loads to measured stream loads, predicted stream loads to model residuals, and predicted yields to model residuals.

Figure S15. Measured Stream Load Relative to Predicted Stream Load for the Total Nitrogen SPARROW Model Developed for the United States Pacific Northwest.

Figure S16. Residual Stream Load Relative to Stream Predicted Load to for the Total Nitrogen SPARROW Model Developed for the United States Pacific Northwest.

Figure S17. Residual Stream Load Relative to Predicted Yield for the Total Nitrogen SPARROW Model Developed for the United States Pacific Northwest.

Figure S18. Probability Plot of Standardized Residual Stream Load for the Total Nitrogen SPARROW Model Developed for the United States Pacific Northwest.

Figure S19. Measured Stream Load Relative to Predicted Stream Load for the Total Phosphorous SPARROW Model Developed for the United States Pacific Northwest.

Figure S20. Residual Stream Load Relative to Predicted Stream Load for the Total Phosphorous SPARROW Model Developed for the United States Pacific Northwest.

Figure S21. Residual Stream Load Relative to Predicted Yield for the Total Phosphorous SPARROW Model Developed for the United States Pacific Northwest.

Figure S22. Probability Plot of Standardized Residual Stream Load for the Total Phosphorous SPARROW Model Developed for the United States Pacific Northwest.

Prediction Results

Table S3 provides details for each of the regional and national studies that we compared to our predicted yields for watersheds dominated by different land cover types.

Table S2. Details for Regional and National Nutrient Yield Estimates.

Tables S3 – S5 show the SPARROW estimates of nutrient enrichment and a summary of nutrient-related 303(d) listings for each HUC8 watershed in the PNW.

Table S3. Estimation of Nutrient Enrichment and Summary of State 303(d) Listings Because of Impairment Related to Nutrient Enrichment within the Columbia River Basin.

Table S4. Estimation of Nutrient Enrichment and Summary of State 303(d) Listings Because of Impairment Related to Nutrient Enrichment within the Snake River Basin.

Table S5. Estimation of Nutrient Enrichment and Summary of State 303(d) Listings Because of Impairment Related to Nutrient Enrichment within the Puget Sound Basin, the Coast Ranges of Washington and Oregon Draining Directly to the Pacific Ocean, and the Closed Basins of Oregon.

Literature Cited

Alexander, R.B., Smith, R.A. Schwarz, G.E., Preston, S.D., Brakebill, J.W., Srinivasan, R., and Pacheco, P.A., 2001, Atmospheric Nitrogen Flux from the Watersheds of Major Estuaries of the United States: An Application of the SPARROW Watershed Model, In: Nitrogen Loading in Coastal Water Bodies: An Atmospheric Perspective, American Geophysical Union Monograph 57, Richard Valigura, Richard Alexander, Mark Castro, Tilden Meyers, Hans Paerl, Paul Stacey, and R. Eugene Turner (eds.) pp. 119-170.

Beaulac, M.N and K.H. Rechow, 1982. An Examination of Land Use – Nutrient Export Relationships. Water Resources Bulletin 18(6):1013-1024.

Brakebill, J.W., D.M. Wolock, and S.E. Terziotti, 2011. Digital Hydrologic Networks Supporting Applications Related To Spatially Referenced Regression Modeling. Journal of the American Water Resources Association. This issue.

Brown, C.A. and R.J. Ozretich, 2009. Coupling Between the Coastal Ocean and Yaquina Bay, Oregon: Importance of Oceanic Inputs Relative to Other Nitrogen Sources. Estuaries and Coasts 33:87-117.

Brown, G.W., A.R. Gahler, and R.B. Marston, 1973. Nutrient Losses after Clear-Cut Logging and Slash Burning in the Oregon Coast Range. Water Resources Research 9(5): 1450-1453.

California Environmental Protection Agency, 2002. California's 2002 CWA Section 303(d) List of Water Quality Limited Segments. http://www.waterboards.ca.gov/water_issues/ programs/ tmdl/303d_lists.shtml, *accessed* March 17, 2009.

Compton, J.A., M.R. Church, S.T. Larned, and W.E. Hogsett, 2003. Nitrogen Export from Forested Watersheds in the Oregon Coast Range: The Role of N_2-fixing Red Alder. Ecosytems 6:773-785.

Edmonds, R.L., T.B. Thomas, and R.D. Blew, 1995. Biogeochemistry of an Old-Growth Forested Watershed, Olympic National Park, Washington. Water Resources Bulletin 31(3):409-419.

Embrey, S.S., and E.L. Inkpen, 1998, Nutrient Transport in Rivers of the Puget Sound Basin, Washington 1980-1993: U.S. Geological Survey Water-Resources Investigation Report 97-4270, 30 p.

Gravelle, J.A., G. Ice, T.E. .Link, and D.L Cook, 2009. Nutrient Concentration Dynamics in an Inland Pacific Northwest Watershed Before and after Timber Harvest. Forest Ecology and Management 257:1663-1675.

Homer, C. C. Huang, L. Yang, B. Wylie and M. Coan. 2004. Development of a 2001 National Landcover Database for the United States. Photogrammetric Engineering and Remote Sensing, 70: 829-840.

Idaho Department of Environmental Quality, 2002. Idaho Integrated §303(d)/§305(b) Report (2002).

http://www.deq.state.id.us/water/data_reports/surface_water/monitoring/integrated_report.cfm, *accessed* March 17, 2009.

LEMMA (Landscape, Ecology, Modeling, Mapping and Analysis), 2008. Interagency Mapping and Assessment Project). http://www.fsl.orst.edu/lemma/main.php?project=common&id= mr&model_region =9&ref=imap, accessed August 27, 2008.

Martin, C.W. and R.D. Harr, 1988. Precipitation and Streamwater Chemistry from Undisturbed Watersheds in the Cascade Mountains of Oregon. Water, Air, and Soil Pollution 42:203-219.

Montana Department of Environmental Quality, 2002. Montana 303(d) Information (2002). http://cwaic.mt.gov/query.aspx, *accessed* March 17, 2009.

Nevada Division of Environmental Protection, 2004. Nevada's 303(d) Impaired Waters List (2004). http://ndep.nv.gov/BWQP/303DLIST.HTM, *accessed* March 17, 2009.

Oregon Department of Environmental Quality, 2002. Water Quality Assessment of Oregon (2002). http://www.deq.state.or.us/wq/assessment/assessment.htm, *accessed* March 17, 2009.

Preston, S.D., R.B. Alexander, M.D. Woodside, and P.A. Hamilton, 2009. SPARROW Modeling —Enhancing Understanding of the Nation's Water Quality: U.S. Geological Survey Fact Sheet 2009–3019, 6 p.

Schaefer, S.C and J.T. Hollibaugh, 2009. Watershed Nitrogen Input and Riverine Export on the West Coast of the U.S. Biogechemistry 92: 219-233.

Schruben, P.G., R.E. Arndt, and W.J. Bawiec, 1997. Geology of the Conterminous United States at 1:2,500,000 Scale — A Digital Representation of the 1974 P.B. King and H.M. Beikman Map: U.S. Geological Survey Digital Data Series DDS-11, available at http://pubs.usgs.gov/dds/dds11/kb.html, accessed September 15, 2008.

USEPA (U.S. Environmental Protection Agency), 2008. Level IV Ecoregions of the Conterminous United States. U.S. Environmental Protection Agency, Western Ecology Division. http://www.epa. gov/wed/ pages/ecoregions/level_iv.htm, *accessed* September 17, 2008.

USGS (United States Geological Survey), 2004. The National Geochemical Survey - Database and Documentation: U.S. Geological Survey Open-File Report 2004-1001. http://tin.er.usgs.gov/geochem/, accessed August 1, 2008.

Utah Department of Environmental Quality, 2006. Utah's 2006 Integrated Report: Volume II – 303(d) List of Impaired Streams. http://www.waterquality.utah.gov/documents/ 200_303d_submittal_3-31-06.pdf, *accessed* March 17, 2009.

Vanderbilt, K.S., Kate Lajtha, and F.J. Swanson, 2003. Biogeochemistry of Unpolluted Forested Watersheds in the Oregon Cascades: Temporal Patterns of Precipitation and Stream Nitrogen Fluxes. Biogeochemistry 62:87-117.

Washington Department of Ecology, 2002. Water Quality Assessment of Washington (2002). http://apps.ecy.wa.gov/wqawa2008/viewer.htm, accessed March 17, 2009.

Wyoming Department of Environmental Quality, 2002. Wyoming Watershed Program 303d list (2002). http://deq.state.wy.us/wqd/watershed/Downloads/TMDL/2-2227-doc.pdf, accessed March 17, 2009.

Table S1. Compilation of Stream Reaches Placed on State 303(d) Lists in the United States Pacific Northwest Because of Impairment Related to Nutrient Enrichment.

State	Date of 303(d) listings	Primary chemical indicators	Secondary chemical indicators	Biological indicators	Number of reaches	Total length of reaches (km)
California[1]	2002	na	na	na	0	0
Idaho[2]	2002	nutrients, nitrogen, phosphorus	dissolved oxygen, pH	none	358	4,160
Montana[3]	2002	nutrients, nitrate, nitrogen	dissolved oxygen	algal growth, chlorophyll *a*	150	1,305
Nevada[4]	2004	total phosphorus	dissolved oxygen, pH	none	29	152
Oregon[5]	2002	nitrate, phosphorus	dissolved oxygen, pH	algae, aquatic weeds	378	3,405
Utah[6]	2006	na	na	na	0	0
Washington[7]	2008	total nitrogen, total phosphorus	dissolved oxygen, pH	none	321	4,194
Wyoming[8]	2002	na	na	na	0	0
Oregon-Washington border (Columbia River)	2002	total nitrogen, nitrate, total phosphorus, phosphorus	dissolved oxygen, pH	algae, aquatic weeds	29	264
Oregon-Idaho border (Snake River)	2002	nutrients, nitrogen, nitrates, phosphorus	dissolved oxygen, pH	algae, aquatic weeds	37	376
Washington-Idaho border (Snake River)	2002	nutrients, total nitrogen, nitrogen, total phosphorus, phosphorus	dissolved oxygen, pH	none	3	24

Notes:
na: not applicable, since there were no 303(d) listed streams for the state in the modeling region.
km: kilometers
[1]California Environmental Protection Agency, 2002
[2]Idaho Department of Environmental Quality, 2002
[3]Montana Department of Environmental Quality, 2002
[4]Nevada Division of Environmental Protection, 2004
[5]Oregon Department of Environmental Quality, 2002
[6]Utah Department of Environmental Quality, 2006
[7]Washington Department of Ecology, 2002
[8]Wyoming Department of Environmental Quality, 2002

Table S2. Details for Regional and National Nutrient Yield Estimates.	
**Data Source**	**Description of Results**
Alexander *et al*., 2001	Median TN yields estimated by SPARROW for watersheds across the nation having more than 90% crop land and 85% pasture land.
	Median TN yields estimated by SPARROW for watersheds across the nation having more than 95% forest land.
	Median TN yields estimated by SPARROW for watersheds across the nation having more than 75% urban land.
	Median TN yields estimated by SPARROW for watersheds across the nation having 100% range land.
Beaulac and Rechow, 1982	Median TN and TP export for mixed agricultural land across the U.S.
	Median TN and TP export for pasture and range land across the U.S.
	Median TN and TP for urban land across the U.S.
Brown *et al*., 1973	Median NO3-N export from watersheds in the Alsea R. basin (Oregon).
Brown and Ozretich, 2009	Riverine N export to Yaquina Bay (Oregon).
Compton *et al*., 2003	Average N export from watersheds within the Salmon R. basin (Oregon).
Edmunds *et al*., 1995	TN and TP export from West Twin Creek watershed (Washington).
Embrey and Inkpen , 1998	Average inoganic N and TP export from three primarily agricultural basins draining to Puget Sound (Washington).
	Inoganic N and TP export from a primarily forested basin draining to Puget Sound (Washington).
	Average inoganic N and TP export from two primarily urban basins draining to Puget Sound (Washington).
Gravelle *et al*., 2009	Average NO3/NO2-N export from regenerated subwatersheds within the Mica Creek basin (Idaho).
	Average NO3/NO2-N export from clearcut subwatersheds within the Mica Creek basin (Idaho).
Martin and Harr, 1988	Nitrate-N and Phosphate-P export from an undisturbed forested watershed in the Oregon Cascades.
Schaefer *et al*., 2009	TN export for 2002 from the Spokane River basin (Washington).
	TN export for 1992 from the Nehalem River basin (Oregon).
Vanderbilt *et al*., 2003	Average TN export from unpolluted watersheds in the Oregon Cascades.
Wise, 2004 (unpublished)	TN and TP yields for small agricultural watersheds in the lower Yakima River Basin (Washington)

Notes:
TN: total nitrogen.
TP: total phosphorus.
NO3-N: nitrate, as nitrogen
N: nitrogen
NO3/NO2-N: nitrate plus nitrage, as nitrogen.
Phosphate-P: phosphate, as phosphorus.
SPARROW: Spatially Referenced Regressions on Watershed Attributes model.

Table S3. Estimation of Nutrient Enrichment and Summary of State 303(d) Listings Because of Impairment Related to Nutrient Enrichment within the Columbia River Basin.

Six-Digit Hydrologic Unit Code (HUC6)Watershed		Eight-Digit Hydrologic Unit Code (HUC8) Watershed		Percentage of stream reach length where the probability of exceeding the suggested USEPA TN criterion was greater than 90%	Percentage of stream reach length where the probability of exceeding the suggested USEPA TP criterion was greater than 90%	Percentage of stream reach length that is placed on state 303(d) lists because of impairment related to nutrient enrichment
170101	Kootenai River	17010101	Upper Kootenai	82.0	71.3	3.6
		17010102	Fisher	70.5	36.0	12.6
		17010103	Yaak	70.4	65.6	0.0
		17010104	Lower Kootenai	44.4	22.0	0.0
		17010105	Moyie	72.9	18.6	0.0
170102	Pend Oreille River	17010201	Upper Clark Fork	97.1	55.3	35.7
		17010202	Flint-Rock	84.0	58.0	4.0
		17010203	Blackfoot	72.3	41.7	14.5
		17010204	Middle Clark Fork	53.2	55.6	21.1
		17010205	Bitterroot	28.8	29.4	13.1
		17010206	North Fork Flathead	45.1	51.6	0.0
		17010207	Middle Fork Flathead	18.9	25.9	0.0
		17010208	Flathead Lake	30.4	11.6	0.0
		17010209	South Fork Flathead	48.2	46.2	0.0
		17010210	Stillwater	86.9	56.7	24.9
		17010211	Swan	82.2	68.7	2.4
		17010212	Lower Flathead	61.1	55.4	0.0
		17010213	Lower Clark Fork	32.2	45.3	2.0
		17010214	Pend Oreille Lake	56.2	67.7	9.5
		17010215	Priest	31.2	65.0	0.0
		17010216	Pend Oreille	58.2	67.1	69.9
170103	Spokane River	17010301	Upper Coeur D'alene	3.2	3.2	0.0
		17010302	South Fork Coeur D'alene	21.7	61.5	0.0
		17010303	Coeur D'alene Lake	40.2	60.8	0.8
		17010304	St. Joe	31.0	40.6	1.3
		17010305	Upper Spokane	5.0	20.1	44.0
		17010306	Hangman	100.0	100.0	34.6
		17010307	Lower Spokane	71.0	76.8	16.1
		17010308	Little Spokane	100.0	100.0	64.8
170300	Yakima River	17030001	Upper Yakima	36.5	60.3	10.8
		17030002	Naches	71.6	80.1	13.9
		17030003	Lower Yakima	35.8	76.0	14.7
Snake River (see table S5)						
170200	Upper Columbia River	17020001	Franklin D. Roosevelt Lake	72.9	73.7	10.4
		17020002	Kettle	88.0	79.0	8.4
		17020003	Colville	100.0	100.0	11.2
		17020004	Sanpoil	4.7	4.7	7.4
		17020005	Chief Joseph	3.4	3.4	0.7
		17020006	Okanogan	40.3	44.7	22.6
		17020007	Similkameen	3.0	1.1	6.9
		17020008	Methow	14.2	55.2	0.0
		17020009	Lake Chelan	0.0	8.5	3.9
		17020010	Upper Columbia-Entiat	7.4	10.2	1.6
		17020011	Wenatchee	1.0	14.5	16.2
		17020012	Moses Coulee	0.0	0.0	0.0
		17020013	Upper Crab	100.0	100.0	30.3
		17020014	Banks Lake	100.0	100.0	0.9
		17020015	Lower Crab	88.5	84.7	66.7
		17020016	Upper Columbia-Priest Rapids	0.0	36.0	32.5

Table S3 con't. Estimation of Nutrient Enrichment and Summary of State 303(d) Listings Because of Impairment Related to Nutrient Enrichment within the Columbia River Basin.						
**Six-Digit Hydrologic Unit Code (HUC6 Watershed**		**Eight-Digit Hydrologic Unit Code (UUC8) Watershed**		**Percentage of stream reach length where the probability of exceeding the suggested USEPA TN criterion was greater than 90%**	**Percentage of stream reach length where the probability of exceeding the suggested USEPA TP criterion was greater than 90%**	**Percentage of stream reach length that is placed on state 303(d) lists because of impairment related to nutrient enrichment**
170702	John Day River	17070201	Upper John Day	91.6	100.0	13.4
		17070202	North Fork John Day	90.6	96.6	0.0
		17070203	Middle Fork John Day	59.2	100.0	0.0
		17070204	Lower John Day	25.0	77.9	0.4
170703	Deschutes River	17070301	Upper Deschutes	65.8	81.8	22.4
		17070302	Little Deschutes	100.0	100.0	20.9
		17070303	Beaver-South Fork	18.0	79.2	0.0
		17070304	Upper Crooked	36.5	88.7	7.0
		17070305	Lower Crooked	76.2	86.3	18.1
		17070306	Lower Deschutes	54.0	72.3	12.0
		17070307	Trout	12.8	84.9	0.0
170701	Middle Columbia	17070101	Middle Columbia-Lake Wallula	4.5	11.1	0.3
		17070102	Walla Walla	79.8	100.0	0.0
		17070103	Umatilla	36.8	75.0	5.5
		17070104	Willow	35.3	73.3	19.7
		17070105	Middle Columbia-Hood	59.4	47.9	4.8
		17070106	Klickitat	63.1	87.5	0.0
170900	Willamette River	17090001	Middle Fork Willamette	9.1	74.6	2.7
		17090002	Coast Fork Willamette	20.3	57.8	3.6
		17090003	Upper Willamette	53.1	17.6	10.5
		17090004	McKenzie	10.1	74.0	5.2
		17090005	North Samtiam	4.5	60.4	5.2
		17090006	South Santiam	20.8	59.7	0.0
		17090007	Middle Willamette	66.7	21.7	26.5
		17090008	Yamhill	74.8	25.1	10.2
		17090009	Molalla-Pudding	90.9	58.7	0.0
		17090010	Tualatin	66.6	30.7	0.0
		17090011	Clackamas	29.9	73.7	0.0
		17090012	Lower Willamette	78.3	41.7	10.5
170800	Lower Columbia River	17080001	Lower Columbia-Sandy	35.4	56.7	26.4
		17080002	Lewis	26.8	71.9	0.0
		17080003	Lower Columbia-Clatskanie	91.9	86.8	21.5
		17080004	Upper Cowlitz	3.7	23.8	0.1
		17080005	Lower Cowlitz	54.2	69.3	0.0
		17080006	Lower Columbia	99.4	96.1	30.5

Table S4. Estimation of Nutrient Enrichment and Summary of State 303(d) Listings Because of Impairment Related to Nutrient Enrichment within the Snake River Basin.						
**Six-Digit Hydrologic Unit Code (HUC6) Watershed**		**Eight-Digit Hydrologic Unit Code (HUC8) Watershed**		**Percentage of stream reach length where the probability of exceeding the suggested USEPA TN criterion was greater than 90%**	**Percentage of stream reach length where the probability of exceeding the suggested USEPA TP criterion was greater than 90%**	**Percentage of stream reach length that is placed on state 303(d) lists because of impairment related to nutrient enrichment**
170401	Snake River Headwaters	17040101	Snake Headwaters	32.1	95.5	0.0
		17040102	Gros Ventre	35.2	82.5	0.0
		17040103	Greys-Hobock	10.8	65.5	0.0
		17040104	Pallisades	46.0	97.7	0.0
		17040105	Salt	73.0	100.0	3.5
170402	Upper Snake River	17040201	Idaho Falls	32.3	43.2	0.0
		17040202	Upper Henry's	60.2	78.8	0.0
		17040203	Lower Henry's	53.0	63.5	0.0
		17040204	Teton	45.3	72.9	7.5
		17040205	Willow	72.0	100.0	13.4
		17040206	American Falls	58.3	72.3	30.4
		17040207	Blackfoot	29.8	91.0	0.0
		17040208	Portneuf	65.8	99.0	9.0
		17040209	Lake Walcott	45.5	72.6	0.0
		17040210	Raft	71.4	94.1	20.7
		17040211	Goose	16.2	91.1	9.0
		17040212	Upper Snake-Rock	54.6	76.5	28.8
		17040213	Salmon Falls	9.2	98.1	16.5
		17040214	Beaver-Camas	82.2	100.0	35.6
		17040215	Medicine Lodge	51.6	90.6	0.7
		17040216	Birch	61.7	100.0	11.7
		17040217	Little Lost	87.5	100.0	0.2
		17040218	Big Lost	17.4	80.2	17.5
		17040219	Big Wood	9.7	62.5	10.8
		17040220	Camas	2.1	92.3	2.3
		17040221	Little Wood	5.6	48.2	38.5
170501	Middle Snake - Boise Rivers	17050101	C.J. Idaho	33.4	55.0	7.6
		17050102	Bruneau	0.8	56.2	1.0
		17050103	Middle Snake-Succor	19.3	43.4	14.0
		17050104	Upper Owyhee	0.0	95.9	6.9
		17050105	South Fork Owyhee	4.4	93.3	0.0
		17050106	East Little Owyhee	0.0	100.0	0.0
		17050107	Middle Owyhee	3.7	86.5	2.8
		17050108	Jordan	18.3	99.3	2.6
		17050109	Crooked-Rattlesnake	5.1	92.7	0.0
		17050110	Lower Owyhee	8.9	98.6	10.1
		17050111	North and Middle Forks Boise	0.0	55.1	0.0
		17050112	Boise-Mores	2.7	53.5	0.0
		17050113	South Fork Boise	0.0	14.5	0.0
		17050114	Lower Boise	62.5	80.6	67.3
		17050115	Middle Snake-Payette	72.8	72.8	35.6
		17050116	Upper Malheur	45.6	100.0	0.0
		17050117	Lower Malheur	22.4	100.0	19.4
		17050118	Bully	10.1	100.0	3.5
		17050119	Willow	57.4	98.6	8.1
		17050120	South Fork Payette	0.0	5.2	0.0
		17050121	Middle Fork Payette	0.0	0.0	0.0
		17050122	Payette	0.6	18.2	20.2
		17050123	North Fork Payette	0.0	0.0	35.4
		17050124	Weiser	22.9	79.8	30.0
170502	Middle Snake - Powder Rivers	17050201	Brownlee Reservoir	78.6	92.7	30.7
		17050202	Burnt	73.1	100.0	8.2
		17050203	Powder	78.4	97.6	11.6

Table S4 con't. Estimation of Nutrient Enrichment and Summary of State 303(d) Listings Because of Impairment Related to Nutrient Enrichment within the Snake River Basin.						
**Six-Digit Hydrologic Unit Code (HUC6 Watershed**		**Eight-Digit Hydrologic Unit Code (HUC8) Watershed**		**Percentage of stream reach length where the probability of exceeding the suggested USEPA TN criterion was greater than 90%**	**Percentage of stream reach length where the probability of exceeding the suggested USEPA TP criterion was greater than 90%**	**Percentage of stream reach length that is placed on state 303(d) lists because of impairment related to nutrient enrichment**
170602	Salmon River	17060201	Upper Selway	0.0	32.6	5.8
		17060202	Pahsimeroi	0.0	67.3	25.9
		17060203	Middle Salmon-Panther	3.0	19.4	3.5
		17060204	Lemhi	69.7	89.0	6.3
		17060205	Upper Middle Fork Salmon	0.0	29.8	0.0
		17060206	Lower Middle Fork Salmon	0.0	1.5	0.0
		17060207	Middle Salmon-Chamberlain	1.9	15.3	0.0
		17060208	South Fork Salmon	0.0	7.3	0.0
		17060209	Lower Salmon	19.4	77.1	8.6
		17060210	Little Salmon	21.7	55.7	5.5
170603	Clearwater River	17060301	Upper Selway	0.0	0.0	0.0
		17060302	Lower Selway	0.0	10.6	0.0
		17060303	Lochsa	5.5	2.3	0.0
		17060304	Middle Fork Clearwater	13.4	38.2	18.5
		17060305	South Fork Clearwater	13.4	34.0	14.1
		17060306	Clearwater	66.0	83.5	40.2
		17060307	Upper North Fork Clearwater	24.2	27.6	0.0
		17060308	Lower North Fork Clearwater	11.8	34.8	4.7
170601	Lower Snake River	17060101	Hells Canyon	54.5	89.8	51.7
		17060102	Imnaha	67.0	100.0	0.0
		17060103	Lower Snake-Asotin	41.7	98.2	5.7
		17060104	Upper Grande Ronde	82.7	96.7	0.0
		17060105	Wallowa	52.4	91.1	14.9
		17060106	Lower Grande Ronde	63.6	100.0	0.0
		17060107	Lower Snake-Tucannon	41.6	82.7	23.4
		17060108	Palouse	77.5	88.4	37.1
		17060109	Rock	88.1	92.8	22.2
		17060110	Lower Snake	0.0	11.2	14.1

Table S5. Estimation of Nutrient Enrichment and Summary of State 303(d) Listings Because of Impairment Related to Nutrient Enrichment within the Puget Sound Basin, the Coast Ranges of Washington and Oregon Draining Directly to the Pacific Ocean, and the Closed Basins of Oregon.

**Six-Digit Hydrologic Unit Code (HUC6) Watershed**		**Eight-Digit Hydrologic Unit Code (HUC8) Watershed**		**Percentage of stream reach length where the probability of exceeding the suggested USEPA TN criterion was greater than 90%**	**Percentage of stream reach length where the probability of exceeding the suggested USEPA TP criterion was greater than 90%**	**Percentage of stream reach length that is placed on state 303(d) lists because of impairment related to nutrient enrichment**
171100	Puget Sound	Fraser	17110001	39.9	38.3	9.7
		Strait of Georgia	17110002	100.0	83.9	37.1
		San Juan Islands	17110003	na	na	na
		Nooksack	17110004	28.2	40.2	34.1
		Upper Skagit	17110005	4.8	14.0	0.0
		Sauk	17110006	1.3	33.9	0.0
		Lower Skagit	17110007	49.5	43.7	34.6
		Stillaquamish	17110008	22.6	31.5	2.6
		Skykomish	17110009	15.8	14.9	8.0
		Snoqualmie	17110010	17.9	5.0	11.4
		Snohomish	17110011	89.5	67.3	54.1
		Lake Washington	17110012	73.8	66.8	46.3
		Duwamish	17110013	57.9	87.5	54.1
		Puyallup	17110014	32.1	73.5	9.3
		Nisqually	17110015	62.7	74.8	11.1
		Deschutes	17110016	100.0	100.0	79.3
		Skokomish	17110017	17.7	62.4	0.0
		Hood Canal	17110018	8.1	13.2	10.9
		Puget Sound	17110019	95.8	91.5	9.0
		Dungeness-Elwha	17110020	5.1	25.6	0.0
		Crescent-Hoko	17110021	83.0	63.7	0.0
171001	Washington Coastal Rivers	Hoh-Quillayute	17100102	4.4	66.1	0.0
		Queets-Quinault	17100103	95.2	94.3	5.1
		Upper Chehalis	17100104	65.0	83.8	0.0
		Lower Chehalis	17100105	0.2	85.7	2.2
		Grays Harbor	17100106	91.0	90.9	3.5
		Willapa Bay	17100101	21.8	60.9	9.3
171002	Northern Oregon Coastal Rivers	Necanicum	17100201	100.0	100.0	0.0
		Nehalem	17100202	93.6	96.5	0.0
		Wilson-Trusk-Nestucca	17100203	100.0	94.2	33.3
		Siletz-Yaquina	17100204	97.2	70.1	8.0
		Alsea	17100205	95.7	81.6	8.3
		Siuslaw	17100206	72.8	62.6	23.6
		Siltcoos	17100207	94.8	83.3	0.0
171003	Southern Oregon Coastal Rivers	North Umpqua	17100301	27.8	80.1	7.7
		South Umpqua	17100302	34.3	59.3	22.0
		Umpqua	17100303	63.1	73.8	5.3
		Coos	17100304	88.2	77.2	10.5
		Coquille	17100305	71.4	46.9	20.3
		Sixes	17100306	98.7	100.0	8.1
		Upper Rogue	17100307	23.1	98.5	10.2
		Middle Rogue	17100308	47.3	73.0	5.0
		Applegate	17100309	4.5	38.6	12.5
		Lower Rogue	17100310	49.8	72.7	18.7
		Illinois	17100311	18.1	66.9	0.0
		Chetco	17100312	1.8	50.6	11.8
171200	Oregon Closed Basins	Harney-Malheur	17120001	68.1	85.0	0.0
		Silvies	17120002	84.4	95.0	5.6
		Donner und Blitzen	17120003	0.0	48.6	0.0
		Silver	17120004	22.5	33.0	0.0
		Summer Lake	17120005	86.5	91.5	0.0
		Lake Albert	17120006	54.0	73.5	0.0
		Warner Lakes	17120007	17.2	49.5	8.0
		Guano	17120008	0.0	11.6	0.0
		Alvord Lake	17120009	0.0	71.9	0.0

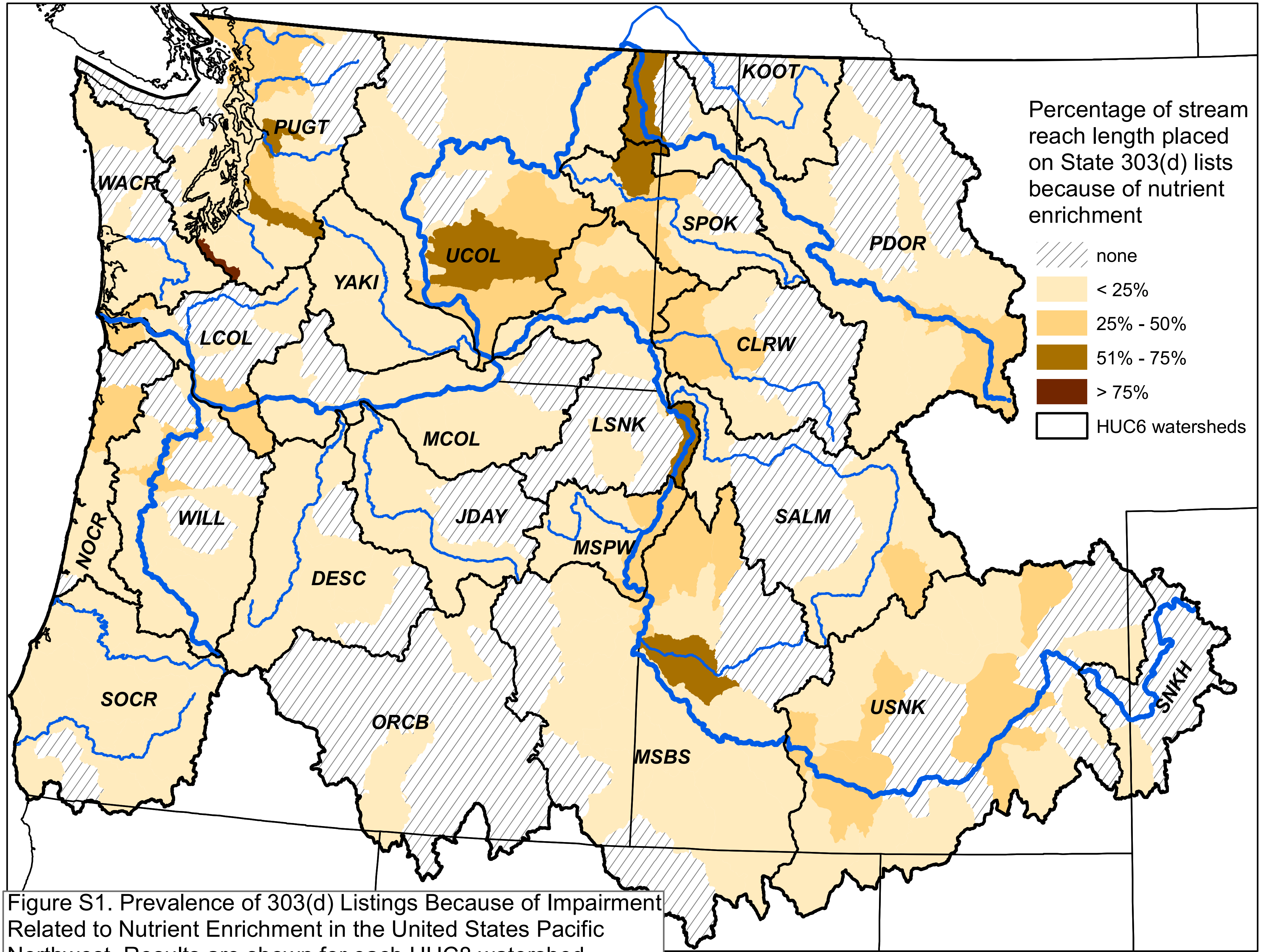

Figure S1. Prevalence of 303(d) Listings Because of Impairment Related to Nutrient Enrichment in the United States Pacific Northwest. Results are shown for each HUC8 watershed.

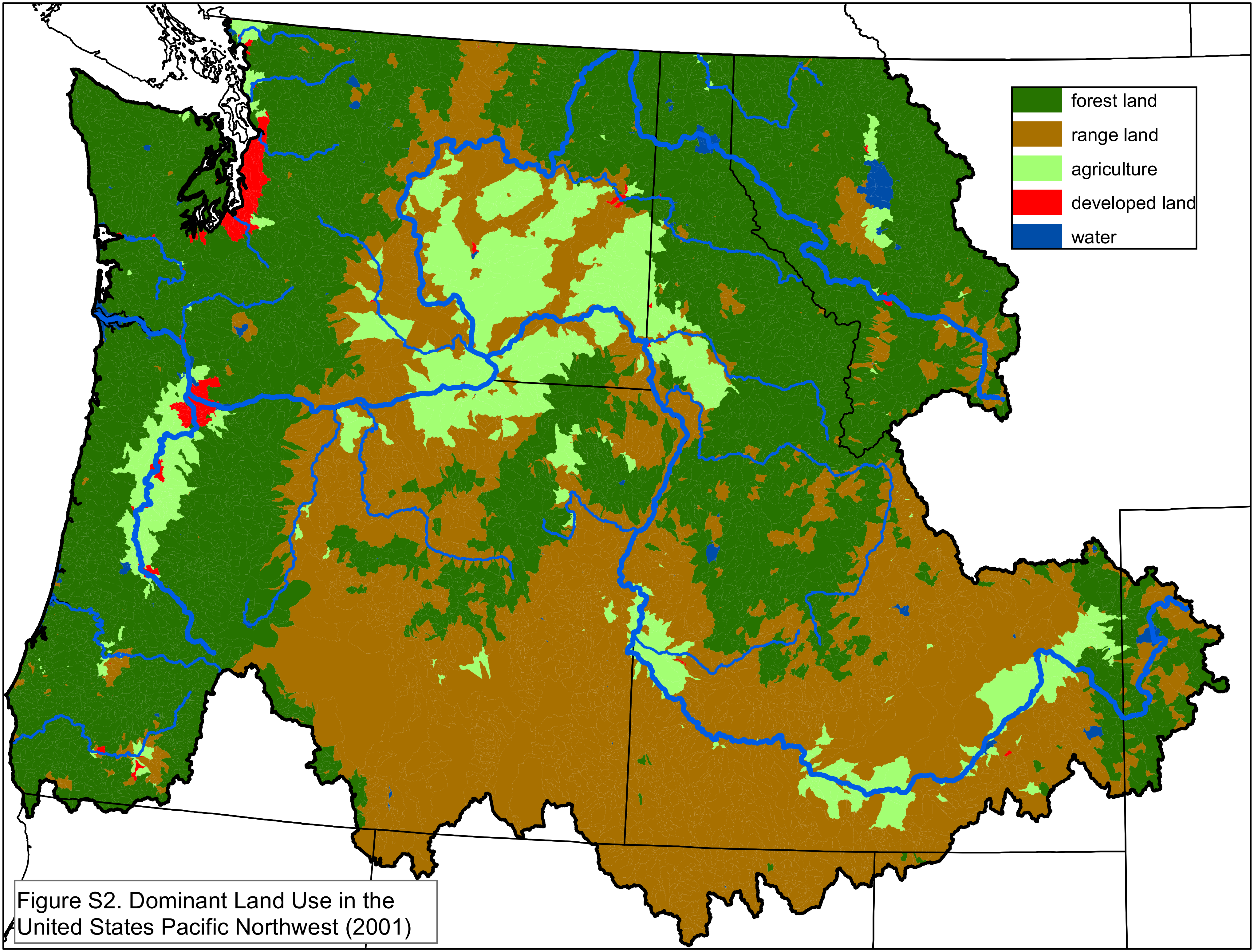

Figure S2. Dominant Land Use in the United States Pacific Northwest (2001)

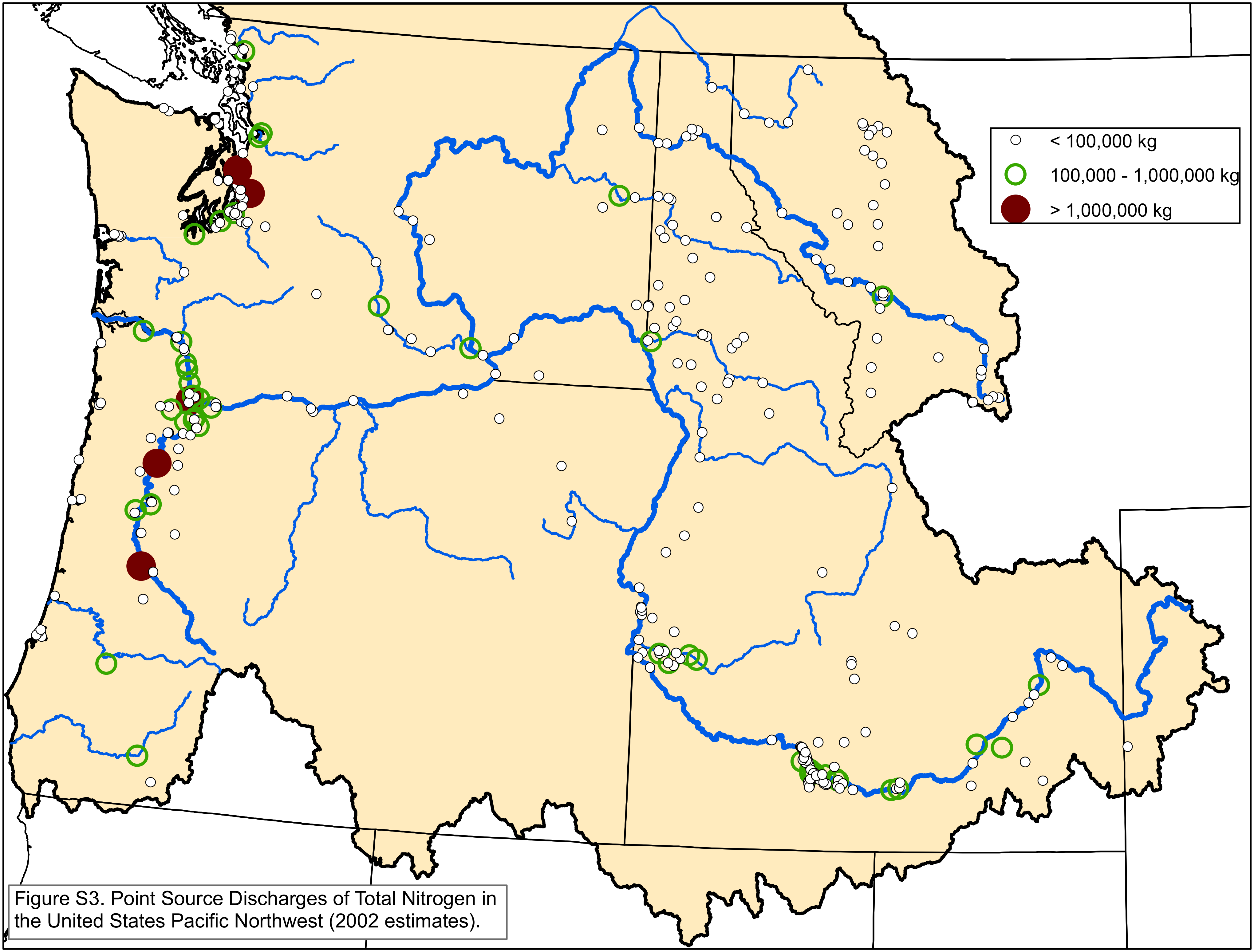

Figure S3. Point Source Discharges of Total Nitrogen in the United States Pacific Northwest (2002 estimates).

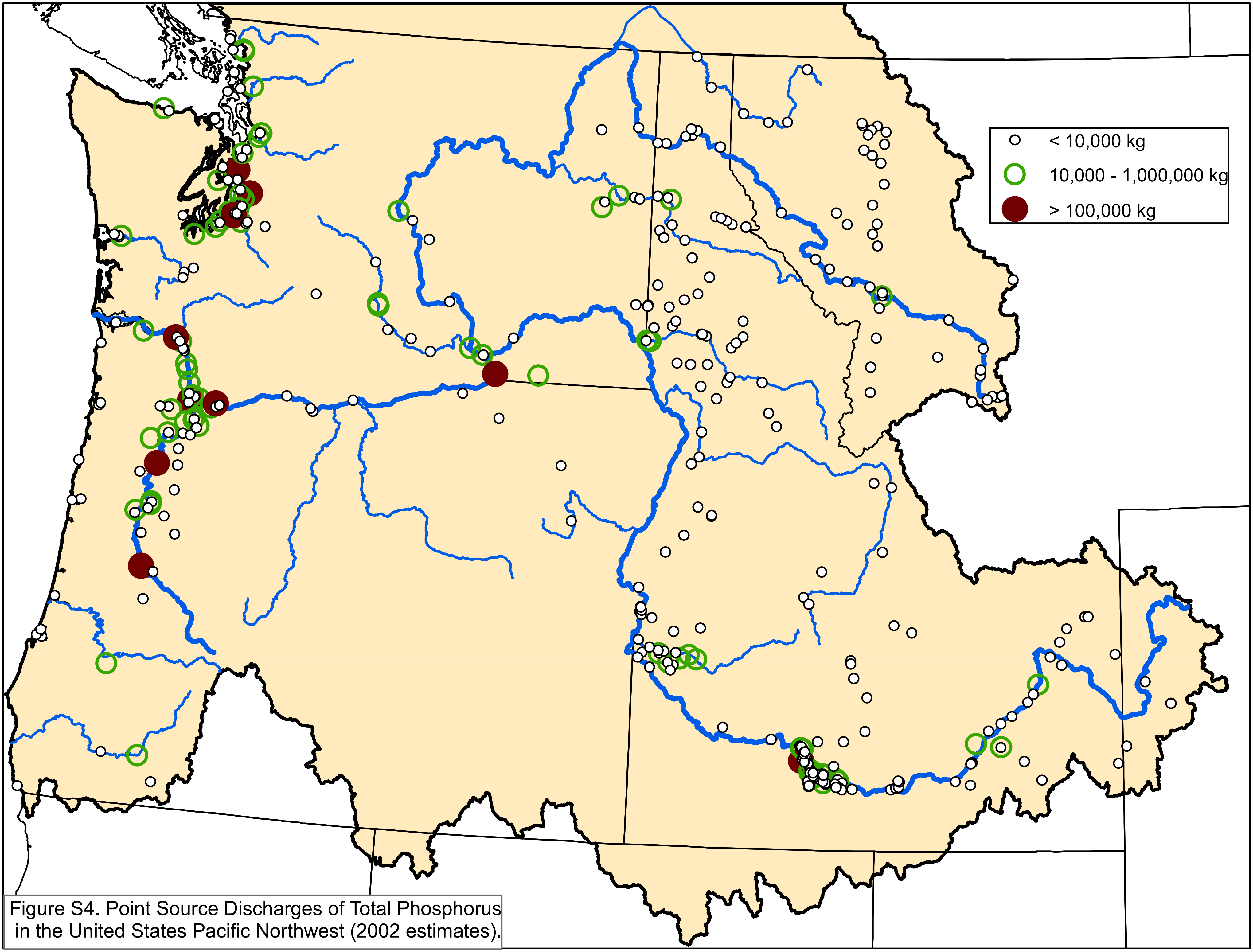

Figure S4. Point Source Discharges of Total Phosphorus in the United States Pacific Northwest (2002 estimates).

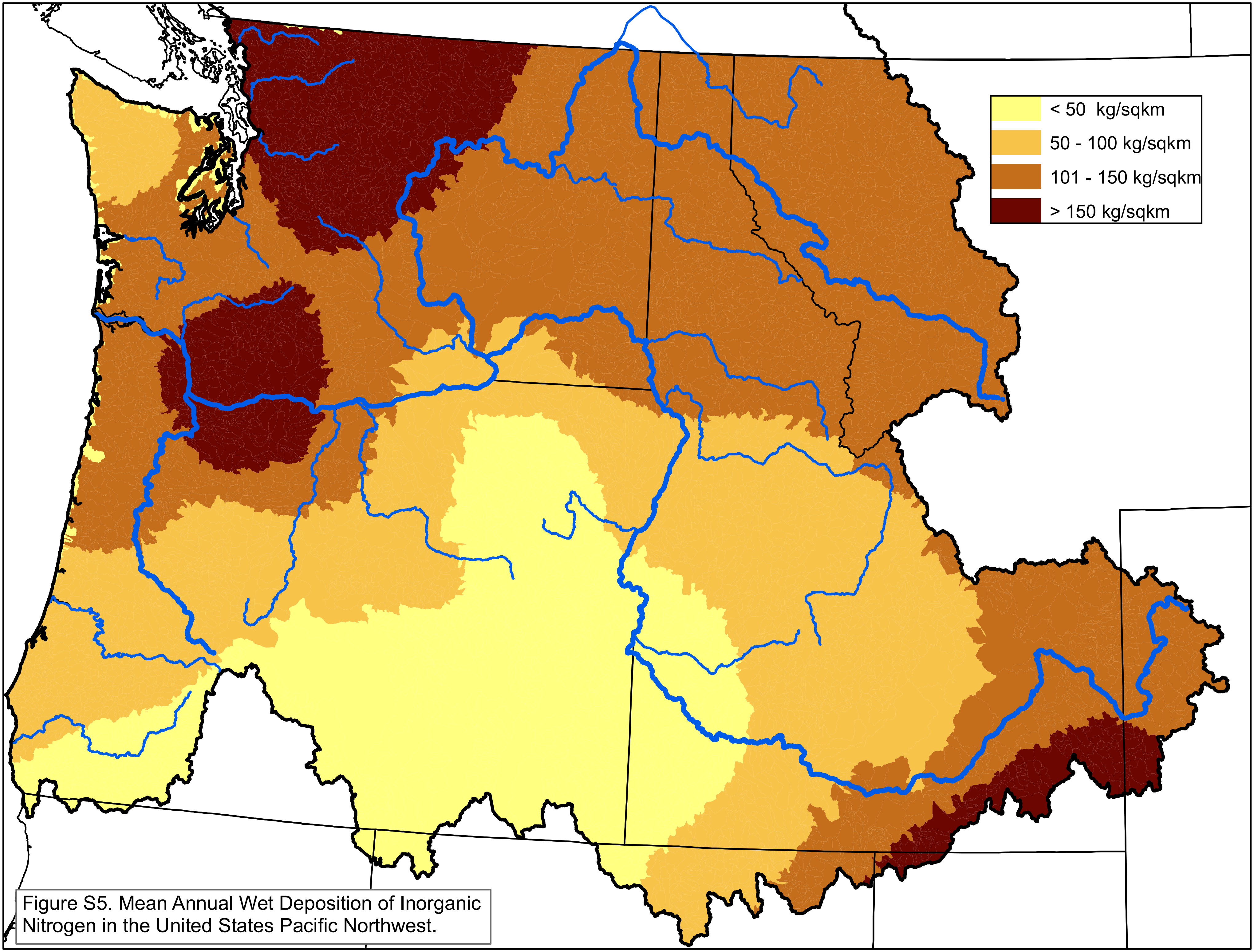

Figure S5. Mean Annual Wet Deposition of Inorganic Nitrogen in the United States Pacific Northwest.

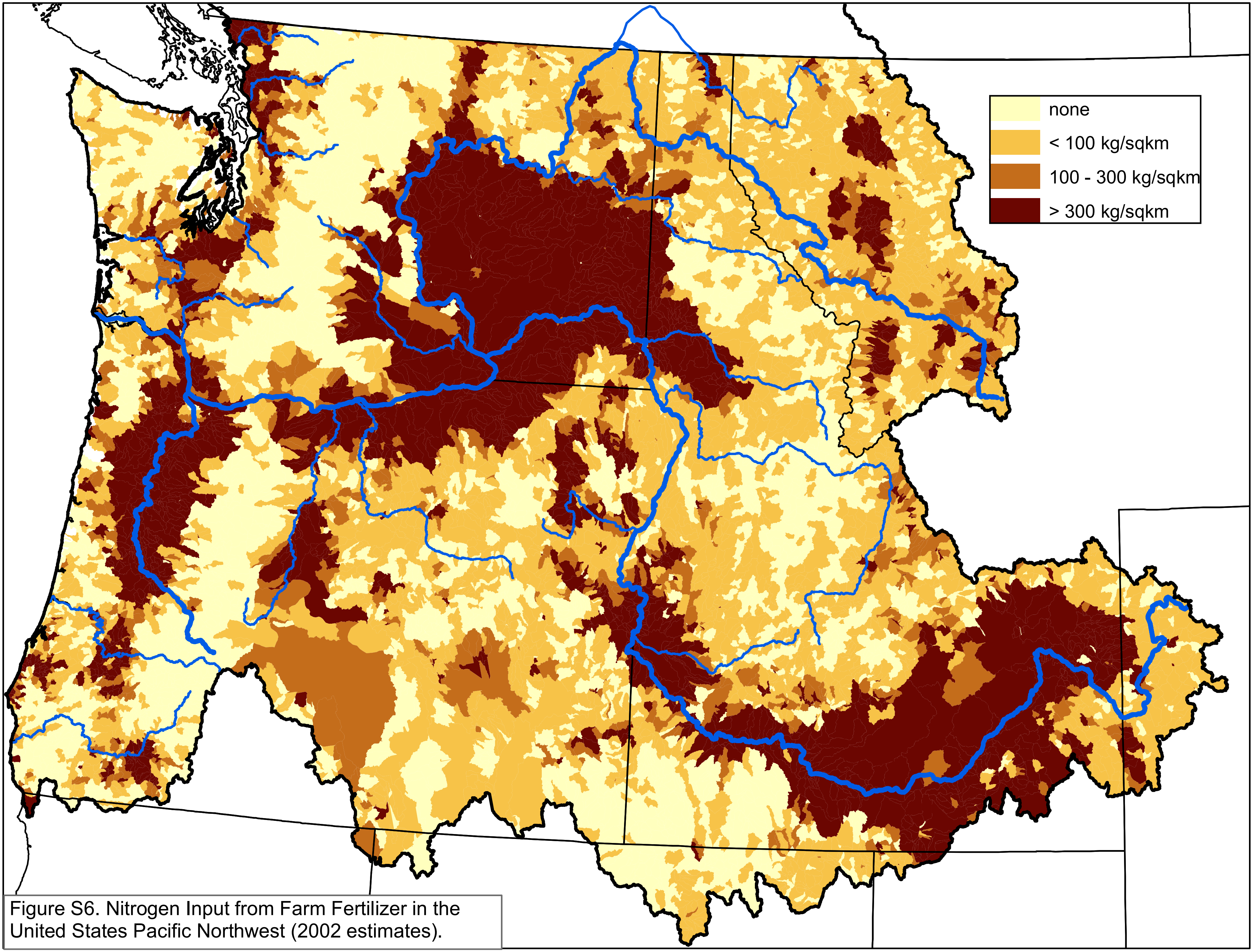

Figure S6. Nitrogen Input from Farm Fertilizer in the United States Pacific Northwest (2002 estimates).

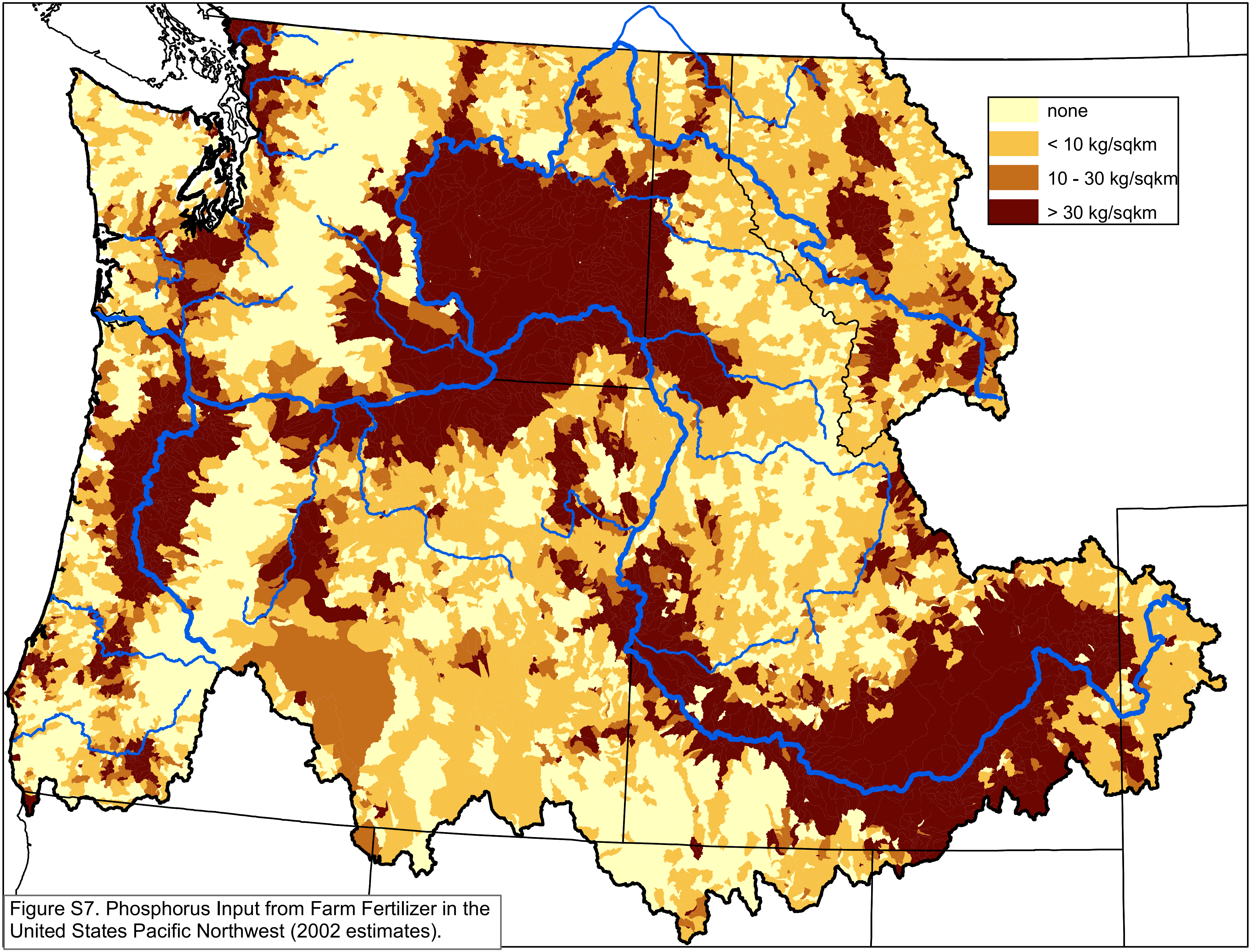

Figure S7. Phosphorus Input from Farm Fertilizer in the United States Pacific Northwest (2002 estimates).

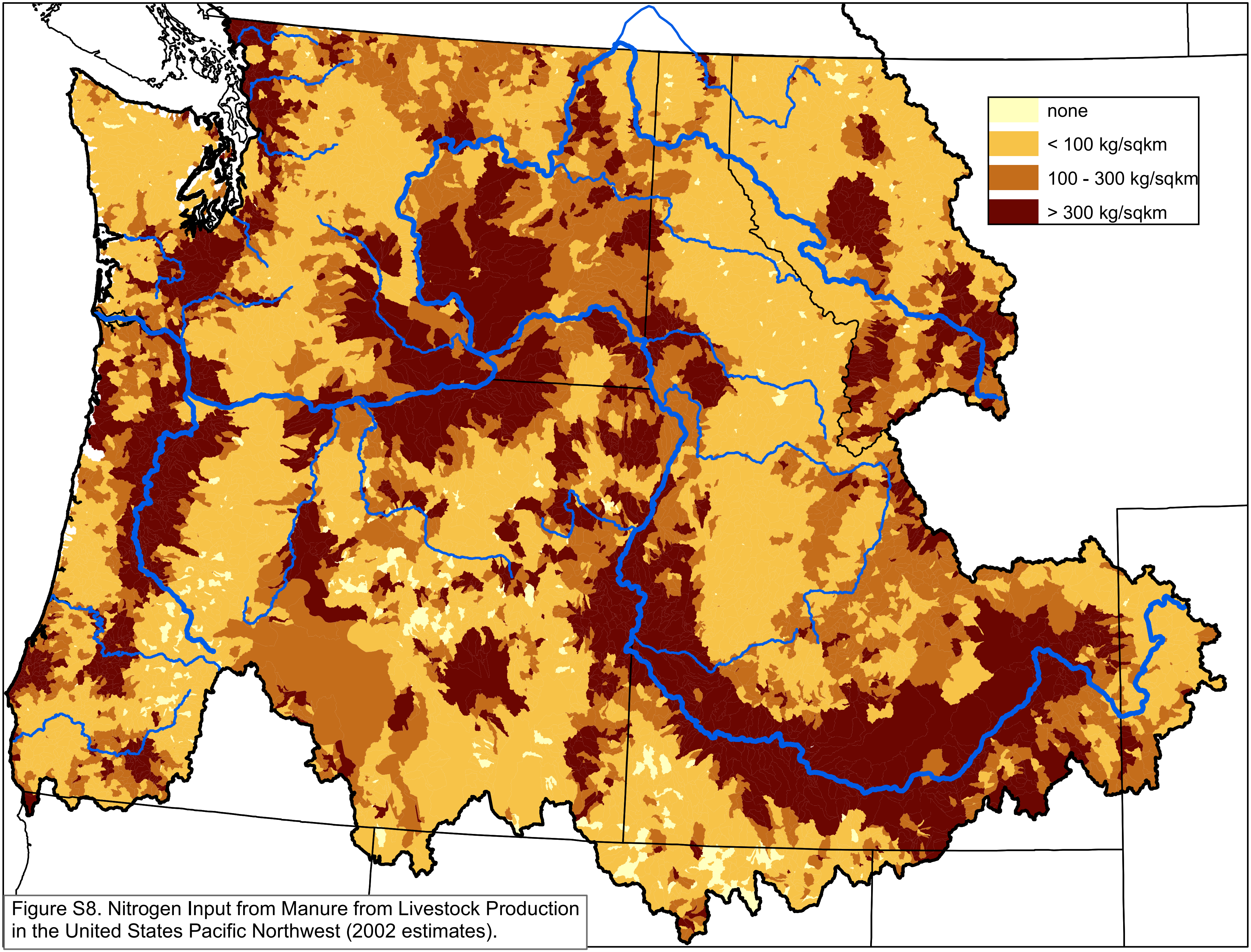

Figure S8. Nitrogen Input from Manure from Livestock Production in the United States Pacific Northwest (2002 estimates).

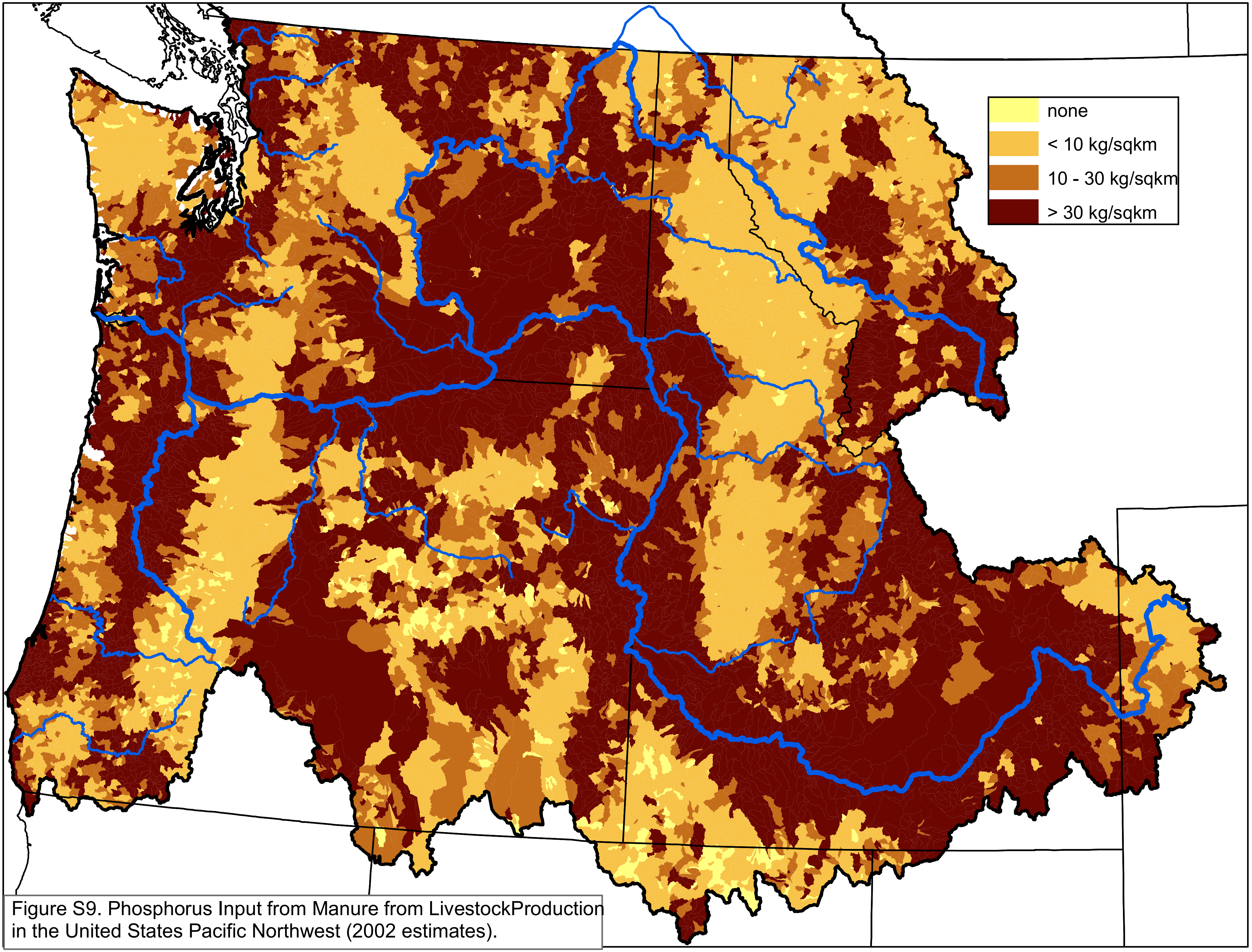

Figure S9. Phosphorus Input from Manure from LivestockProduction in the United States Pacific Northwest (2002 estimates).

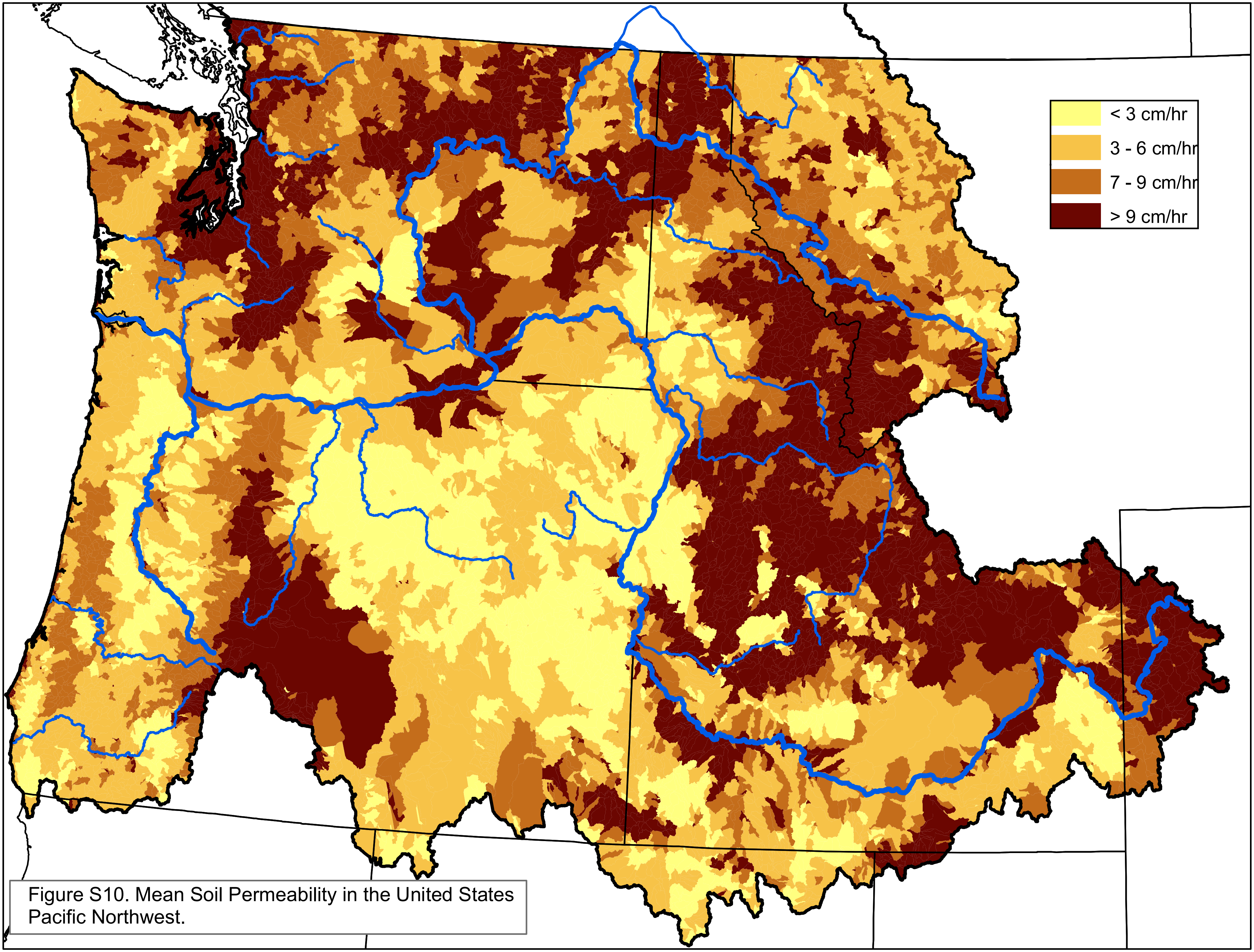

Figure S10. Mean Soil Permeability in the United States Pacific Northwest.

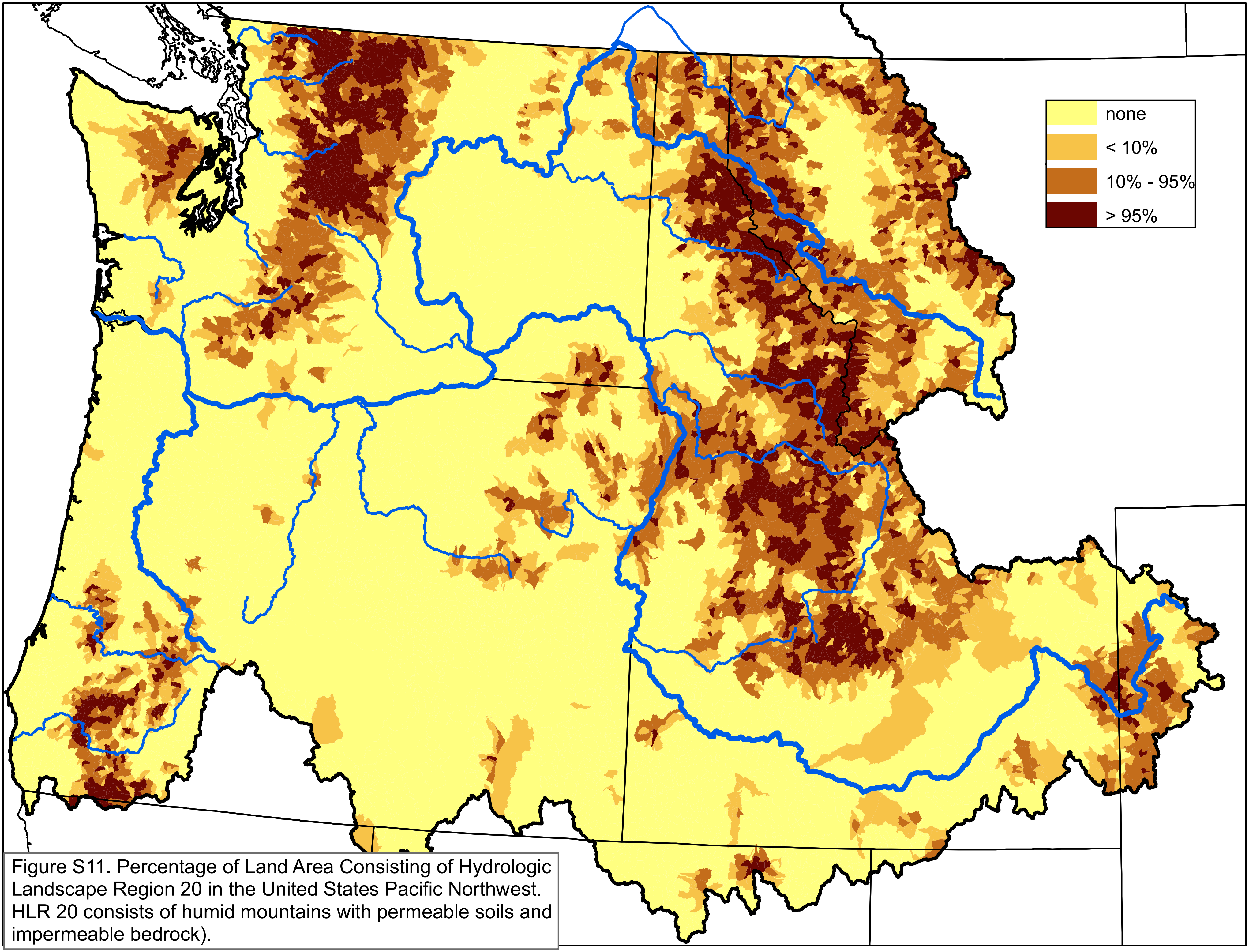

Figure S11. Percentage of Land Area Consisting of Hydrologic Landscape Region 20 in the United States Pacific Northwest. HLR 20 consists of humid mountains with permeable soils and impermeable bedrock).

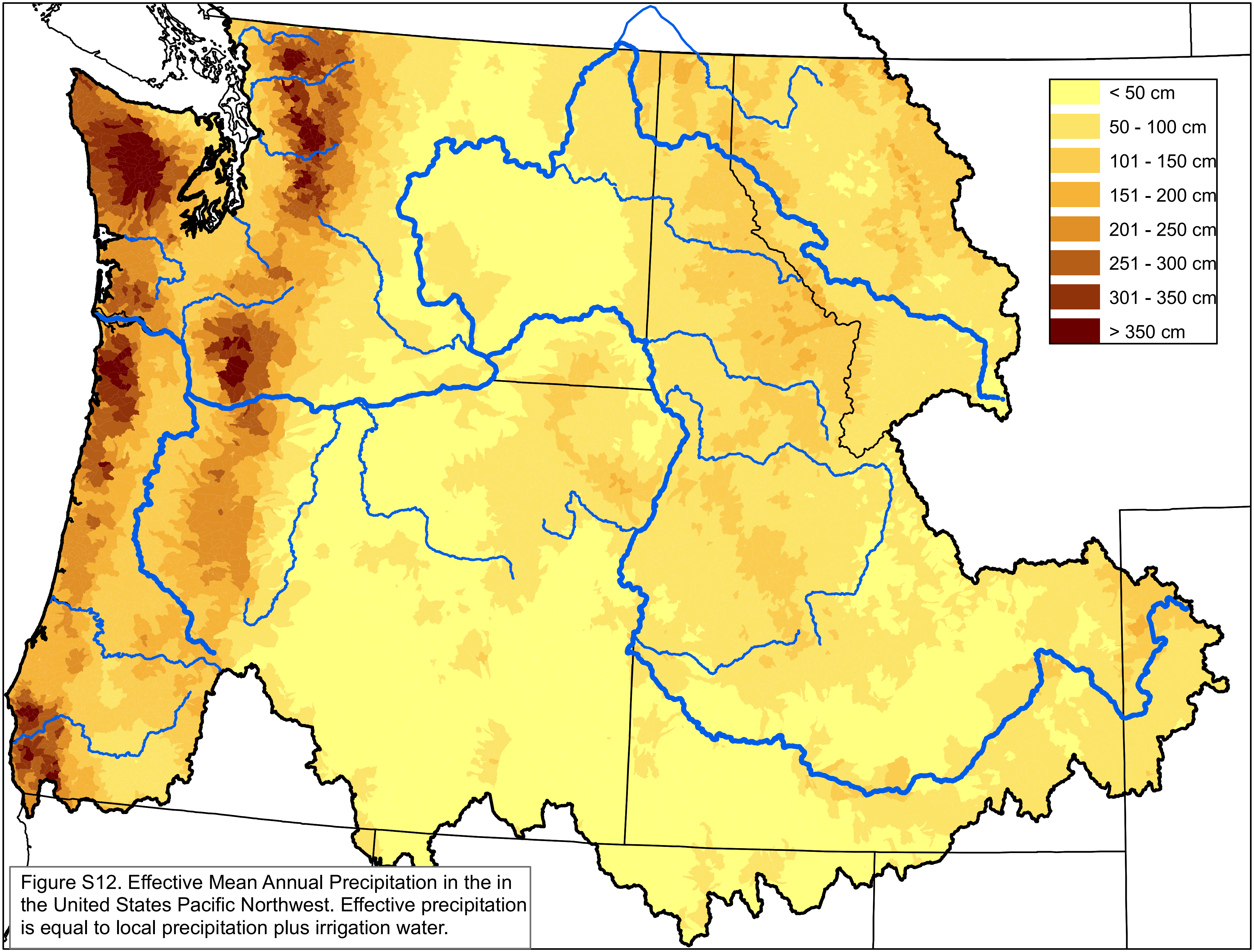

Figure S12. Effective Mean Annual Precipitation in the in the United States Pacific Northwest. Effective precipitation is equal to local precipitation plus irrigation water.

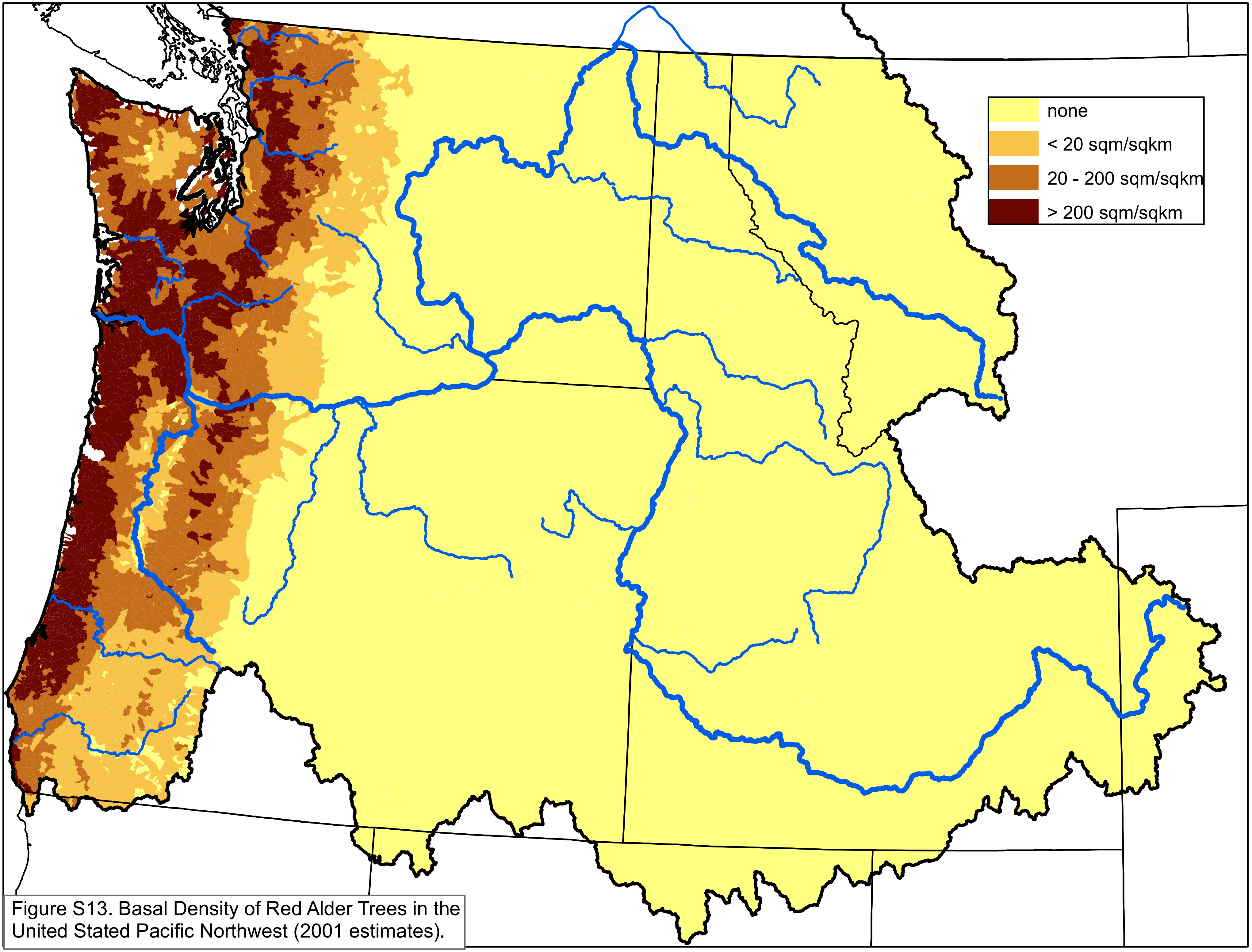

Figure S13. Basal Density of Red Alder Trees in the United Stated Pacific Northwest (2001 estimates).

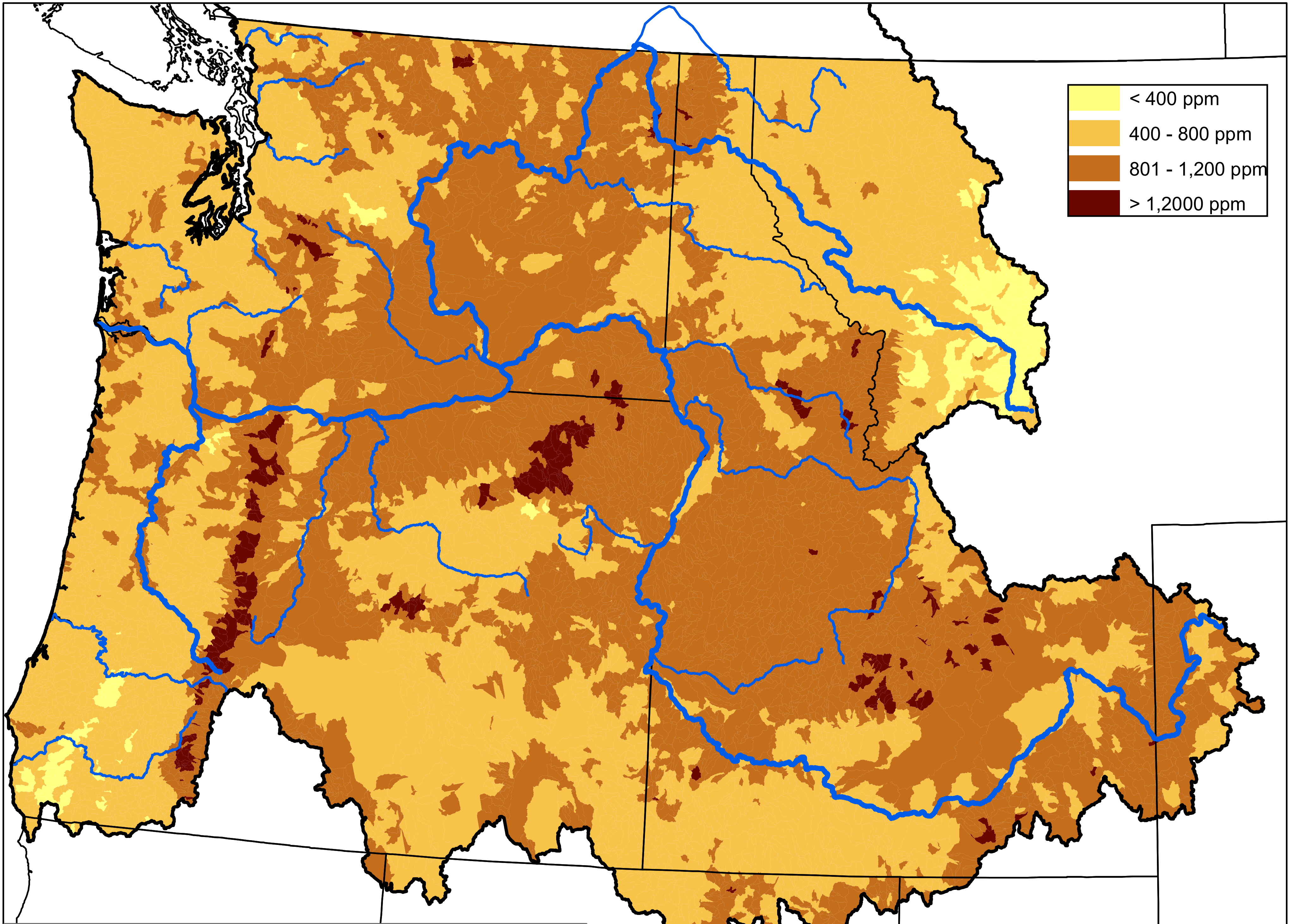

Figure S14. Estimated Concentration of Phosphorus in Surficial Geologic Material in the United States Pacific Northwest.

Figure S15. Measured Stream Load Relative to Predicted Stream Load for the Total Nitrogen SPARROW Model Developed for the United States Pacific Northwest.

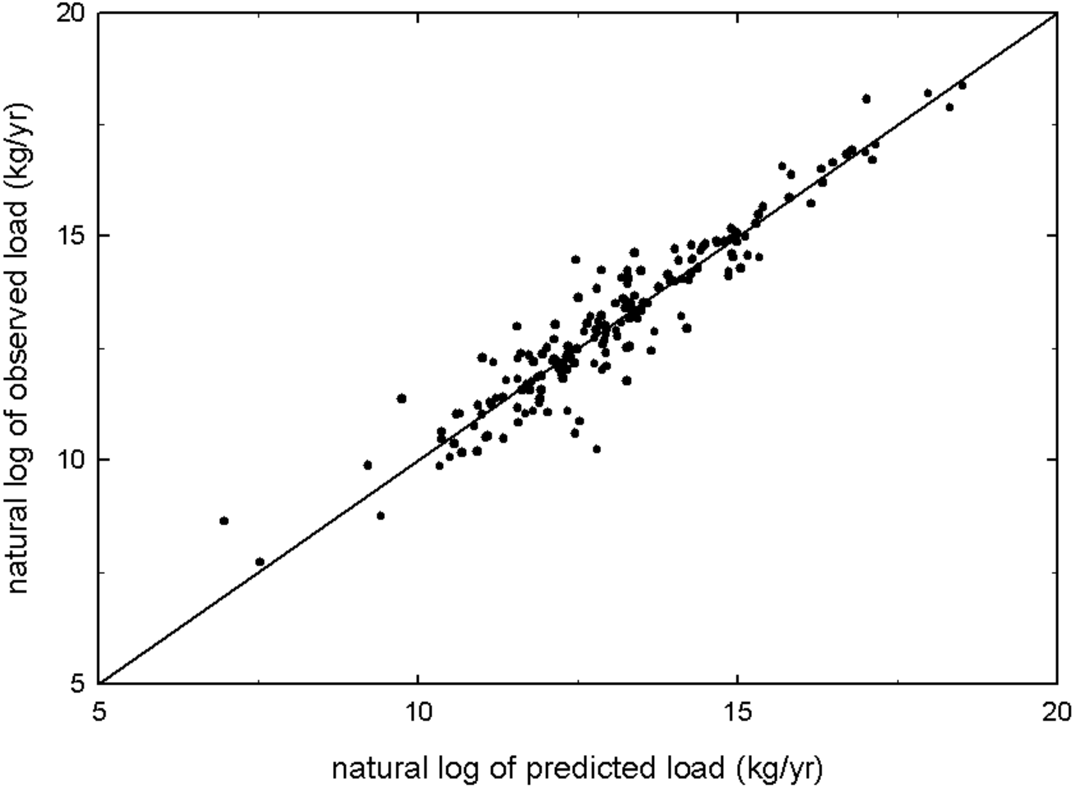

Figure S16. Residual Stream Load Relative to Stream Predicted Load to for the Total Nitrogen SPARROW Model Developed for the United States Pacific Northwest.

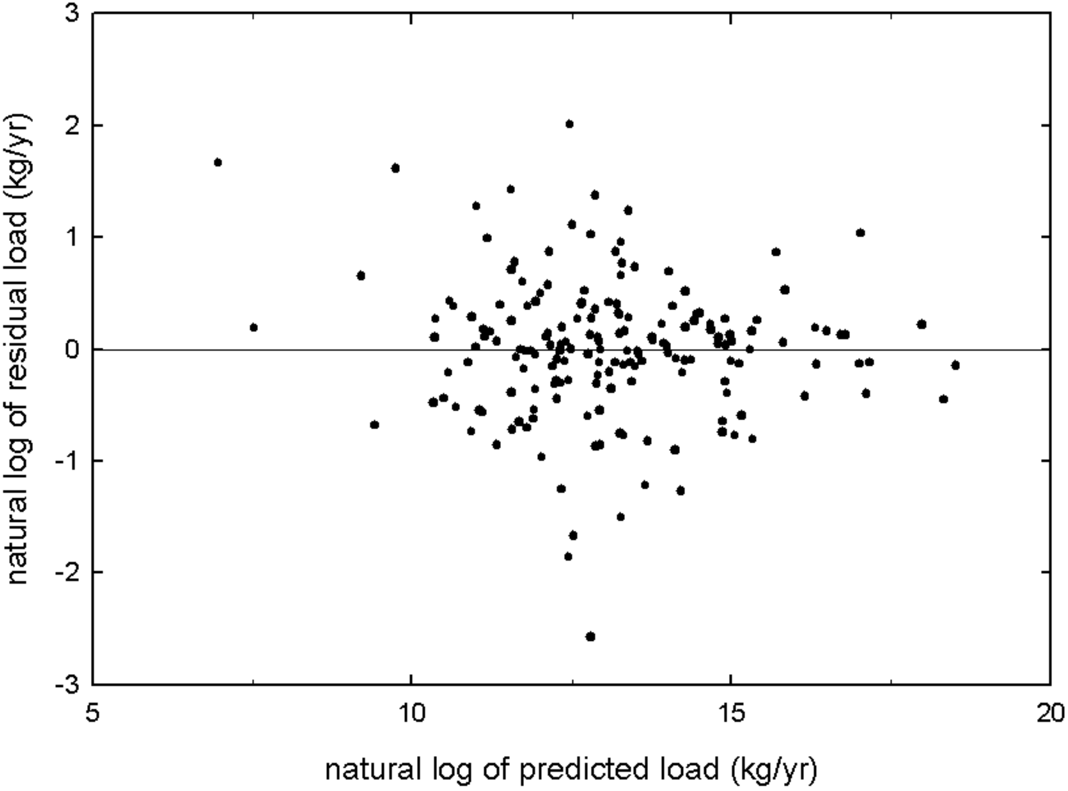

Figure S17. Residual Stream Load Relative to Predicted Yield for the Total Nitrogen SPARROW Model Developed for the United States Pacific Northwest.

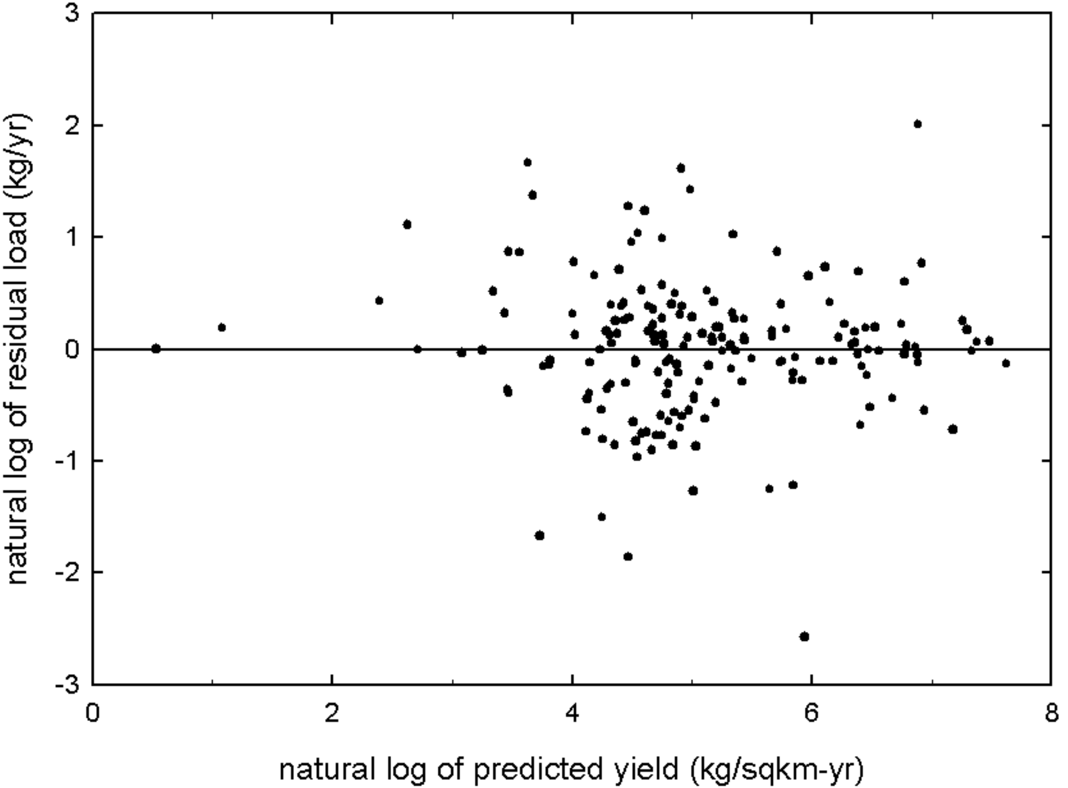

Figure S18. Probability Plot of Standardized Residual Stream Load for the Total Nitrogen SPARROW Model Developed for the United States Pacific Northwest.

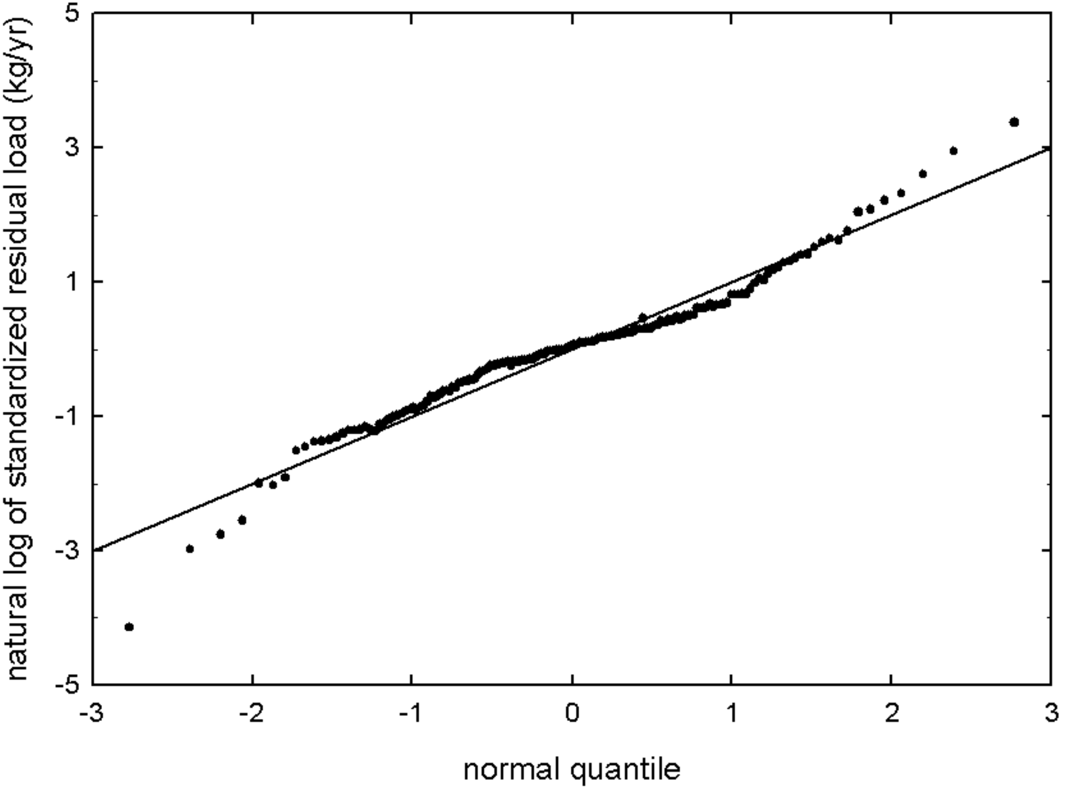

Figure S19. Measured Stream Load Relative to Predicted Stream Load for the Total Phosphorous SPARROW Model Developed for the United States Pacific Northwest.

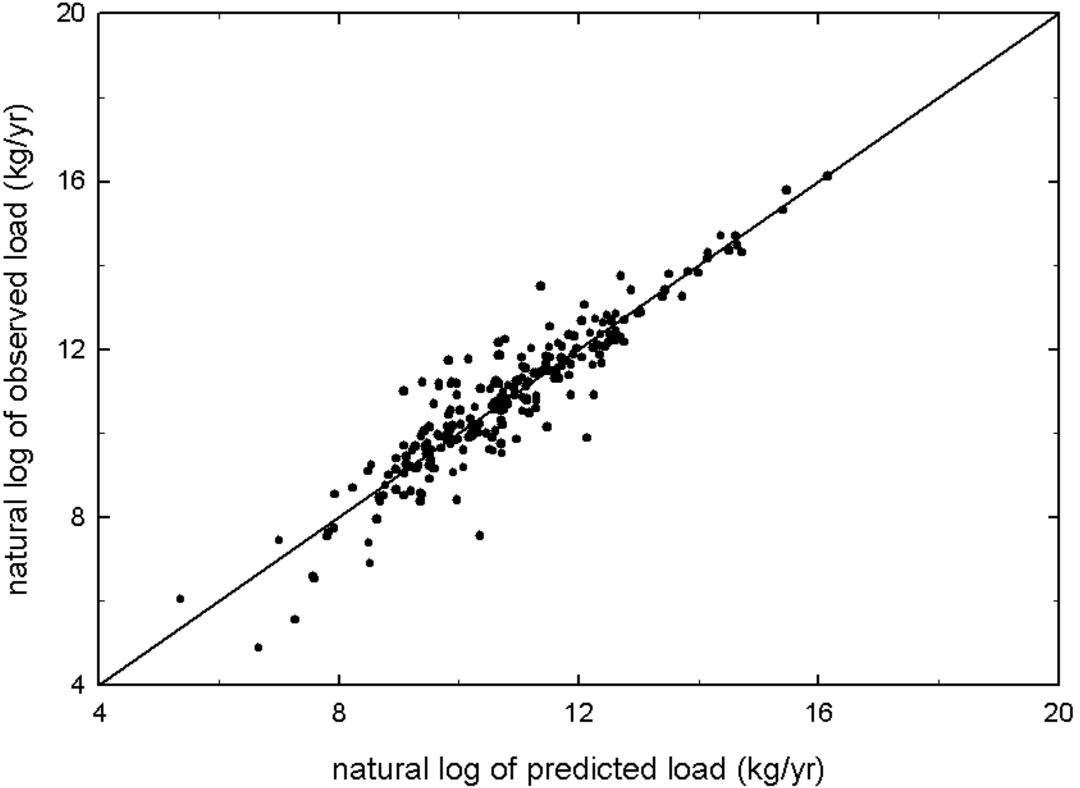

Figure S20. Residual Stream Load Relative to Predicted Stream Load for the Total Phosphorous SPARROW Model Developed for the United States Pacific Northwest.

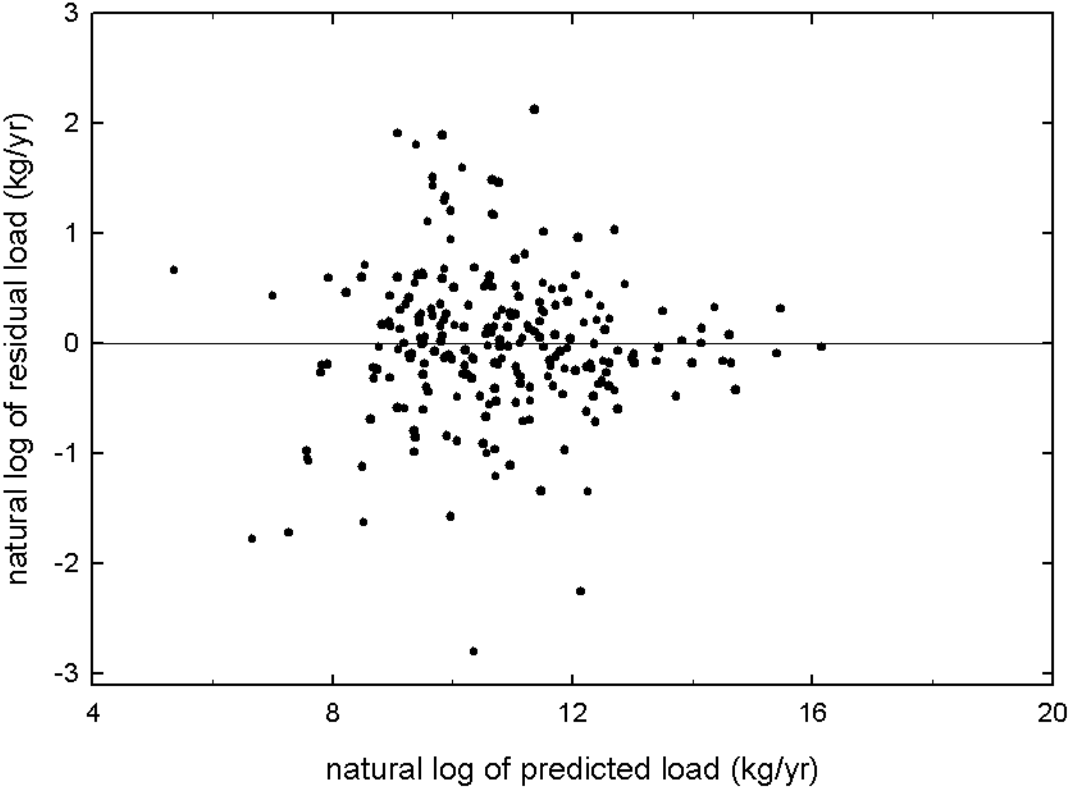

Figure S21. Residual Stream Load Relative to Predicted Yield for the Total Phosphorous SPARROW Model Developed for the United States Pacific Northwest.

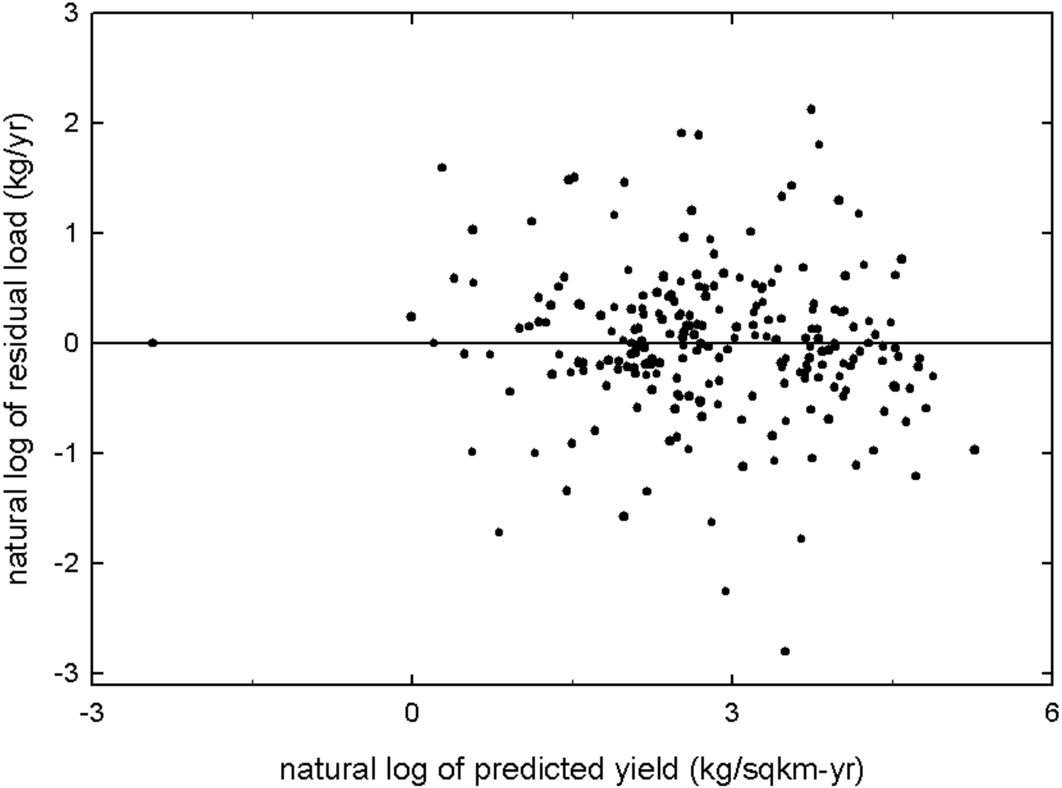

Figure S22. Probability Plot of Standardized Residual Stream Load for the Total Phosphorous SPARROW Model Developed for the United States Pacific Northwest.

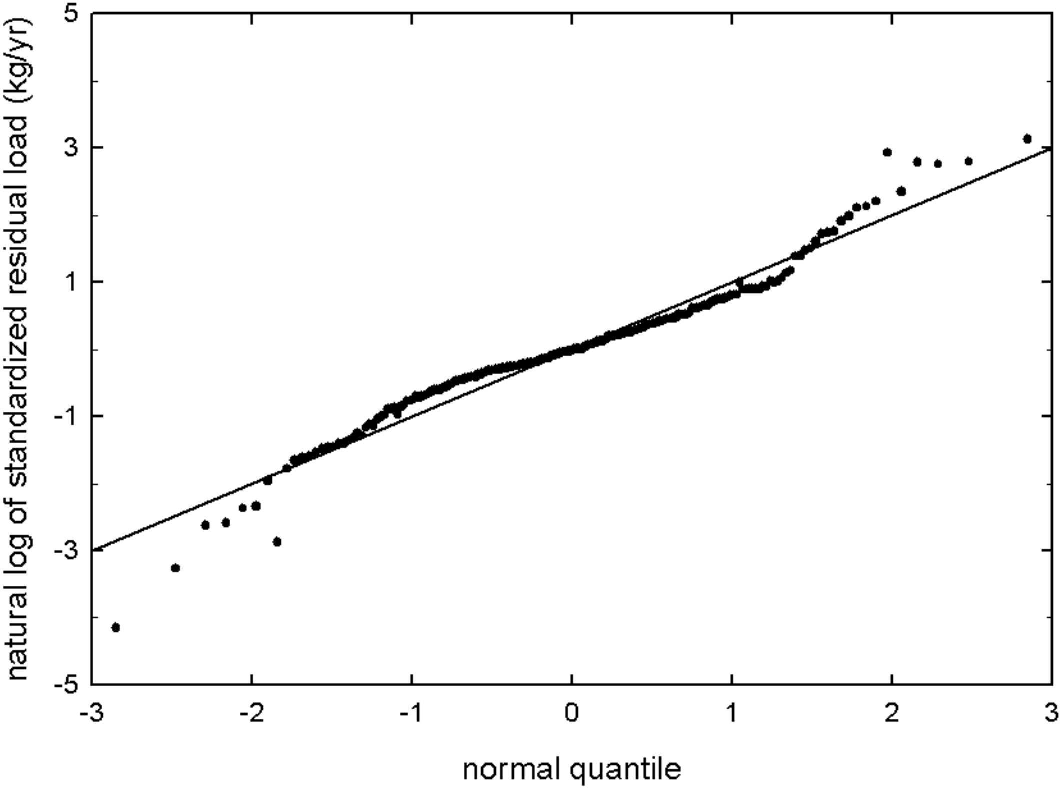

U.S. DEPARTMENT OF THE INTERIOR
U.S. GEOLOGICAL SURVEY

USEPA Level 3 and 4 Ecoregions in the United States Pacific Northwest

Ecoregions—PLATE 1

Wise, D.R. and H.M. Johnson. 2011. Surface-Water Nutrient Conditions and Sources in the United States Pacific Northwest. Journal of the American Water Resources Association (JAWRA).

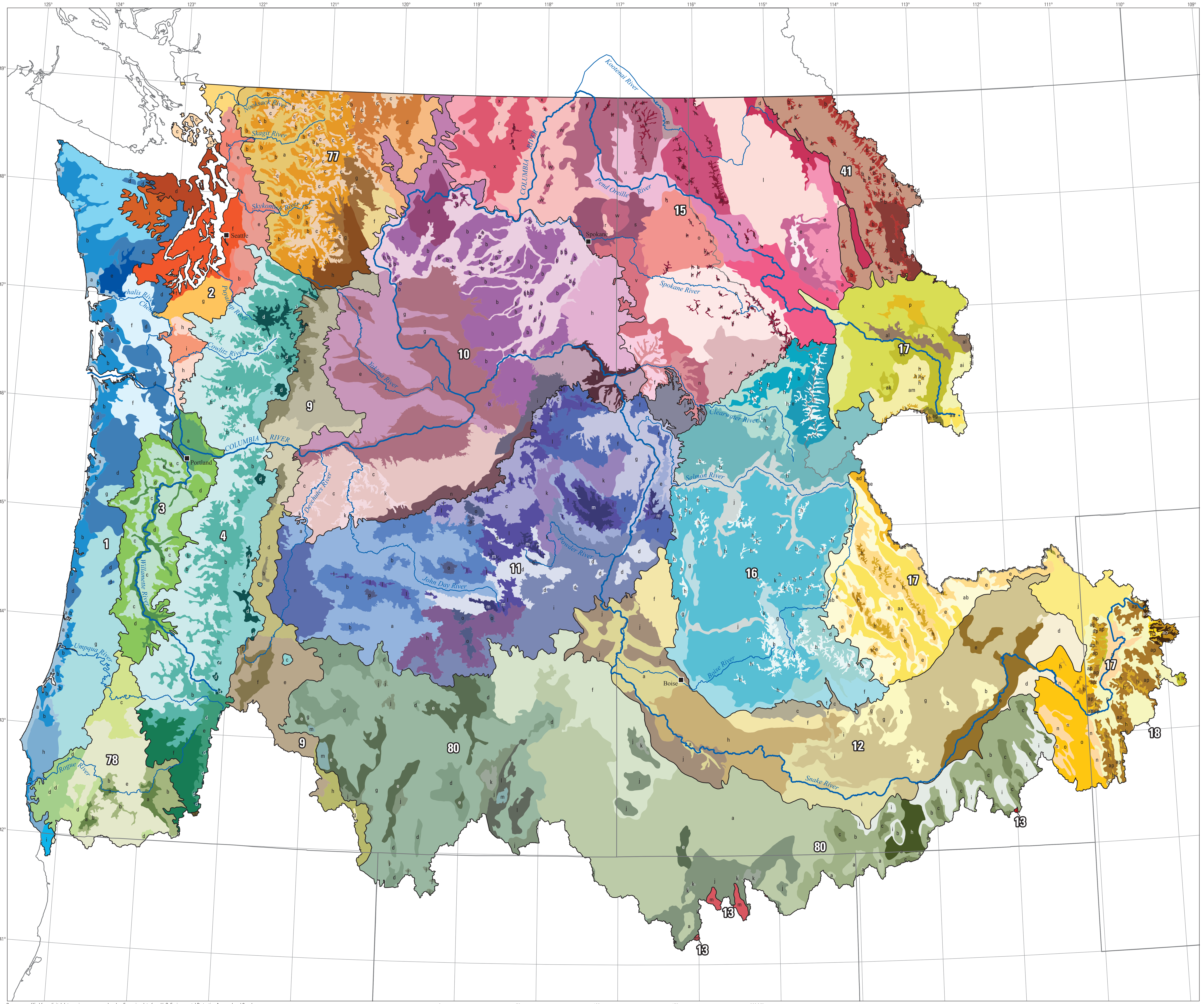

Basemap modified from digital data, various sources and scales. Ecoregion data from U. S. Environmental Protection Agency: Level 3 and Level 4 Ecoregions. Accessed: http://www.epa.gov/wed/pages/ecoregions/level_iii_iv.htm. Coordinate system: Albers Equal Area, SP1=29.5°, SP2=45.5°, CM=-96°, LO=23°. Datum is North American Datum of 1983.

0 50 100 150 200 Miles
0 50 100 150 200 Kilometers

EXPLANATION

1 — U. S. Environmental Protection Agency Level 3 Ecoregion boundary and number

a — Level 4 Ecoregion id code, see chart below

1 Coast Range
- a, Coastal Lowlands
- b, Coastal Uplands
- c, Low Olympics
- d, Volcanics
- e, Outwash
- f, Willapa Hills
- g, Mid-Coastal Sedimentary
- h, Southern Oregon Coastal Mountains
- i, Redwood Zone

2 Puget Lowland
- a, Fraser Lowland
- b, Eastern Puget Riverine Lowlands
- d, Olympic Rainshadow
- e, Eastern Puget Uplands
- f, Central Puget Lowland
- g, Southern Puget Prairies
- h, Cowlitz/Chehalis Foothills
- i, Cowlitz/Newaukum Prairie Floodplains

3 Willamette Valley
- a, Portland/Vancouver Basin
- b, Willamette River and Tributaries Gallery Forest
- c, Prairie Terraces
- d, Valley Foothills

4 Cascades
- a, Western Cascades Lowlands and Valleys
- b, Western Cascades Montane Highlands
- c, Cascade Crest Montane Forest
- d, Cascade Subalpine/Alpine
- e, High Southern Cascades Montane Forest
- f, Southern Cascades

9 Eastern Cascades Slopes and Foothills
- a, Yakima Plateau & Slopes
- b, Grand Fir Mixed Forest
- c, Oak/Conifer Foothills
- d, Ponderosa Pine/Bitterbrush Woodland
- e, Pumice Plateau
- f, Pumice Plateau Basins
- g, Klamath/Goose Lake Basins
- h, Fremont Pine/Fir Forest
- i, Southern Cascade Slope

10 Columbia Plateau
- a, Channeled Scablands
- b, Loess Islands
- c, Umatilla Plateau
- d, Okanogan Drift Hills
- e, Pleistocene Lake Basins
- f, Dissected Loess Uplands
- g, Yakima Folds
- h, Palouse Hills
- i, Deep Loess Foothills
- j, Nez Perce Prairie
- k, Deschutes/John Day Canyons
- l, Lower Snake and Clearwater Canyons
- m, Okanogan Valley
- n, Umatilla Dissected Uplands

11 Blue Mountains
- a, John Day/Clarno Uplands
- b, John Day/ Clarno Highlands
- c, Maritime-Influenced Zone
- d, Melange
- e, Wallowas/Seven Devils Mountains
- f, Canyons and Dissected Highlands
- g, Canyons and Dissected Uplands
- h, Continental Zone Highlands
- i, Continental Zone Foothills
- k, Blue Mountain Basins
- l, Mesic Forest Zone
- m, Subalpine-Alpine Zone
- n, Deschutes River Valley
- o, Cold Basins

12 Snake River Plain
- a, Treasure Valley
- b, Lava Fields
- c, Camas Prairie
- d, Dissected Plateaus and Teton Basin
- e, Upper Snake River Plain
- f, Semiarid Foothills
- g, Eastern Snake River Basalt Plains
- h, Mountain Home Uplands
- i, Magic Valley
- j, Unwooded Alkaline Foothills

13 Central Basin and Range
- i, Malad and Cache Valleys
- m, Upper Humboldt Plains

15 Northern Rockies
- a, Grave Creek Range-Nine Mile Divide
- b, Camas Valley
- c, Flathead Valley
- d, Tobacco Plains
- e, Flathead Hills and Mountains
- f, Grassy Potlatch Ridges
- g, Western Okanogan Semiarid Foothills
- h, High Northern Rockies
- i, Clearwater Mountains and Breaks
- j, Lower Clearwater Canyons
- k, Clark Fork Valley and Mountains
- l, Salish Mountains
- m, Kootenai Valley
- n, Weippe Prairie
- o, Coeur d Alene Metasedimentary Zone
- p, St. Joe Schist-Gneiss Zone
- q, Purcell-Cabinet-North Bitterroot Mountains
- r, Okanogan-Colville Xeric Valleys and Foothills
- s, Spokane Valley Outwash Plains
- t, Stillwater-Swan Wooded Valley
- u, Inland Maritime Foothills and Valleys
- v, Northern Idaho Hills and Low Relief Mountains
- w, Western Selkirk Maritime Forest
- x, Okanogan Highland Dry Forest
- y, Granitic Selkirk Mountains

16 Idaho Batholith
- a, Eastern Batholith
- b, Lochsa Uplands
- c, Lochsa-Selway-Clearwater Canyons
- d, Dry Partly Wooded Mountains
- e, Glaciated Bitterroot Mountains and Canyons
- f, Foothill Shrublands-Grasslands
- g, High Glacial Drift-Filled Valleys
- h, High Idaho Batholith
- i, South Clearwater Forested Mountains
- j, Hot Dry Canyons
- k, Southern Forested Mountains

17 Middle Rockies
- aa, Dry Intermontane Sagebrush Valleys
- ab, Dry Gneissic-Schistose-Volcanic Hills
- ac, Big Hole
- ad, Western Beaverhead Mountains
- ae, Forested Beaverhead Mountains
- ag, Pioneer-Anaconda Ranges
- ah, Eastern Pioneer Sedimentary Mountains
- ai, Elkhorn Mountains-Boulder Batholith
- aj, Eastern Divide Mountains
- ak, Deer Lodge-Philipsburg-Avon Grassy Intermontane Hills and Valleys
- al, Southern Garnet Sedimentary-Volcanic Mountains
- am, Flint Creek-Anaconda Mountains
- ao, Absaroka Volcanic Subalpine Zone
- ap, Absaroka-Gallatin Volcanic Mountains
- ap, High Elevation Rockland Alpine Zone
- ap, Sedimentary Subalpine Zone
- e, Barren Mountains
- g, Mid-elevation Sedimentary Mountains
- h, Alpine Zone
- h, High Elevation Rockland Alpine Zone
- i, Absaroka-Gallatin Volcanic Mountains
- j, Yellowstone Plateau
- k, Granitic Subalpine Zone
- l, Gneissic-Schistose Forested Mountains
- n, Cold Valleys
- o, Partly Forested Mountains
- p, Foothill Potholes
- s, Bitterroot-Frenchtown Valley
- x, Rattlesnake-Blackfoot-South Swan -Northern Garnet-Sapphire Mountains

18 Wyoming Basin
- tc, Sub-irrigated High Valleys

41 Canadian Rockies
- b, Crestal Alpine-Subalpine Zone
- c, Western Canadian Rockies
- d, Southern Carbonate Front
- e, Flathead Thrust Faulted Carbonate-Rich Mountains

77 North Cascades
- a, North Cascades Lowland Forests
- b, North Cascades Highland Forests
- c, North Cascades Subalpine/Alpine
- d, Pasayten/Sawtooth Highlands
- e, Okanogan Pine/Fir Hills
- f, Chelan Tephra Hills
- g, Wenatchee/Chelan Highlands
- h, Chiwaukum Hills and Lowlands
- i, High Olympics

78 Klamath Mountains
- a, Rogue/Illinois Valleys
- b, Oak Savanna Foothills
- c, Umpqua Interior Foothills
- d, Serpentine Siskiyous
- e, Inland Siskiyous
- f, Coastal Siskiyous
- g, Klamath River Ridges

80 Northern Basin and Range
- a, Dissected High Lava Plateau
- b, Semiarid Hills and Low Mountains
- c, High Elevation Forests and Shrublands
- d, Pluvial Lake Basins
- e, High Desert Wetlands
- f, Owyhee Uplands and Canyons
- g, High Lava Plains
- h, Sagebrush-Dominated Valleys
- i, Sagebrush Steppe Valleys
- j, Semiarid Uplands
- k, Partly Forested Mountains
- l, Salt Shrub Valleys
- m, Barren Playas

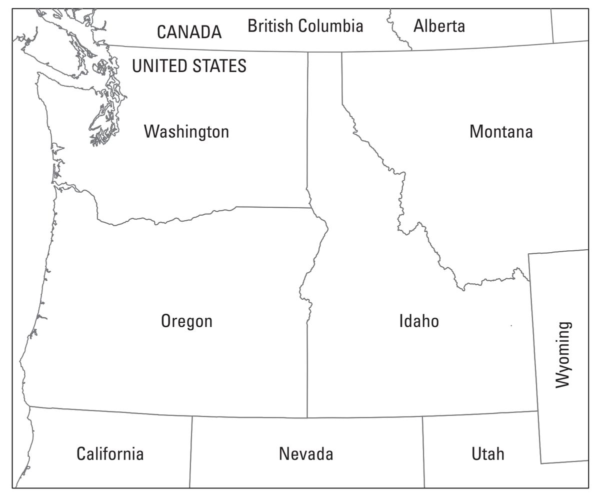

U. S. Environmental Protection Agency Level 3 and Level 4 Ecoregion Boundaries in the United States Pacific Northwest
